# *iFeatureOmega:* an integrative platform for engineering, visualization and analysis of features from molecular sequences, structural and ligand data sets

**DOI:** 10.1093/nar/gkac351

**Published:** 2022-05-07

**Authors:** Zhen Chen, Xuhan Liu, Pei Zhao, Chen Li, Yanan Wang, Fuyi Li, Tatsuya Akutsu, Chris Bain, Robin B Gasser, Junzhou Li, Zuoren Yang, Xin Gao, Lukasz Kurgan, Jiangning Song

**Affiliations:** Collaborative Innovation Center of Henan Grain Crops, Henan Agricultural University, Zhengzhou 450046, China; Center for Crop Genome Engineering, Henan Agricultural University, Zhengzhou 450046, China; Drug Discovery and Safety, Leiden Academic Centre for Drug Research, Einsteinweg 55, Leiden 2333 CC, The Netherlands; State Key Laboratory of Cotton Biology, Institute of Cotton Research of Chinese Academy of Agricultural Sciences (CAAS), Anyang 455000, China; Monash Biomedicine Discovery Institute and Department of Biochemistry and Molecular Biology, Monash University, Melbourne, Victoria 3800, Australia; Monash Biomedicine Discovery Institute and Department of Biochemistry and Molecular Biology, Monash University, Melbourne, Victoria 3800, Australia; Monash Biomedicine Discovery Institute and Department of Biochemistry and Molecular Biology, Monash University, Melbourne, Victoria 3800, Australia; Bioinformatics Center, Institute for Chemical Research, Kyoto University, Kyoto 611-0011, Japan; Monash Data Future Institutes, Monash University, Melbourne, Victoria 3800, Australia; Department of Veterinary Biosciences, Melbourne Veterinary School, The University of Melbourne, Parkville, Victoria 3010, Australia; Collaborative Innovation Center of Henan Grain Crops, Henan Agricultural University, Zhengzhou 450046, China; State Key Laboratory of Cotton Biology, Institute of Cotton Research of Chinese Academy of Agricultural Sciences (CAAS), Anyang 455000, China; Computational Bioscience Research Center (CBRC), Computer, Electrical and Mathematical Sciences and Engineering Division, King Abdullah University of Science and Technology (KAUST), Thuwal 23955, Saudi Arabia; Department of Computer Science, Virginia Commonwealth University, Richmond, VA, USA; Monash Biomedicine Discovery Institute and Department of Biochemistry and Molecular Biology, Monash University, Melbourne, Victoria 3800, Australia; Monash Data Future Institutes, Monash University, Melbourne, Victoria 3800, Australia

## Abstract

The rapid accumulation of molecular data motivates development of innovative approaches to computationally characterize sequences, structures and functions of biological and chemical molecules in an efficient, accessible and accurate manner. Notwithstanding several computational tools that characterize protein or nucleic acids data, there are no one-stop computational toolkits that comprehensively characterize a wide range of biomolecules. We address this vital need by developing a holistic platform that generates features from sequence and structural data for a diverse collection of molecule types. Our freely available and easy-to-use *iFeatureOmega* platform generates, analyzes and visualizes 189 representations for biological sequences, structures and ligands. To the best of our knowledge, *iFeatureOmega* provides the largest scope when directly compared to the current solutions, in terms of the number of feature extraction and analysis approaches and coverage of different molecules. We release three versions of *iFeatureOmega* including a webserver, command line interface and graphical interface to satisfy needs of experienced bioinformaticians and less computer-savvy biologists and biochemists. With the assistance of *iFeatureOmega*, users can encode their molecular data into representations that facilitate construction of predictive models and analytical studies. We highlight benefits of *iFeatureOmega* based on three research applications, demonstrating how it can be used to accelerate and streamline research in bioinformatics, computational biology, and cheminformatics areas. The *iFeatureOmega* webserver is freely available at http://ifeatureomega.erc.monash.edu and the standalone versions can be downloaded from https://github.com/Superzchen/iFeatureOmega-GUI/ and https://github.com/Superzchen/iFeatureOmega-CLI/.

## Graphical Abstract

**Graphical Abstract:**
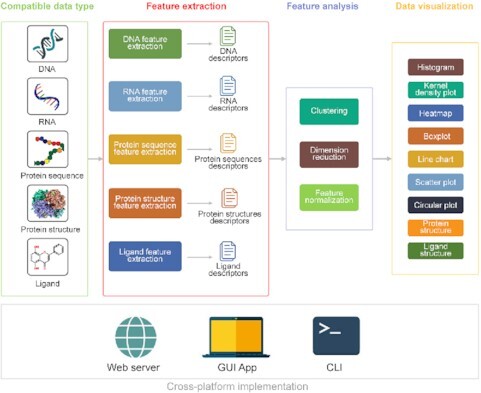
*iFeatureOmega* provides three interfaces including the web server, locally executable command line interface and graphical user interface, to perform feature extraction, analysis and visualization of biological sequences, structures and ligands.

## INTRODUCTION

The speed and affordability of high-throughput sequencing techniques have led to massive influx and accumulation of molecular data (1–3). However, curation and analysis of these data could be challenging due to lack of computational methods that facilitate visualization and encoding of the raw data into features that are suitable for statistical data analysis and development of predictive models (4). The encoding is particularly crucial for machine-learning and deep-learning techniques that are increasingly being used to predict key structural and functional properties of nucleic acid and protein sequences (5–8). These studies enhance annotation of genomic and proteomic datasets, indirectly improving understanding of biological processes, pathways and molecular functions across cells, tissues and organisms (9–11).

Efficient and systematic encoding of features that represent different molecule types (e.g. nucleic acids, proteins and small ligands) and which cover different characteristics including sequence, three-dimensional structure and binding-partners, is challenging and vital to develop high quality machine-learning models (4,7). This motivates demand for reliable and accessible tools for feature engineering, extraction, calculation, analysis and visualization from molecular sequences and structures. Many feature engineering tools that target DNA, RNA, proteins and ligands were released in recent years. They include PseAAC (12), PROFEAT (13), PseAAC-Builder (14), PyDPI (15), ChemoPy (16), propy (17), RDKit (18), PseAAC-General (19), Rcpi (20), ProFET (21), protr/ProtrWeb (22), BioTriangle (23), repRNA (24), POSSUM (25), PseKRAAC (26), iFeature (27), PyFeat (28), Seq2Feature (29), MRMD2.0 (30) and MathFeature (31). Besides these feature engineering tools, several platforms for the development of machine learning predictors, including BioSeq-Analysis2.0 (32), PFeature (33), iLearn (34) and iLearnPlus (5), also provide feature extraction facilities. These computational toolkits have been employed in numerous bioinformatics and cheminformatics projects, with just a few examples that cover identification and prediction of mutational effects (35), protein–protein interaction hotspots (36), drug-target interactions (37), protein crystallization propensity (38), DNA-binding sites (39) and DNA-binding proteins (40), protein families (41) and/or DNA/RNA/protein modifications (42–45). The PseAAC (12) web server appears to be the earliest tool. It covers protein feature engineering, focusing on encoding features that rely on the pseudo amino acid composition. We highlight two other well-established early tools, Propy (17) (in Python) and Rcpi (20) (in R/Bioconductor), which facilitate calculation of a large numbers of structural and physicochemical features from protein sequences. More recent platforms expand the coverage to extract features from other types of molecules. For instance, PyFeat (28) is a Python-based feature generation tool for DNA, RNA and protein sequences; Seq2Feature (29) calculates protein and DNA sequence-based descriptors; and BioTriangle (23) provides representations for ligands/chemicals and protein, DNA and RNA sequences.

While being often utilized and useful, the existing feature engineering tools have some limitations (Table 1). First, most of the current tools calculate features for one of the molecules. Only BioTriangle covers DNA, RNA, ligands and protein sequences, however, it does not consider protein structures. MathFeature (31) and some of the recent machine learning platforms, such as BioSeq-Analysis2.0 (32), iLearn (34) and iLearnPlus (5), provide a relatively rich collection of feature sets for nucleic acids and proteins, outperforming the older feature engineering tools, however, they do not consider ligands and protein structures, except for PFeature (33) that considers only protein sequences and structures. A few current tools, including PIC (46), PDBparam (47) and PFeature, encode features from protein structure, facilitating important applications, such as rational drug development (48) and prediction of protein functions (49–52).

**Table 1.** Comparison of existing state-of-the-art computational toolkits for feature engineering, extraction, calculation, analysis and visualization. Tools are sorted chronologically

Tools	Coverage of different molecule types					Performs feature analysis	Performs data visualization	Available interfaces			Ref.
	DNAs	RNAs	Protein sequences	Ligands	Protein structures			Web server	CLI stand-alone	GUI stand-alone	
PIC	×	×	×	×	√	×	√	√	×	×	(46)
PseAAC	×	×	√ (3)	×	×	×	×	√	×	×	(12)
PROFEAT	×	×	√ (11)	√ (1)	×	×	×	√	×	×	(13)
PseAAC-Builder	×	×	√ (3)	×	×	×	×	×	√	√	(14)
PyDPI	×	×	√ (14)	√ (13)	×	×	×	×	√	×	(15)
ChemoPy	×	×	×	√ (19)	×	×	×	×	√	×	(16)
Propy	×	×	√ (13)	×	×	×	×	×	√	×	(17)
PseAAC-General	×	×	√ (13)	×	×	×	×	×	√	×	(19)
Rcpi	×	×	√ (10)	√ (8)	×	×	×	×	√	×	(20)
Protr/ProtrWeb	×	×	√ (22)	×	×	×	×	×	√	×	(22)
BioTriangle	√(14)	√(14)	√(14)	√(18)	×	×	×	√	×	×	(23)
PDBparam	×	×	×	×	√(4)	×	×	√	×	×	(47)
repRNA	×	√ (11)	×	×	×	×	×	√	×	×	(24)
PseKRAAC	×	×	√ (16)	×	×	×	×	√	×	×	(26)
iFeature	×	×	√ (53)	×	×	√(10)	√(2)	√	√	×	(27)
PyFeat	√(13)	√ (13)	√(9)	×	×	×	×	×	√	×	(28)
Seq2Feature	√(1)	√(1)	√ (4)	×	×	×	×	√	×	×	(29)
BioSeq-Analysis2.0*	√(36)	√(27)	√(53)	×	×	√(2)	√(1)	√	√	×	(32)
PFeature*	×	×	√	×	√	×	×	√	√	×	(33)
iLearn*	√(26)	√(18)	√(53)	×	×	√(15)	√(3)	√	√	×	(34)
iLearnPlus*	√(46)	√(35)	√(66)	×	×	√(**20**)	√(7)	√	×	√	(5)
MathFeature	√(30)	√(30)	√(12)	×	×	×	×	×	√	√	(31)
*iFeatureOmega*	√(**49**)	√(**37**)	√(**71**)	√(**18**)	√(**14**)	√(15)	√(**9**)	√	√	√	-

Note: *the tool is a machine-learning platform. ‘X’ means that the function is unavailable. Numbers in the brackets denote the numbers of different feature sets, or analysis/visualization options.

Second, virtually none of the existing feature engineering tools, except for iFeature, supports analysis of the resulting features. Relevant tasks include feature clustering, dimensionality reduction and normalization. These utilities are vitally important to ensure efficiency and quality of the subsequent applications of features. For example, while BioTriangle (23) extracts a diverse collection of descriptors for ligands and protein, DNA and RNA sequences, it does not offer support to cluster, select or normalize these features, forcing users to resort to using other software for this purpose. However, clustering and feature selection are fundamental to many bioinformatics applications and have been widely used (53–55).

Third, in addition to the feature calculation and analysis, users would benefit from visualization capabilities. This may include visualization of the feature values and statistical characteristics, such as distributions. These visual representations assist in screening and validating features (56). Our analysis (Table 1), reveals that only one feature engineering tool, iFeature, and a few machine learning platforms (BioSeq-Analysis2.0, iLearn and iLearnPlus) provide visualization facilities.

Fourth, very few of the published tools provide graphical user interface (GUI). This makes it rather difficult for non-coders and less computer savvy users, including structural biologists and biochemists, to use these tools. Majority of the existing tools must be used with the command line interface (CLI), which requires knowledge of a specific programming language. Web servers and local executable GUI solve these challenges by providing point-and-click interfaces and an easy-to-follow process. However, web servers usually constraint the size of input data and could be offline or heavily used at times, resulting in long delays. Thus, the desired solution is to provide a wide range of options including CLI to cater to experienced bioinformaticians and programmers, web server for users who may not be able to run software on their local hardware and need ad hoc access, and GUI to serve users who have limited programming background but would like to run the analysis locally. We note that we have experience and history providing related platforms including iLearnPlus (5), iFeature (27) and iLearn (34).

We present *iFeatureOmega* platform that overcomes the above limitations and challenges. *iFeatureOmega* produces 189 feature sets and covers analysis and visualization of DNA, RNA, proteins and ligands. To compare, the largest number of feature sets produced by the current feature engineering tools is 72 for MathFeature and among the machine learning platforms is 147 for iLearnPlus (Table 1). More importantly, iLearnPlus and MatchFeature consider only DNA, RNA and proteins. Our platform integrates 15 feature analysis methods including ten clustering, three dimensionality reduction and two feature normalization algorithms. It provides nine types of interactive plots, including histograms, boxplots, scatters and three-dimensional structures, to facilitate visualization of statistical summaries for the generated features. In contrast to the published feature engineering and machine learning tools (Table 1), *iFeatureOmega* provides a full spectrum of interfaces including the web server and locally executable CLI and GUI. The web server can be accessed through http://ifeatureomega.erc.monash.edu, and the GUI and CTL versions can be downloaded at: https://github.com/Superzchen/iFeatureOmega-GUI and https://github.com/Superzchen/iFeatureOmega-CLI, respectively.

## METHODS

### Features representing amino acid sequences

To describe protein sequences, *iFeatureOmega* incorporates ten categories of feature sets which are widely applied in modern bioinformatic investigations (Supplementary Table S1). They include the amino acid composition, grouped amino acid composition, autocorrelations, quasi-sequence-order, pseudo-amino acid composition, residue representation, physicochemical property, BLOSUM matrix, *Z*-scale index and similarity-based descriptor. The ‘amino acid composition’ category calculates 12 different types of composition features for a given protein/peptide sequence, while ‘grouped amino acid composition’ clusters amino acids based on the calculation of the composition measures for the amino acids in a given subgroup. To compute the autocorrelations and cross-covariance feature sets, *iFeatureOmega* covers six correlation and covariance measures for individual amino acid sequences, summarized in the ‘autocorrelations’ category. Two sequence order-based features can also be calculated by *iFeatureOmega* in the ‘quasi-sequence-order’ category. Similar to the amino acid composition, pseudo amino acid composition uses a series of measures to characterize protein/peptide sequences, but pseudo amino acid composition incorporates additional information, such as the correlation between residues within a distance threshold, to better describe local sequence patterns (57). *iFeatureOmega* also provides measures including amphiphilic pseudo-amino acid composition (APAAC), and 16 types of pseudo *K*-tuple reduced amino acid compositions. The sixth group includes 16 types of residue-level feature sets, where each amino acid is represented by a vector of a fixed length. To better represent the physicochemical properties of a particular protein/peptide sequence, *iFeatureOmega* also refers to the AAindex database (58), which contains 556 indices that quantify physicochemical properties of individual amino acids, such as alpha-CH chemical shifts and hydrophobicity. The BLOSUM (BLOcks SUbstitution Matrix) (59) is a widely-used matrix to show the relatedness of amino acids in sequence alignments, reflecting evolutionary divergence. *iFeatureOmega* incorporates BLOSUM62 (59) to build such matrices. Finally, the ninth category is the *Z*-scales, in which each amino acid is represented by five physicochemical descriptor variables; this feature set is inspired by the *Z*-scales index that was developed by Sandberg *et al.* (60). Finally, the similarity-based descriptor quantifies similarity between sequences based on the nearest neighbor approach.

### Features representing nucleic acid sequences

Eight major categories of features can be encoded from DNA and RNA sequences with *iFeatureOmega*. They include nucleic acid composition, pseudo nucleic acid composition, position-specific encoding of *n*-nucleotides, electron-ion interaction pseudopotential (EIIP), autocorrections and cross-covariance, physiochemical property, mutual information, and similarity-based descriptor (Supplementary Table S2). The ‘Nucleic acid composition’ features quantify the frequencies of nucleotides in a sequence. The ‘position-specific of *n*-nucleotides’ group provides nucleic acid feature vectors calculated using more advanced nucleotide compositions, such as dinucleotide binary encoding and position-specific encoding of the four nucleotides. EIIP refers to the energy of delocalized electrons in nucleotides (61,62). We also provided autocorrelations and cross-covariance features for nucleotides, which represent the statistical pattern of nucleic acid sequences. Regarding the physicochemical properties of nucleotides, *iFeatureOmega* calculates di-/tri-nucleotide physicochemical properties using a recently published approach (63). The sixth feature group is multivariate ‘mutual information’, which quantifies correlations between nucleotide pairs (64). The seventh feature group consists of six types of pseudo nucleic acid compositions (65,66), such as pseudo *k*-tuple composition and parallel correlation pseudo dinucleotide composition, complementing the above nucleic acid composition features. The last feature group is similarity-based, and it calculates similarity between a query nucleotide sequence and the other nucleotide sequences using the nearest neighbor method.

### Features representing ligand information

Ligand features quantify several different aspects of their chemical structures (23). The *iFeatureOmega* platform covers more than ten feature groups for the ligand data including constitution, topology, connectivity, *E*-state (topological and electronic information linked to atoms), Kappa (molecular shape descriptors), bask, burden, Kappa, autocorrelations, charge (electronic measure for a complete molecule or specific regions within a molecule), molecular property descriptors, pharmacophore, MOE-type and fingerprints (Supplementary Table S3). The molecular constitution descriptors characterize composition of chemical elements and chemical bonds, path length, hydrogen bond-acceptor, and donator in the constitution module. The topological descriptors, which are calculated directly from the ligand structure, quantify key topological aspects including molecular connectivity and valence connectivity for different path-orders, cycle, or cluster size. In addition, the E-state, bask, burden, pharmacophore and charge can also be calculated and used to represent the physiochemical properties of a ligand. We also provided three types of autocorrelation-based features including geary, moran and moreau-broto autocorrelations. The molecular descriptors focus on the chemical structures of ligands while the Molecular Operating Environment (MOE)-type descriptors represent the topological, structural, and physiochemical properties. In *iFeatureOmega*, the MOE-type descriptors are computed from the connection table information based on atomic contributions to Van der Waals surface area, log *P*, molar refractivity, partial charge and *E*-state value. Finally, we incorporated fingerprint calculations to facilitate rapid screening, string representations and structural similarity measurements ligands and similar chemicals (67). We covered multiple fingerprint types including MACCS, morgan and *E*-state.

### Features representing protein structure

The three-dimensional structure of a protein is useful to decode and study protein function, following the ‘structure-to-function’ paradigm (68). Accurate representation of a protein structure is therefore critically important to analyze and predict its function and functional sites. Functional sites are microenvironments within the structure that can be defined by both three-dimensional and local neighborhood locations and which are involved in a particular function (69). In *iFeatureOmega*, we implemented seven feature groups that capture the microenvironments—ranging in scale from atoms to residues and to secondary structure elements; in total 14 feature encoding schemes. These groups include amino acids composition, grouped amino acid composition, secondary structure, half sphere exposure, residue depth, atom composition and network-based index (Supplementary Table S4).

The first group includes two types of feature sets that quantify the amino acid composition: AAC_type1 and AAC_type2. Here, the target sites (microenvironments) are defined by a three-dimensional position and a radius defining the neighborhood. Shells are formed around each target site (Supplementary Figure S1) and the frequency of each amino acid type is calculated for each shell (AAC_type1) and for cumulative shells (AAC_type2). In the secondary group, the 20 amino acid types are further categorized into five classes, according to their physicochemical properties (hydrophobicity, charge and molecular size). Then, the frequency of each amino acid group is calculated in the same way as for the first group. The ‘secondary structure’ feature group encodes multiple features based on secondary structural elements around a target residue. For feature sets in this group, ‘SS3’ considers three types of secondary structural elements (i.e. helix, β-strand, and β-turns and -loops (70)), while ‘SS8’ considers eight types of secondary structural elements (i.e. α-helix, isolated β-bridge residue, strand, 3–10 helix, Π-helix, turn, bend and other (70)). The type1 features calculate the frequency of each type of SSE in each shell (i.e. SS3_type1 and SS8_type1) while type2 features quantify the frequency of each SSE type in cumulative shells (i.e. SS3_type2 and SS8_type2). *iFeatureOmega* also calculates the half sphere exposure and residue depth as descriptors for each residue in a given structure. Half sphere exposure (HSE) is a 2D measure of solvent exposure which counts the number of Cα atoms around a residue in the direction of its side chain and in the opposite direction (within a radius of 13 Å) (71). HSE compliments the residue depth measure (72), which is calculated as an average distance of a residue's atoms from the solvent accessible surface (73). The ‘atom composition’ feature group includes two types of encoding schemes that are computed at the atomic level. For the feature sets in this group, the target atom is specified as a three-dimensional position and a radius defining its neighborhood, and shells are formed around each target atom (Supplementary Figure S1). Then, frequency of each atom type is calculated in each shell (i.e. AC_type1) and in cumulative shells (i.e. AC_type2). Finally, the ‘network-based index’ feature set encodes residues in the context of a network that represents placement of residues in the space defined by the protein structure. These features quantify the degree, degree centrality, betweenness, clustering coefficient, closeness, and centrality values.

### Feature analysis

Feature analysis is an important step to examine and understand relationships between features. This is critically important to guide the process of constructing the subsequently developed models and to assist with evaluation of performance of these models. To this end, *iFeatureOmega* supports three major types of approaches for feature analysis using 15 distinct algorithms. We covered ten methods for feature clustering, three for dimensionality reduction, and two for normalization (Supplementary Table S5). Feature clustering aims to group similar molecules (i.e. DNA, RNA, protein or ligand), which are encoded by a specific set of features. Upon the completion of clustering, molecules are grouped, and each group is assigned a cluster identification code (ID). The feature clustering results are displayed in a scatter plot. Feature dimensionality reduction approaches transform the high-dimensional feature representation to a low-dimensional space, with the aim to retain the most informative features for the subsequent model construction. Supplementary Table S5 lists specific clustering and dimensionality reduction algorithms that are included in *iFeatureOmega*. They include popular methods, such as *k*-means (74), DBSCAN (75) and principal component analysis (PCA) (76). Finally, feature normalization is used to rescale feature values to a specific range so that they are consistent across different feature sets that *iFeatureOmega* generates. We implemented two commonly used normalization algorithms, the *Z*-score and Min–Max normalizations. For *Z*-score normalization, feature values are rescaled to a normal distribution with a mean of 0 and a standard deviation of 1. While for Min-Max normalization, feature values are scaled to the range between 0 and 1.

### Data visualization

*iFeatureOmega* offers various plots that facilitate visualization, interpretation, and analysis of the engineered features. They include histograms, kernel density plots, heatmaps, boxplots, line charts and circular plots (Supplementary Table S6). Histograms and kernel density plots are particularly useful for the visualization of distributions of feature values. Histograms are distribution where feature values are grouped into discrete intervals while kernel density plots produce smooth curves that represent probability density functions and they are best suited for continuous features. The heatmaps provide a different and complementary perspective where distributions of the feature values are set against samples/molecules, i.e. rows and columns correspond to samples and features, respectively. The boxplots and the line charts succinctly summarize distribution of individual features where boxplots, which rely on medians and quartiles, allow for efficient examination and comparison of distributions across features. On the other hand, scatter plots provide detailed and data-rich visualization that is best suited to analyze results of the clustering and dimensionality reduction. The circular plot should be used to examine correlations and associations between features and molecules. Taking comparison of molecules as an example, the circular plots use nodes to represent molecules and edges to denote correlations/associations between the molecules. The associations can be quantified with, for instance, Pearson's correlation coefficients (PCCs), in which case presence of an edge would mean that two molecules are correlated as a certain minimal value of PCC. Notably, the plots are interactive. We used the JavaScript package ECharts (v. 5.1.1) to implement plots in the web server version, and the matplotlib library (v. 3.4.2) (77) for the GUI version. Moreover, powered by two JavaScript libraries, NGL viewer v1.0.0 and Ketcher v2.1.0, the *iFeatureOmega* server can be used to display interactive three-dimensional protein and chemical structures, respectively.

### Webserver-construction of iFeatureOmega

*iFeatureOmega* resides on the Nectar (The National eResearch Collaboration Tools and Resources) cloud computing infrastructure, managed by the eResearch Centre at Monash University. The *iFeatureOmega* web server was built in a ‘Linux + Apache + Django’ framework and is equipped with 16 cores, 64 GB memory and 2 TB hard disk space. The server supports five popular web browsers including the Internet Explorer (≥v.7.0), Microsoft Edge, Mozilla Firefox, Google Chrome and Safari.

### iFeatureOmega implementation and visualization

The CLI- and GUI-based versions of *iFeatureOmega* were implemented and visualized using Python (v3.7.4), together with the third-party software packages including Biopython (v.1.78), Pandas (v.1.1.3) (78), Numpy (v.1.19.2) (79), NetworkX (v.2.5) (80), RDKit (v. 2020.03.3.0) (18), Matplotlib (v.3.1.2) (77), DSSP (v.3.0.0) (70,81) and MSMS (v.2.6.1) (73). The latter version of *iFeatureOmega* was implemented by PyQt5 (v.5.9.2).

## RESULTS

### Calculation and analysis of feature sets using iFeatureOmega

**Figure 1.**
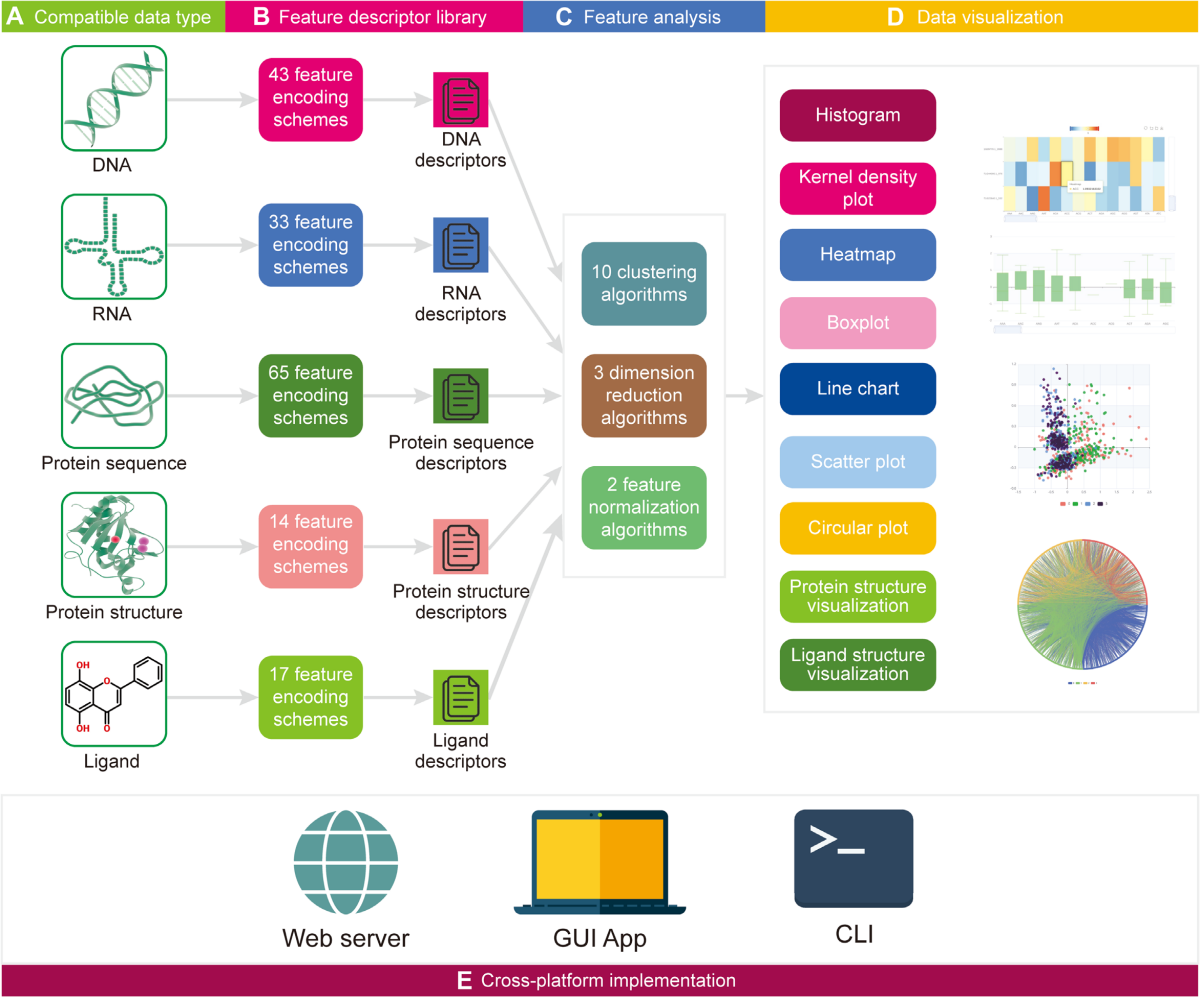
The *iFeatureOmega* architecture with three version applications, including *iFeatureOmega-Web*, *iFeatureOmega-GUI* and *iFeatureOmega-CLI*.

The core of *iFeatureOmega* platform is a python package implemented using PyQt5 in the GUI version, Apache ECharts for the data visualization in the webserver, RDKit (18) for the ligand descriptor calculation, and scikit-learn (85) for the feature analysis. We implemented code for the feature extraction for the DNA, RNA and protein sequences and protein structure. The *iFeatureOmega*’s architecture is summarized in Figure 1.

The ***web server*** version (http://ifeatureomega.erc.monash.edu/) performs feature extraction, calculation, analysis and visualization on the server-side, relieving users from the necessity to utilize local computational resources (Supplementary Figure S1a). The server includes five webpages that are accessible via the navigation bar at the top of the main page. To calculate features for protein, DNA and/or RNA sequences, users are required to use the FASTA format and either upload a file containing the sequences or copy them into the provided entry text box. To calculate protein structural features, users should provide input protein structure(s) in the PDB (86) (https://www.rcsb.org/) format (i.e. PDB or CIF format) or a PDB accession. For the analysis of ligands, uses should use SMILES encoding or files in the SDF format. Moreover, ligand molecules be also drawn using Ketcher (https://github.com/epam/ketcher), which is a web-based interface for editing chemical structures (Supplementary Figure S1b). The resulting software-drawn ligand structure can be converted to SMILES format and displayed.

The features and results of feature analysis are displayed on the ‘Result’ page. Multiple tabs are available to view the results. The calculated features are available for download in four formats including SVM (https://www.cs.cornell.edu/people/tj/svm_light/), Comma-Separated Values (CSV), Tab Separated Values (TSV), and Waikato Environment for knowledge Analysis (WEKA) (87). *iFeatureOmega* generates nine types of interactive plots including histograms, kernel density plots, heatmaps, boxplots, line charts, scatters, circular plots, three-dimensional protein structures and ligand structures. For example, a scatter plot is useful to summarize results of feature clustering, where each point represents an individual molecule, and different colors represent distinct categories of clusters. Relevant information, including sample name and category, are displayed in a table when points are selected using the lasso tool.

**Figure 2.**
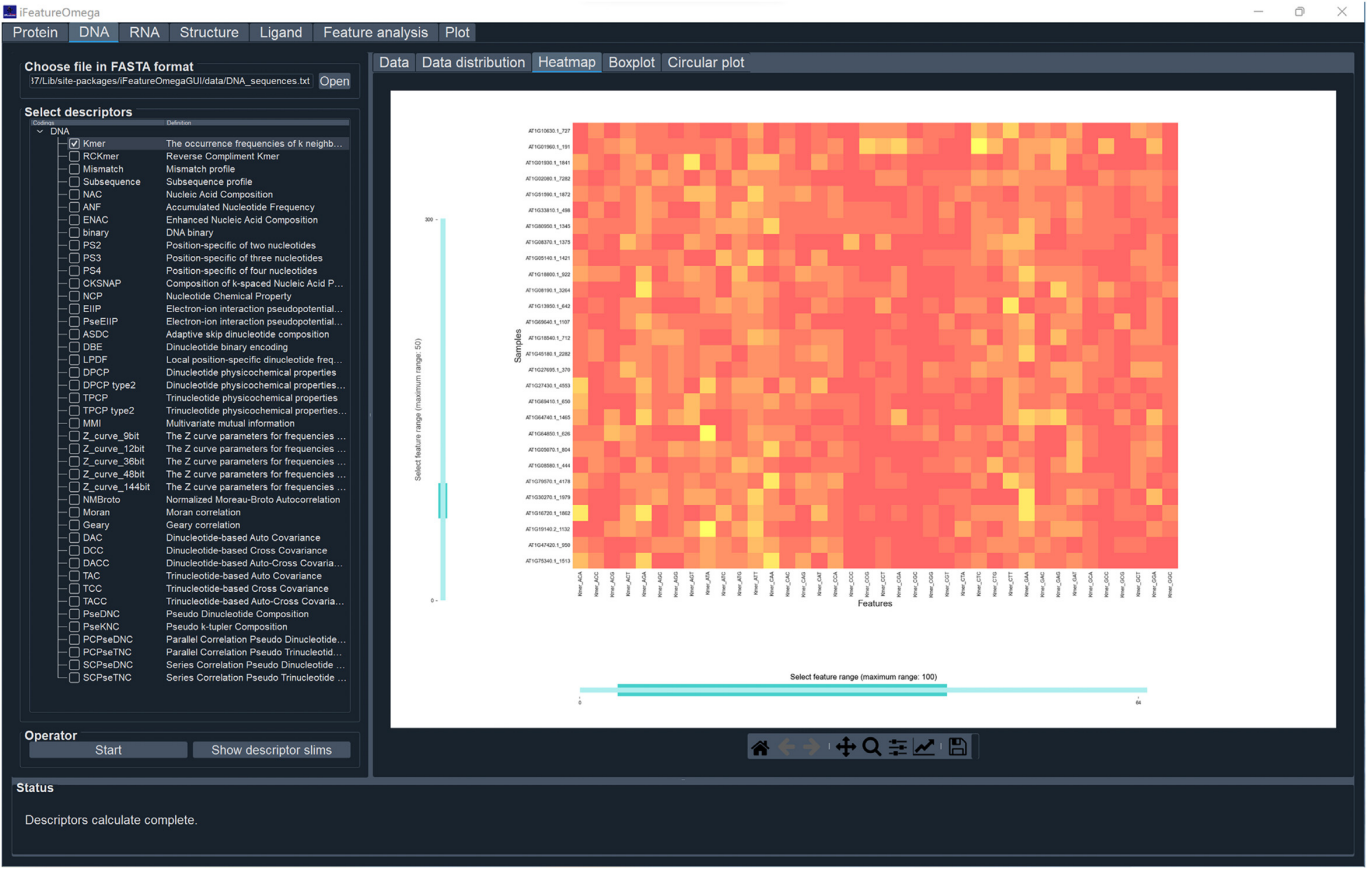
The screenshot showing the GUI version of *iFeatureOmega*, including ‘Protein’ panel, ‘DNA’ panel, ‘RNA’ panel, ‘Structure’ panel, ‘Ligand’ panel, ‘Feature analysis’ panel and ‘Plot’ panel.

*The* ***stand-alone*** versions, including GUI and CLI interfaces, provide users with the ability to run *iFeatureOmega* on their local hardware. This avoids the potential pitfalls of web servers that require uninterrupted availability of Internet and could be delayed by extensive use. Moreover, these versions offer the option of running feature calculation and analysis in batches, while the web server version provides descriptors for one feature type for each submitted task. For the GUI-based version (Figure 2), seven tab-widgets that implement different functionality are available. For example, using the ‘DNA’ tab, users can select to obtain one or more feature descriptors for DNA sequences. After clicking the ‘Start’ button, the selected descriptors are calculated, and relevant statistical plots are produced to facilitate analysis of the resulting features. The selected subset of feature sets is displayed in a convenient table widget, and includes molecule name, (column) and associated values. The plots are displayed in a single tab widget and can be conveniently saved in a variety of image formats, such as PNG, JPG, PDF and TIFF. Moreover, most of the plot types, except only for the histogram and kernel density plot, are interactive. For instance, in the heatmaps, users can specify which data to display by adjusting the range sliders on rows (i.e. samples) and/or columns (i.e. descriptors). A detailed description of the interactive plots is available in the online manual.

The CLI-based version, which was developed and implemented as a python package, targets experienced bioinformaticians and programmers. This version accepts a JSON format configuration file, allowing users to conveniently specify parameter values that define which features and algorithms for feature analysis should be run (Supplementary Figure S2a). There are seven major schemes for this version of *iFeatureOmega* including ‘iProtein’, ‘iDNA’, ‘iRNA’, ‘iStructure’ and ‘iLigand’, which implement methods for the extraction of features from protein sequences, DNA sequences, RNA sequences, protein structures and ligand molecules, respectively. The iAnalysis scheme provides access to the feature analysis algorithms while the iPlot scheme should be used to produce plots. Supplementary Figure S2b shows the source code pertaining to feature extraction and feature analysis in the CLI-based version. The online manual provides further details concerning the use of the web server, CLI and GUI versions.

### Demonstrating the utility and versality of iFeatureOmega

We present three diverse practical applications of *iFeatureOmega* to demonstrate versatility and usefulness of this platform. Each application utilizes a different *iFeatureOmega* interface and concerns a different type of inputs. They include (i) a representation of the zinc-binding sites microenvironment in protein structures, (ii) visualization of the feature descriptors for long noncoding RNAs (lncRNAs) and (iii) extraction and visualization of features generated for the adenosine A_2A_ receptors.

**Figure 3.**
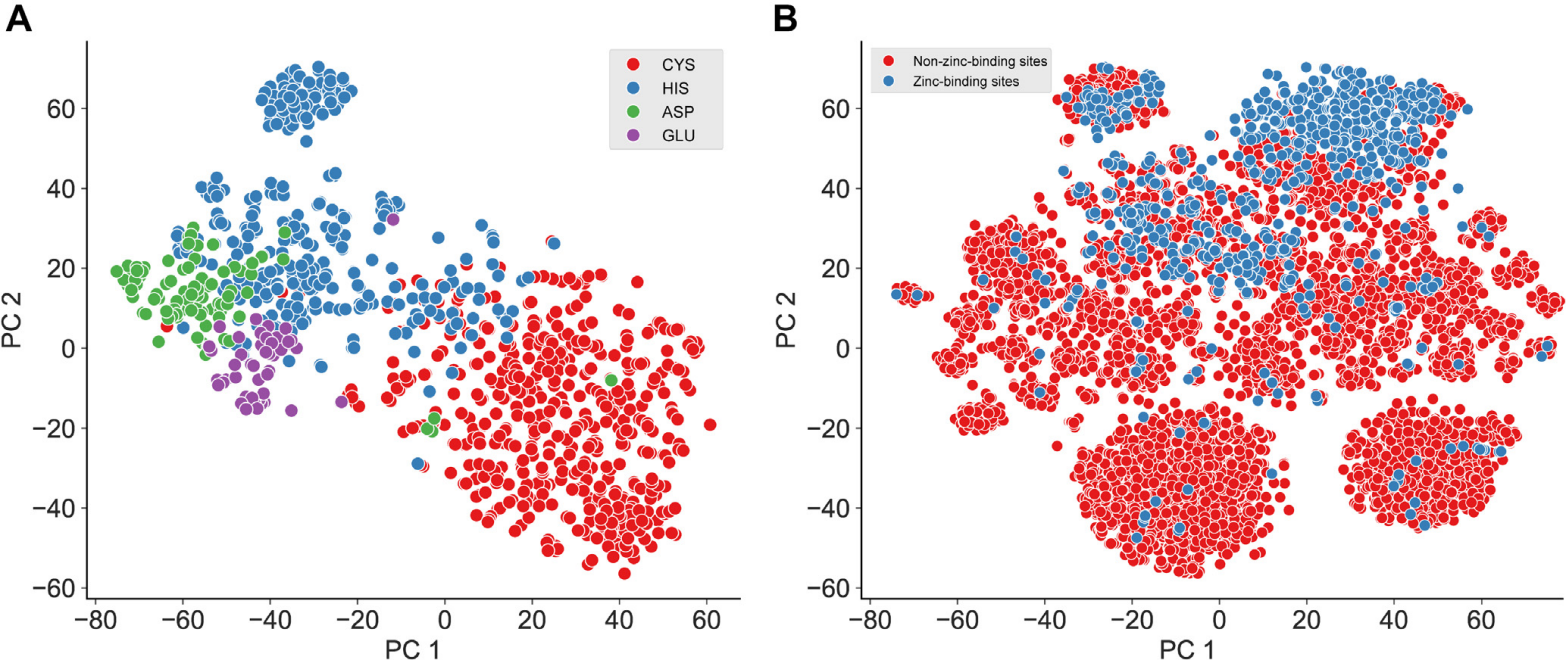
The feature analysis result for protein zinc-binding sites using the ‘AAC_type2’ feature extraction method and the local CLI version of *iFeatureOmega*, including the data visualization for four types of zinc-binding sites (**A**), the data visualization for zinc-binding sites and non-zinc-binding sites (**B**).

Zinc is one of the most important and ubiquitous trace elements in microorganisms, plants and animals. Similar to other types of metal ions, zinc is involved in the catalysis of some enzymes (e.g. cytidine deaminase (88) and 6-pyruvoyl tetrahydropterin synthase (89)) and plays key roles in governing some protein structures, such as the zinc finger proteins (90). Zinc-binding sites contain four main types of amino acids—CYS, HIS, GLU and ASP—‘CHED’ for short (90). Here, we applied the CLI-based version of *iFeatureOmega* to depict the three-dimensional microenvironment (i.e. amino acids content) around the zinc-binding sites using a published dataset (82). This dataset contains 999 protein-zinc binding sites (531 CYS, 325 HIS, 92 ASP and 51 GLU) and 7426 non-zinc-binding sites in 208 non-redundant PDB chains. The structural feature sets ‘AAC_type2’ were used to obtain features. For the feature set, each zinc-binding CHED and non-zinc-binding CHED residue was specified by their three-dimensional position and the radius defining their ‘neighborhood’, and shells were formed around each target site (Supplementary Figure S3). Then, the frequency of each amino acid type was calculated for cumulative shells. The PDB structures were downloaded, and a python script written to execute the extraction of features. First, we applied the *t*-SNE algorithm to display the distribution of the 999 zinc-binding sites (Figure 3A). According to the dimensionality reduction result, the zinc-binding sites of the same residues clustered well (i.e. the zinc-binding sites with the same residue cluster together), indicating that ‘AAC_type2’ features are suitable to capture the three-dimensional microenvironmental characteristics around the zinc-binding sites. Second, we performed a linear discriminant analysis (LDA) analysis to display the difference in distribution between zinc-binding sites and non-zinc-binding sites (Figure 3B). The results revealed a marked difference in the feature values between the zinc-binding and non-zinc-binding sites.

**Figure 4.**
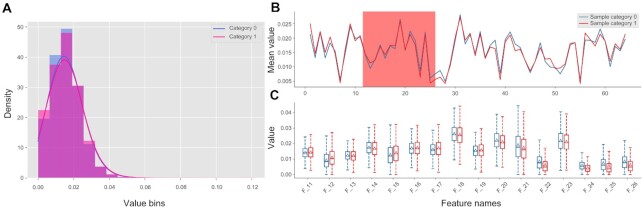
The data visualization for lncRNA sequences and mRNA sequences using local GUI version of *iFeatureOmega*, including the histogram and kernel density plot shows the distribution difference between lncRNA and mRNA sequences (**A**), line chart shows the mean value difference (**B**) and box plot shows the distribution difference (**C**) for each descriptor between lncRNA and mRNA sequences.

The lncRNAs are transcripts of >200 bp in length that do not code for proteins (83). Various algorithms have been proposed to predict lncRNAs and their functions from mRNAs (91). The ‘Kmer’ based features were shown to be effective in the prediction of lncRNAs (5,92,93). Here, we utilized the GUI-based version of *iFeatureOmega* to compare distributions using the ‘Kmer’ features between lncRNAs and mRNAs using a recent data set of 4200 lncRNA and 4200 mRNA sequences from mouse (*Mus musculus*) (83). First, we used the ‘DNA’ panel to obtain the Kmer features for both sets of sequences. The features for lncRNAs were taken as positive samples and labelled as ‘1’; while the features generated from the mRNA sequences were taken as negative samples and labeled as ‘0’. Second, we applied the ‘iPlot’ panel to produce several plots including a histogram, a kernel density plot, a line chart and a boxplot (Figure 4). The histogram and kernel density plot (Figure 4A) display statistical distributions of the two sets of features. The line chart (Figure 4B; display area is adjustable) shows the mean difference between the lncRNAs and mRNAs data for each descriptor. Finally, the boxplot (Figure 4C) illustrates the difference in the distribution of feature values between lncRNAs and mRNAs. This demonstrates how easy it is to utilize *iFeatureOmega* to encode the features and obtain insightful and comprehensive analysis of their values.

**Figure 5.**
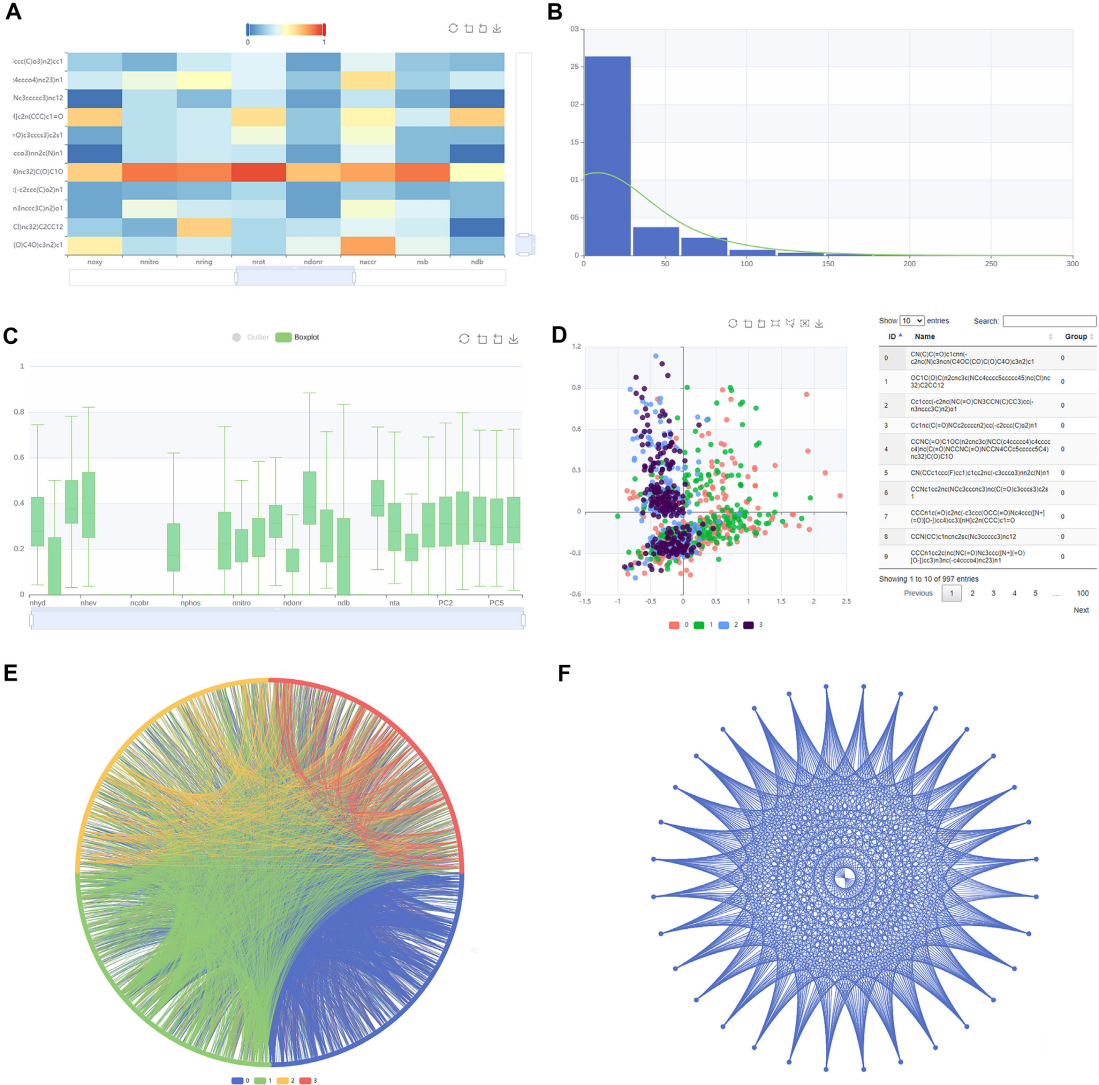
The data visualization for ligands with constitution (A–D) and geary (E and F) features. The feature matrix is shown as heatmap, and the value in the matrix can be filtered with the color bar (**A**). The distribution of whole feature values is shown in a histogram, and the line plot represents the probability density curve fitted with kernel density estimation (**B**). The distribution of each feature is shown using the box plots (**C**). The first two component of PCA on these calculated features with provided labels are shown using a scatter plot, and the detailed information are listed in the table at the right side when the points are railed out (**D**). The similarity of these molecules is exhibited as relationship plot in which each node stands for a molecule (**E**). If it is similar to another molecule, there will be an edge between them, and the similarity value will be shown when the mouse pointer hovers. The different clusters obtained by the clustering algorithms will be labeled with different colors. The relationship plot can also visualize the similarity of features with different distance metrics (**F**).

The adenosine A_2A_ receptor is one of the most extensively studied G protein-coupled receptors in human (84), and has been reported as a promising target for drugs against Parkinson's, cardiovascular and inflammatory diseases (94). Here, we applied the web server version of *iFeatureOmega* to extract, calculate and visualize features for the A_2A_ receptors. We selected 997 small organic molecules as active ligands for the A_2A_ receptors based on recent work (84). This set was divided into four different chemotypes: one representing known ligands obtained from ChEMBL database (3) (cluster 0) and the other three inferred by three different deep-learning (DL)-based molecular generators (clusters 1, 2 and 3). After these molecules were uploaded into the web server, we selected and calculated the ‘constitution’ and ‘geary’ features and visualized them using several plots (Figure 5). Relations between these features are shown with the help of an interactive heatmap (Figure 5A), in which feature values can be filtered via the color bar. The distributions of the feature values are visualized in the histogram and we fitted them into a probability density curve via kernel density estimation (Figure 5B). Next, we produced a boxplot to analyze distributions of values of individual features (Figure 5C). We also used the scatter plot (Figure 5D) to study two main components generated via principal components analysis (PCA) of the calculated features, where the four different chemotypes (clusters) are conveniently color-coded. The latter plot indicates that clusters 0 and 1 and clusters 2 and 3 overlap, suggesting that their corresponding features can be used to accurately separate clusters 0 and 1 from clusters 2 and 3. The circular plot (Figure 5e) visualizes similarity between these molecules where each node on the rim represents a molecule and edges are used to denote that the two molecules are similar. The similarity scores associated with the edges are shown when the mouse hovers over them. We produced the second circular plot (Figure 5F) to visualize the calculated features with different distance metrics shown/given.

## CONCLUSIONS

The development of modern feature-engineering, analysis and visualization tools for the characterization and classification of protein, nucleic acid and ligand molecules plays critically important role for machine-learning and deep-learning based exploration of genomic and proteomic data sets. However, our analysis suggests that there are no ‘one-stop’ computational toolkits for this purpose. Therefore, using our prior iFeature (27) tool as a foundation, we constructed a complete and convenient *iFeatureOmega* platform for the extraction and analysis of features from molecules and molecular data sets. Our platform includes three interface versions to satisfy the needs of a wide spectrum of users, including biologists and biochemists with limited bioinformatics expertise who would benefit from the easy-to-use web server or stand-alone GUI versions, and experienced programmers and bioinformaticians who may prefer to use the CLI interface. Moreover, *iFeatureOmega* supports processing DNA, RNA, protein and ligand data, integrates many feature sets, and uses a broad array of algorithms to analyze and display the resulting features and statistical information. Nearly all plots and graphics that are included in *iFeatureOmega* are interactive, allowing users to conveniently select and filter relevant data and setup the plot area.

The *iFeatureOmega* web server can be found at http://ifeatureomega.erc.monash.edu. The standalone versions can be obtained from https://github.com/Superzchen/iFeatureOmega-GUI/ and https://github.com/Superzchen/iFeatureOmega-CLI/; we distribute them under the Massachusetts Institute of Technology (MIT) licence. Given the significant uptake of our much more limited iFeature platform, we believe that *iFeatureOmega* will enjoy even more wide-spread use as an effective and accessible tool for the extraction and analysis features from molecular data sets. Our platform can be applied to molecular data across different organisms (microbes, plants and animals) and scales (protein families, cell, tissues, whole genomes).

## DATA AVAILABILITY

Three data sets used to demonstrate the utility of *iFeatureOmega* are publicly accessible. Specifically, the protein-zinc binding data set was produced by Passerini *et al.* (82) and is accessible at https://github.com/Superzchen/iFeatureOmega-GUI/blob/main/data/Passerni_dataset.zip; the lncRNA and mRNA sequence datasets were produced by Han *et al.* (83) and is accessible at https://github.com/HAN-Siyu/LncFinder/blob/master/Data/Datasets/Mouse.zip; the adenosine A_2A_ receptor dataset were produced by Liu *et al.* (84) and is accessible at https://github.com/Superzchen/iFeatureOmega-GUI/blob/main/data/A2A_datasets.zip.

## CODE AVAILABILITY

The *iFeatureOmega* web server is freely accessible via http://ifeatureomega.erc.monash.edu, and the stand-alone versions of the platform can be downloaded at https://github.com/Superzchen/iFeatureOmega-GUI/ and https://github.com/Superzchen/iFeatureOmega-CLI/ under the MIT License.

## Supplementary Material

gkac351_Supplemental_Files
