# Supplementary material for: iFeatureOmega: an integrative platform for engineering, visualization and analysis of features from molecular sequences, structural and ligand data sets
Source: Nucleic Acids Res. 2022 May 7;50(W1):W434–47. doi: 10.1093/nar/gkac351 (PMC9252729; doi:10.1093/nar/gkac351)
Supplement: gkac351_Supplemental_Files [file gkac351_supplemental_files.zip › iFeatureOmega_supplementary file_FINAL.docx]

***iFeatureOmega –* an integrative platform for engineering, visualization and analysis of features from molecular sequences, structural and ligand data sets**

Zhen Chen^1,2,†^, Xuhan Liu^3,†^, Pei Zhao^4,†^, Chen Li^5,†^, Yanan Wang^5^, Fuyi Li^5^, Tatsuya Akutsu^6^, Chris Bain^7^, Robin B. Gasser^8^, Junzhou Li^1^, Zuoren Yang^4,*^_,_ Xin Gao^9,*^, Lukasz Kurgan^10,*^, and Jiangning Song^5,7,*^

^1^Collaborative Innovation Center of Henan Grain Crops, Henan Agricultural University, Zhengzhou 450046, China, ^2^Center for Crop Genome Engineering, Henan Agricultural University, Zhengzhou 450046, China, ^3^Drug Discovery and Safety, Leiden Academic Centre for Drug Research, Einsteinweg 55, Leiden, 2333 CC, The Netherlands, ^4^State Key Laboratory of Cotton Biology, Institute of Cotton Research of Chinese Academy of Agricultural Sciences (CAAS), Anyang, 455000, China, ^5^Monash Biomedicine Discovery Institute and Department of Biochemistry and Molecular Biology, Monash University, Melbourne, Victoria 3800, Australia, ^6^Bioinformatics Center, Institute for Chemical Research, Kyoto University, Kyoto 611-0011, Japan ^7^Monash Data Future Institutes, Monash University, Melbourne, Victoria 3800, Australia, ^8^Department of Veterinary Biosciences, Melbourne Veterinary School, The University of Melbourne, Parkville, Victoria 3010, Australia, ^9^Computational Bioscience Research Center (CBRC), Computer, Electrical and Mathematical Sciences and Engineering Division, King Abdullah University of Science and Technology (KAUST), Thuwal 23955, Saudi Arabia, ^10^Department of Computer Science, Virginia Commonwealth University, Richmond, VA, USA

^†^These authors contributed equally to this work.

^*^To whom the correspondence should be addressed.

Jiangning Song: Tel: +61-3-9902-9304; Email: [Jiangning.Song@monash.edu](mailto:Jiangning.Song@monash.edu), or Lukasz Kurgan: Tel: +1-804-827-3986; Email: [lkurgan@vcu.edu](mailto:lkurgan@vcu.edu), or Zuoren Yang: Tel: +86-371-5591-2760; Email: [yangzuoren@caas.cn](mailto:yangzuoren@caas.cn), or Xin Gao: Tel: +966-128080323; E-mail: [xin.gao@kaust.edu.sa](mailto:xin.gao@kaust.edu.sa).

**Supplementary Figures**


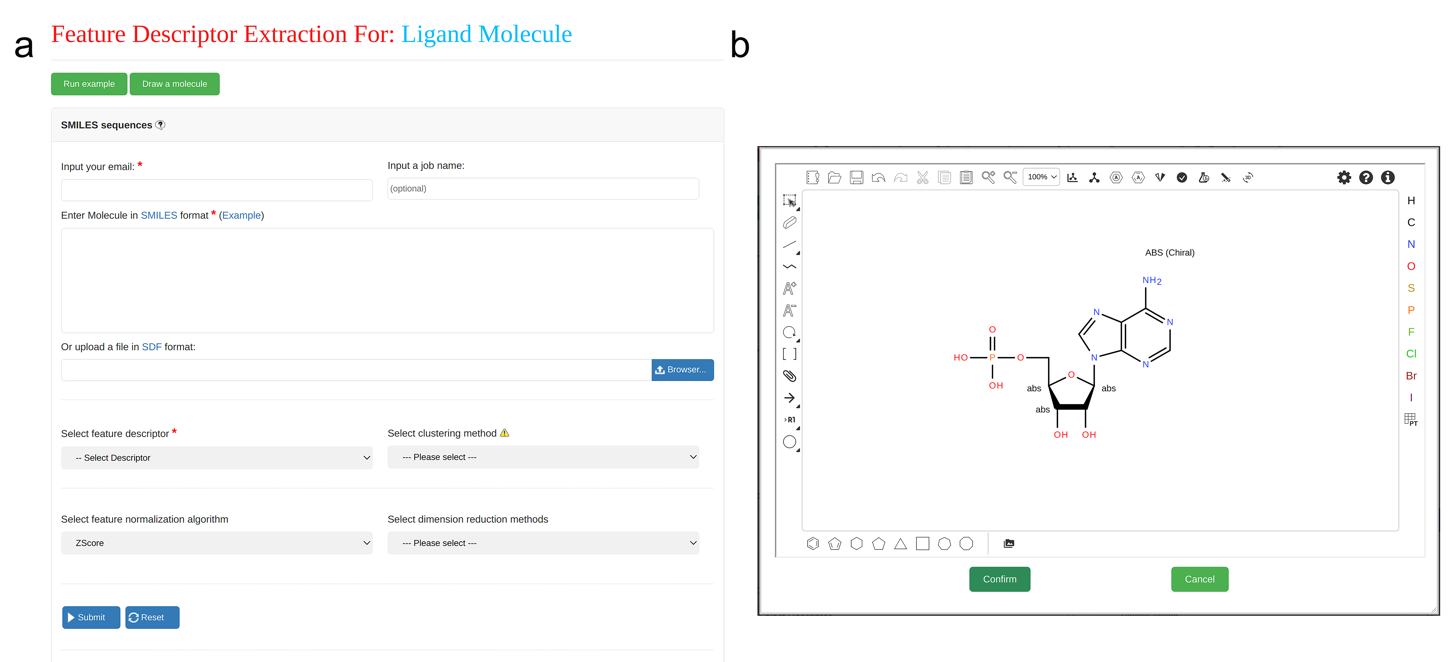


**Figure S1.** A screenshot of the webpage for data uploading for ligands. The datatype of ligands can be either SMILES submitted through text area, or ‘.sdf’ format document uploaded through the file upload component (a). Users can also draw the structure of these ligands one by one with ‘Ketcher’, which is a web-based interface of chemical structure editor integrated in our webserver (b).


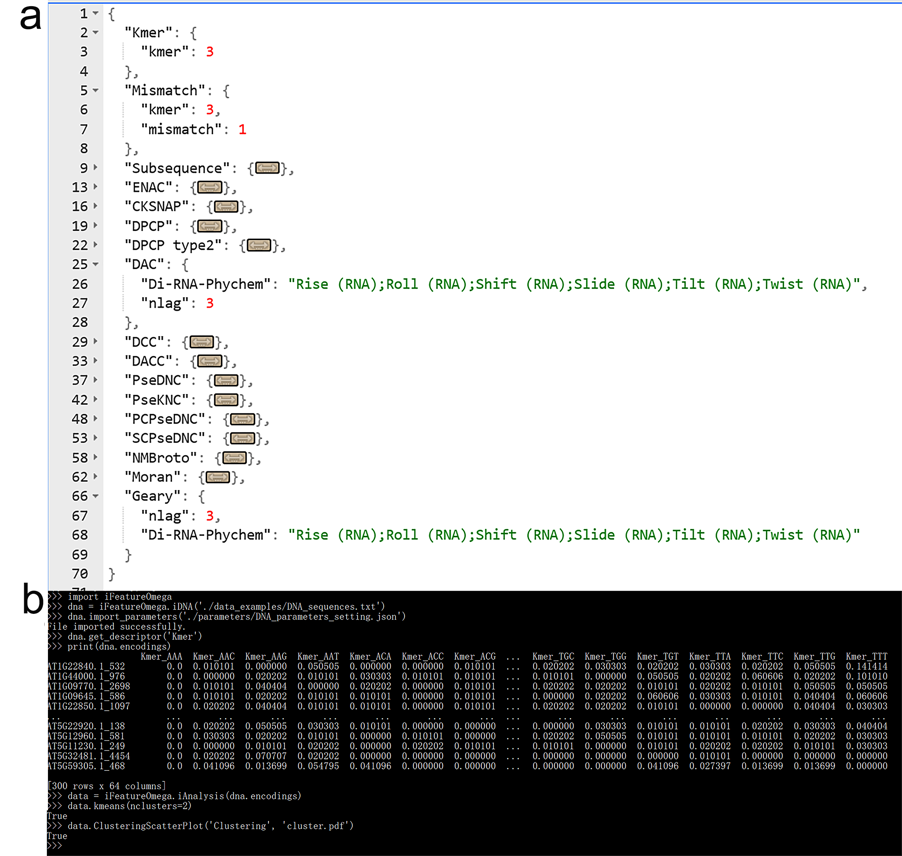


**Figure S2.** The screenshot showing the CLI version of *iFeatureOmega* application. (a) a snapshot of the configuration file that specifies the parameters for DNA descriptors generation and (b) the code on how to extract features and perform feature analysis by using the command line version.


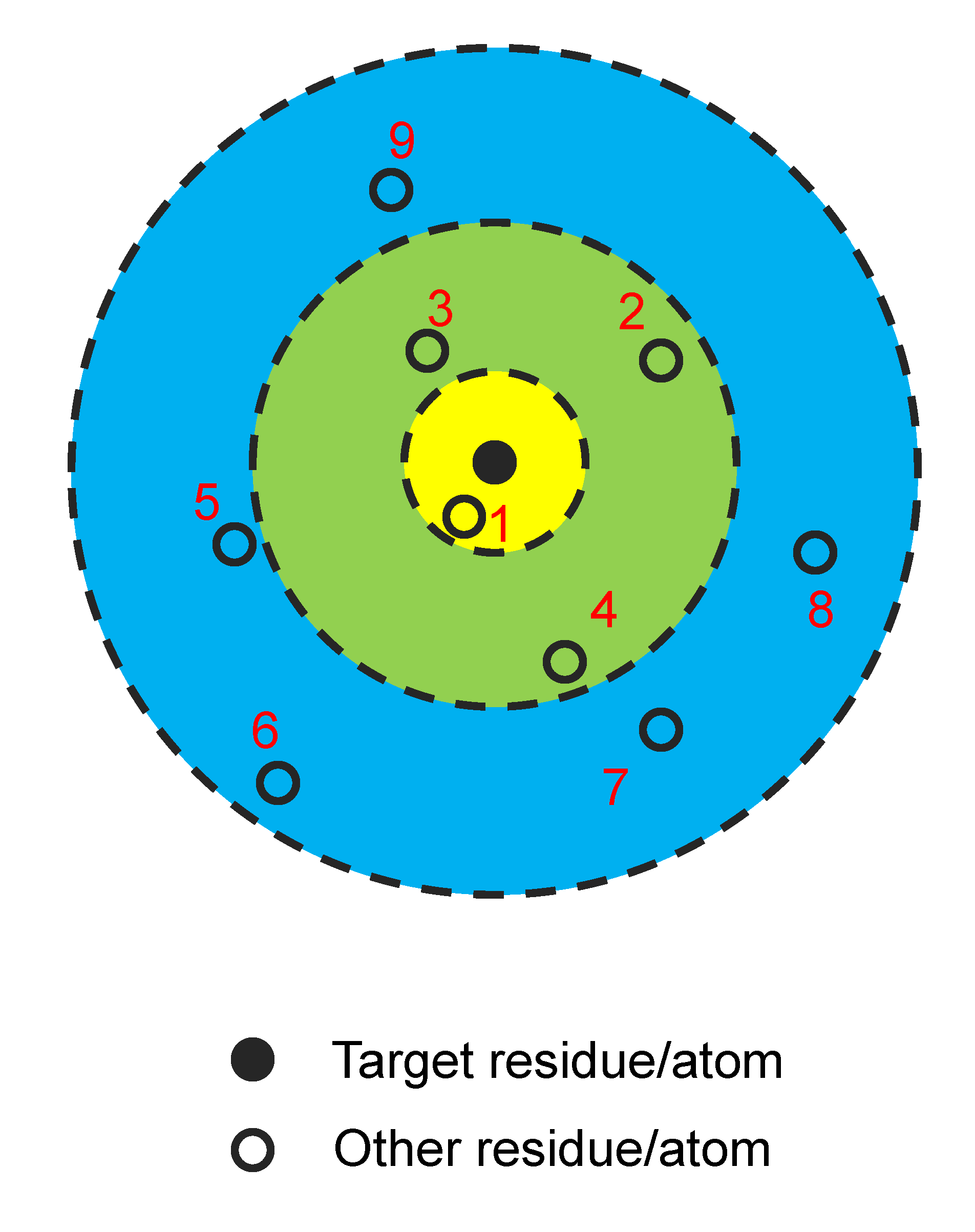


**Figure S3**. **An example of feature extraction for protein structure “AAC_type1/2” descriptors**. The solid dot represents the target site, while the cycles denote other residues. The microenvironment of the target site is specified as a three-dimensional position and a radius defining the neighborhood. Shells are formed around the site (the yellow part is the first shell, the green part is from the second shell, while the blue part is from the third shell). For AAC_type1 feature descriptor, the frequency of amino acids in the first shell (i.e., residue #1), the second shell (i.e., residue #2, #3 and #4) and the third shell (i.e., residue #5-9) will be calculated, respectively. For AAC_type2 descriptor, at first the frequency of amino acids in the first shell (i.e., residue #1) is calculated. Then, the frequency of amino acids in the first and second shells (i.e., residue #1-4) will be calculated. Finally, the frequency of amino acids in all the three shells (i.e., residue #1-9) will be calculated.

**Supplementary Tables**

**Supplementary Table 1.** Feature descriptors calculated by *iFeatureOmega* for protein sequences.

| **Feature category** | **Feature sets (Abbreviation)** | **Reference** |
| --- | --- | --- |
| Amino acid composition | Amino acid composition (AAC) | (1) |
|  | Enhanced amino acid composition (EAAC) | (2,3) |
|  | Composition of *k*-spaced amino acid pairs (CKSAAP type 1 and type 2) | (4,5) |
|  | Kmer (dipeptides and tripeptides) composition (DPC and TPC type 1 and type 2) | (1,6) |
|  | Dipeptide deviation from expected mean (DDE) | (6) |
|  | Composition (CTDC) | (7-11) |
|  | Transition (CTDT) | (7-11) |
|  | Distribution (CTDD) | (7-11) |
|  | Conjoint triad (CTriad) | (12) |
|  | Conjoint *k*-spaced Triad (KSCTriad) | (2,3) |
|  | Adaptive skip dipeptide composition (ASDC) | (13) |
|  | PseAAC of distance-pairs and reduced alphabet (DistancePair) | (14,15) |
| Grouped amino acid composition | Grouped amino acid composition (GAAC) | (2,3) |
|  | Grouped enhanced amino acid composition (GEAAC) | (2,3) |
|  | Composition of *k*-spaced amino acid group pairs (CKSAAGP type 1 and type 2) | (2,3) |
|  | Grouped dipeptide composition (GDPC type 1 and type 2) | (2,3) |
|  | Grouped tripeptide composition (GTPC type 1 and type 2) | (2,3) |
| Autocorrelation | Moran (Moran) | (16,17) |
|  | Geary (Geary) | (18) |
|  | Normalized Moreau-Broto (NMBroto) | (19) |
|  | Auto covariance (AC) | (20-22) |
|  | Cross covariance (CC) | (20-22) |
|  | Auto-cross covariance (ACC) | (20-22) |
| Quasi-sequence-order | Sequence-order-coupling number (SOCNumber) | (23-25) |
|  | Quasi-sequence-order descriptors (QSOrder) | (23-25) |
| Pseudo-amino acid composition | Pseudo-amino acid composition (PAAC) | (26,27) |
|  | Amphiphilic PAAC (APAAC) | (26,27) |
|  | Pseudo *K*-tuple reduced amino acids composition (PseKRAAC_type 1 to type 16) | (28) |
| Residue representation | Binary - 20bit (binary) | (29,30) |
|  | Binary - 6bit (binary_6bit) | (15,31) |
|  | Binary - 5bit (binary_5bit_type 1 and type 2) | (15,32) |
|  | Binary - 3bit (binary_3bit_type 1 to type 7) | (13) |
|  | Learn from alignments (AESNN3) | (15,33) |
|  | Overlapping property features - 10 bit (OPF_10bit) | (13) |
|  | Overlapping property features - 7 bit (OPF_7bit type 1 to type 3) | (13) |
| Physicochemical property | AAindex (AAindex) | (34) |
| BLOSUM matrix | BLOSUM62 (BLOSUM62) | (35) |
| Z-Scales index | Z-Scales (Zscale) | (36) |
| Similarity-based descriptor | *K*-nearest neighbor (KNN) | (37) |

**Supplementary Table 2.** Feature descriptors calculated by *iFeatureOmega* for DNA and RNA sequences.

| **Feature category** | **Feature sets (Abbreviation)** | **Sequence type** | **Reference** |
| --- | --- | --- | --- |
| Nucleic acid composition | Nucleic acid composition (NAC) | DNA/RNA | (3) |
|  | Enhanced nucleic acid composition (ENAC) | DNA/RNA | (3) |
|  | *k*-spaced nucleic acid pairs (CKSNAP type 1 and type 2) | DNA/RNA | (3) |
|  | Basic kmer (Kmer type 1 and type 2) | DNA/RNA | (38) |
|  | Reverse compliment kmer (RCKmer type 1 and type 2) | DNA | (39,40) |
|  | Accumulated nucleotide frequency (ANF) | DNA/RNA | (41) |
|  | Nucleotide chemical property (NCP) | DNA/RNA | (41) |
|  | The occurrence of kmers, allowing at most m mismatches (Mismatch) | DNA/RNA | (15) |
|  | The occurrences of kmers, allowing non-contiguous matches (Subsequence) | DNA/RNA | (15) |
|  | Adaptive skip dinucleotide composition (ASDC) | DNA/RNA | (13) |
|  | Local position-specific dinucleotide frequency (LPDF) | DNA/RNA | (42) |
|  | The Z curve parameters for frequencies of phase-specific mononucleotides (Z_curve_9bit) | DNA/RNA | (43) |
|  | The Z curve parameters for frequencies of phase-independent dinucleotides (Z_curve_12bit) | DNA/RNA | (43) |
|  | The Z curve parameters for frequencies of phase-specific dinucleotides (Z_curve_36bit) | DNA/RNA | (43) |
|  | The Z curve parameters for frequencies of phase-independent trinucleotides (Z_curve_48bit) | DNA/RNA | (43) |
|  | The Z curve parameters for frequencies of phase-specific trinucleotides (Z_curve_144bit) | DNA/RNA | (43) |
| Position-specific of n-nucleotides | Binary (binary) | DNA/RNA | (29,30) |
|  | Dinucleotide binary encoding (DBE) | DNA/RNA | (42) |
|  | Position-specific of two nucleotides (PS2) | DNA/RNA | (15,44) |
|  | Position-specific of three nucleotides (PS3) | DNA/RNA | (15,44) |
|  | Position-specific of four nucleotides (PS4) | DNA/RNA | (15,44) |
|  | Position-specific trinucleotide propensity based on single-strand (PSTNPss) | DNA/RNA | (45,46) |
|  | Position-specific trinucleotide propensity based on double-strand (PSTNPds) | DNA | (45,46) |
| Electron-ion interaction pseudopotentials | Electron-ion interaction pseudopotentials value (EIIP) | DNA | (47,48) |
|  | Electron-ion interaction pseudopotentials of trinucleotide (PseEIIP) | DNA | (47,48) |
| Autocorrelation and cross-covariance | Dinucleotide-based auto covariance (DAC) | DNA/RNA | (20-22) |
|  | Dinucleotide-based cross covariance (DCC) | DNA/RNA | (20-22) |
|  | Dinucleotide-based auto-cross covariance (DACC) | DNA/RNA | (20-22) |
|  | Trinucleotide-based auto covariance (TAC) | DNA | (20) |
|  | Trinucleotide-based cross covariance (TCC) | DNA | (20) |
|  | Trinucleotide-based auto-cross covariance (TACC) | DNA | (20) |
|  | Moran (Moran) | DNA/RNA | (16,17) |
|  | Geary (Geary) | DNA/RNA | (18) |
|  | Normalized Moreau-Broto (NMBroto) | DNA/RNA | (19) |
| Physicochemical property | Dinucleotide physicochemical properties (DPCP type 1 and type 2) | DNA/RNA | (49) |
|  | Trinucleotide physicochemical properties (TPCP type 1 and type 2) | DNA | (49) |
| Mutual information | Multivariate mutual information (MMI) | DNA/RNA | (50) |
| Pseudo nucleic acid composition | Pseudo dinucleotide composition (PseDNC) | DNA/RNA | (20,51) |
|  | Pseudo *k*-tupler composition (PseKNC) | DNA/RNA | (20,51) |
|  | Parallel correlation pseudo dinucleotide composition (PCPseDNC) | DNA/RNA | (20,51) |
|  | Parallel correlation pseudo trinucleotide composition (PCPseTNC) | DNA | (20,51) |
|  | Series correlation pseudo dinucleotide composition (SCPseDNC) | DNA/RNA | (20,51) |
|  | Series correlation pseudo trinucleotide composition (SCPseTNC) | DNA | (20,51) |
| Similarity-based descriptor | *K*-nearest neighbor (KNN) | DNA/RNA | (50) |

**Supplementary Table 3.** Feature descriptors calculated by *iFeatureOmega* for ligands.

| **Feature category** | **Feature sets** | **Reference** |
| --- | --- | --- |
| Constitution | Molecular constitutional descriptors | (52,53) |
| Topology | Topological descriptors | (52,53) |
|  | Molecular connectivity indices | (52,53) |
| E-State | E‑state descriptors | (52,53) |
| Bask | Bask descriptors | (52,53) |
| Burden | Burden descriptors | (52,53) |
| Kappa | Kappa shape descriptors | (52,53) |
| Autocorrelation | Moreau–Broto autocorrelation | (52,53) |
|  | Moran autocorrelation | (52,53) |
|  | Geary autocorrelation | (52,53) |
| Charge | Charge descriptors | (52,53) |
| Property | Molecular property | (52,53) |
| Pharmacophore | Potential pharmacophore point descriptors | (52,53) |
| MOE-type | MOE‑type descriptors | (52,53) |
| Fingerprints | MACCS fingerprints | (52,53) |
|  | Morgan fingerprints | (52,53) |
|  | E-state fingerprints | (52,53) |

**Supplementary Table 4.** Feature descriptors calculated by *iFeatureOmega* for protein structures.

| **Feature category** | **Feature sets (Abbreviation)** |
| --- | --- |
| Amino acids composition | Amino acids content type 1 (AAC_type1) |
|  | Amino acids content type 2 (AAC_type2) |
| Grouped amino acids composition | Grouped amino acids content type 1 (GAAC_type1) |
|  | Grouped amino acids content type 2 (GAAC_type2) |
| Secondary structure | Secondary structure elements (3) type 1 (SS3_type1) |
|  | Secondary structure elements (3) type 2 (SS3_type2) |
|  | Secondary structure elements (8) type 1 (SS8_type1) |
|  | Secondary structure elements (8) type 2 (SS8_type2) |
| Half sphere exposure | Half sphere exposure α (HSE_CA) |
|  | Half sphere exposure β (HSE_CB) |
| Residue depth | Residue depth (Residue depth) |
| Atom composition | Atom content type 1 (AC_type1) |
|  | Atom content type 2 (AC_type2) |
| Network-based index | Network-based index |

**Supplementary Table 5.** The feature analysis approaches provided in *iFeatureOmega*.

| **Method** | **Algorithm (Abbreviation)** | **Reference** |
| --- | --- | --- |
| Clustering | *k*-means (kmeans) | (54,55) |
|  | Mini-Batch *K*-means (MiniBatchKMeans) | (54,55) |
|  | Gaussian mixture (GM) | (54,55) |
|  | Agglomerative (Agglomerative) | (56) |
|  | Spectral (Spectral) | (57) |
|  | Markov clustering (MCL) | (58) |
|  | Hierarchical clustering (hcluster) | (54,59) |
|  | Affinity propagation clustering (APC) | (60) |
|  | Mean shift (meanshift) | (61) |
|  | DBSCAN (dbscan) | (62) |
| Dimensionality reduction | Principal component analysis (PCA) | (63) |
|  | Latent dirichlet allocation (LDA) | (64) |
|  | *t*-distributed stochastic neighbor embedding (*t*_SNE) | (65) |
| Feature normalization | Z-Score (ZScore) | (3) |
|  | Min-Max (MinMax) | (3) |

**Supplementary Table 6.** Graphical display options in *iFeatureOmega*.

| **Category** | **Type** | **Purpose** |
| --- | --- | --- |
| Feature descriptor visualization | Histogram | Display data distribution |
|  | Kernel density plot | Display data distribution |
|  | Heatmap | Display data distribution |
|  | Boxplot | Display data distribution |
|  | Line chart | Display data distribution |
|  | Scatter plot | Display clustering and dimensionality reduction result |
|  | Circular plot | Display the correlation of samples or descriptors |
| Protein structure visualization |  | Display the 3-D protein structure |
| Ligand structure visualization |  | Display chemical structure |

**References**

1. Bhasin, M. and Raghava, G.P. (2004) Classification of nuclear receptors based on amino acid composition and dipeptide composition. *J Biol Chem*, **279**, 23262-23266.

2. Zhou, C., Wang, C., Liu, H., Zhou, Q., Liu, Q., Guo, Y., Peng, T., Song, J., Zhang, J., Chen, L. *et al.* (2018) Identification and analysis of adenine N(6)-methylation sites in the rice genome. *Nat Plants*, **4**, 554-563.

3. Chen, Z., Zhao, P., Li, F., Marquez-Lago, T.T., Leier, A., Revote, J., Zhu, Y., Powell, D.R., Akutsu, T., Webb, G.I. *et al.* (2020) iLearn: an integrated platform and meta-learner for feature engineering, machine-learning analysis and modeling of DNA, RNA and protein sequence data. *Brief Bioinform*, **21**, 1047-1057.

4. Chen, K., Jiang, Y., Du, L. and Kurgan, L. (2009) Prediction of integral membrane protein type by collocated hydrophobic amino acid pairs. *J Comput Chem*, **30**, 163-172.

5. Chen, K., Kurgan, L.A. and Ruan, J. (2007) Prediction of flexible/rigid regions from protein sequences using k-spaced amino acid pairs. *BMC Struct Biol*, **7**, 25.

6. Saravanan, V. and Gautham, N. (2015) Harnessing Computational Biology for Exact Linear B-Cell Epitope Prediction: A Novel Amino Acid Composition-Based Feature Descriptor. *OMICS*, **19**, 648-658.

7. Cai, C.Z., Han, L.Y., Ji, Z.L., Chen, X. and Chen, Y.Z. (2003) SVM-Prot: Web-based support vector machine software for functional classification of a protein from its primary sequence. *Nucleic Acids Res*, **31**, 3692-3697.

8. Cai, C.Z., Han, L.Y., Ji, Z.L. and Chen, Y.Z. (2004) Enzyme family classification by support vector machines. *Proteins*, **55**, 66-76.

9. Dubchak, I., Muchnik, I., Holbrook, S.R. and Kim, S.H. (1995) Prediction of protein folding class using global description of amino acid sequence. *Proc Natl Acad Sci U S A*, **92**, 8700-8704.

10. Dubchak, I., Muchnik, I., Mayor, C., Dralyuk, I. and Kim, S.H. (1999) Recognition of a protein fold in the context of the Structural Classification of Proteins (SCOP) classification. *Proteins*, **35**, 401-407.

11. Han, L.Y., Cai, C.Z., Lo, S.L., Chung, M.C. and Chen, Y.Z. (2004) Prediction of RNA-binding proteins from primary sequence by a support vector machine approach. *RNA*, **10**, 355-368.

12. Shen, J., Zhang, J., Luo, X., Zhu, W., Yu, K., Chen, K., Li, Y. and Jiang, H. (2007) Predicting protein-protein interactions based only on sequences information. *Proc Natl Acad Sci U S A*, **104**, 4337-4341.

13. Qiang, X., Chen, H., Ye, X., Su, R. and Wei, L. (2018) 【m6A - predictor - muti-species】M6AMRFS: Robust Prediction of N6-Methyladenosine Sites With Sequence-Based Features in Multiple Species. *Front Genet*, **9**, 495.

14. Liu, B., Xu, J., Lan, X., Xu, R., Zhou, J., Wang, X. and Chou, K.C. (2014) iDNA-Prot|dis: identifying DNA-binding proteins by incorporating amino acid distance-pairs and reduced alphabet profile into the general pseudo amino acid composition. *PLoS One*, **9**, e106691.

15. Liu, B., Gao, X. and Zhang, H. (2019) BioSeq-Analysis2.0: an updated platform for analyzing DNA, RNA and protein sequences at sequence level and residue level based on machine learning approaches. *Nucleic Acids Res*, **47**, e127.

16. Feng, Z.P. and Zhang, C.T. (2000) Prediction of membrane protein types based on the hydrophobic index of amino acids. *J Protein Chem*, **19**, 269-275.

17. Lin, Z. and Pan, X.M. (2001) Accurate prediction of protein secondary structural content. *J Protein Chem*, **20**, 217-220.

18. Sokal, R.R. and Thomson, B.A. (2006) Population structure inferred by local spatial autocorrelation: an example from an Amerindian tribal population. *Am J Phys Anthropol*, **129**, 121-131.

19. Horne, D.S. (1988) Prediction of protein helix content from an autocorrelation analysis of sequence hydrophobicities. *Biopolymers*, **27**, 451-477.

20. Liu, B., Liu, F., Fang, L., Wang, X. and Chou, K.C. (2015) repDNA: a Python package to generate various modes of feature vectors for DNA sequences by incorporating user-defined physicochemical properties and sequence-order effects. *Bioinformatics*, **31**, 1307-1309.

21. Dong, Q., Zhou, S. and Guan, J. (2009) A new taxonomy-based protein fold recognition approach based on autocross-covariance transformation. *Bioinformatics*, **25**, 2655-2662.

22. Guo, Y., Yu, L., Wen, Z. and Li, M. (2008) Using support vector machine combined with auto covariance to predict protein-protein interactions from protein sequences. *Nucleic Acids Res*, **36**, 3025-3030.

23. Chou, K.C. (2000) Prediction of protein subcellular locations by incorporating quasi-sequence-order effect. *Biochem Biophys Res Commun*, **278**, 477-483.

24. Chou, K.C. and Cai, Y.D. (2004) Prediction of protein subcellular locations by GO-FunD-PseAA predictor. *Biochem Biophys Res Commun*, **320**, 1236-1239.

25. Schneider, G. and Wrede, P. (1994) The rational design of amino acid sequences by artificial neural networks and simulated molecular evolution: de novo design of an idealized leader peptidase cleavage site. *Biophys J*, **66**, 335-344.

26. Chou, K.C. (2001) Prediction of protein cellular attributes using pseudo-amino acid composition. *Proteins*, **43**, 246-255.

27. Chou, K.C. (2005) Using amphiphilic pseudo amino acid composition to predict enzyme subfamily classes. *Bioinformatics*, **21**, 10-19.

28. Zuo, Y., Li, Y., Chen, Y., Li, G., Yan, Z. and Yang, L. (2017) PseKRAAC: a flexible web server for generating pseudo K-tuple reduced amino acids composition. *Bioinformatics*, **33**, 122-124.

29. Chen, Z., Chen, Y.Z., Wang, X.F., Wang, C., Yan, R.X. and Zhang, Z. (2011) Prediction of ubiquitination sites by using the composition of k-spaced amino acid pairs. *PLoS One*, **6**, e22930.

30. Chen, Z., Zhou, Y., Song, J. and Zhang, Z. (2013) hCKSAAP_UbSite: improved prediction of human ubiquitination sites by exploiting amino acid pattern and properties. *Biochim Biophys Acta*, **1834**, 1461-1467.

31. Wang, J.T.L., Ma, Q., Shasha, D. and Wu, C.H. (2001) New techniques for extracting features from protein sequences. **40**, 426–441.

32. White, G. and Seffens, W. (1998) Using a neural network to backtranslate amino acid sequences. *Electronic Journal of Biotechnology (ISSN: 0717-3458) Vol 1 Num 3*, **1**.

33. Lin, K., May, A.C. and Taylor, W.R. (2002) Amino acid encoding schemes from protein structure alignments: multi-dimensional vectors to describe residue types. *J Theor Biol*, **216**, 361-365.

34. Tung, C.W. and Ho, S.Y. (2008) Computational identification of ubiquitylation sites from protein sequences. *BMC Bioinformatics*, **9**, 310.

35. Lee, T.Y., Chen, S.A., Hung, H.Y. and Ou, Y.Y. (2011) Incorporating distant sequence features and radial basis function networks to identify ubiquitin conjugation sites. *PLoS One*, **6**, e17331.

36. Chen, Y.Z., Chen, Z., Gong, Y.A. and Ying, G. (2012) SUMOhydro: a novel method for the prediction of sumoylation sites based on hydrophobic properties. *PLoS One*, **7**, e39195.

37. Chen, X., Qiu, J.D., Shi, S.P., Suo, S.B., Huang, S.Y. and Liang, R.P. (2013) Incorporating key position and amino acid residue features to identify general and species-specific Ubiquitin conjugation sites. *Bioinformatics*, **29**, 1614-1622.

38. Lee, D., Karchin, R. and Beer, M.A. (2011) Discriminative prediction of mammalian enhancers from DNA sequence. *Genome Res*, **21**, 2167-2180.

39. Noble, W.S., Kuehn, S., Thurman, R., Yu, M. and Stamatoyannopoulos, J. (2005) Predicting the in vivo signature of human gene regulatory sequences. *Bioinformatics*, **21 Suppl 1**, i338-343.

40. Gupta, S., Dennis, J., Thurman, R.E., Kingston, R., Stamatoyannopoulos, J.A. and Noble, W.S. (2008) Predicting human nucleosome occupancy from primary sequence. *PLoS Comput Biol*, **4**, e1000134.

41. Chen, W., Tran, H., Liang, Z., Lin, H. and Zhang, L. (2015) Identification and analysis of the N(6)-methyladenosine in the Saccharomyces cerevisiae transcriptome. *Sci Rep*, **5**, 13859.

42. Qiang, X., Chen, H., Ye, X., Su, R. and Wei, L. (2018) M6AMRFS: Robust Prediction of N6-Methyladenosine Sites With Sequence-Based Features in Multiple Species. *Front Genet*, **9**, 495.

43. Gao, F. and Zhang, C.T. (2004) Comparison of various algorithms for recognizing short coding sequences of human genes. *Bioinformatics*, **20**, 673-681.

44. Doench, J.G., Fusi, N., Sullender, M., Hegde, M., Vaimberg, E.W., Donovan, K.F., Smith, I., Tothova, Z., Wilen, C., Orchard, R. *et al.* (2016) Optimized sgRNA design to maximize activity and minimize off-target effects of CRISPR-Cas9. *Nat Biotechnol*, **34**, 184-191.

45. Cursons, J., Pillman, K.A., Scheer, K.G., Gregory, P.A., Foroutan, M., Hediyeh-Zadeh, S., Toubia, J., Crampin, E.J., Goodall, G.J., Bracken, C.P. *et al.* (2018) Combinatorial Targeting by MicroRNAs Co-ordinates Post-transcriptional Control of EMT. *Cell Syst*, **7**, 77-91 e77.

46. He, W., Jia, C. and Zou, Q. (2019) 4mCPred: machine learning methods for DNA N4-methylcytosine sites prediction. *Bioinformatics*, **35**, 593-601.

47. Lalović, D. and Veljković, V. (1990) The global average DNA base composition of coding regions may be determined by the electron-ion interaction potential. *Biosystems*, **23**, 311-316.

48. Nair, A.S. and Sreenadhan, S.P. (2006) A coding measure scheme employing electron-ion interaction pseudopotential (EIIP). *Bioinformation*, **1**, 197-202.

49. Manavalan, B., Basith, S., Shin, T.H., Lee, D.Y., Wei, L. and Lee, G. (2019) 4mCpred-EL: An Ensemble Learning Framework for Identification of DNA N(4)-methylcytosine Sites in the Mouse Genome. *Cells*, **8**.

50. Wei, L., Su, R., Luan, S., Liao, Z., Manavalan, B., Zou, Q. and Shi, X. (2019) Iterative feature representations improve N4-methylcytosine site prediction. *Bioinformatics*, **35**, 4930-4937.

51. Liu, B., Liu, F., Wang, X., Chen, J., Fang, L. and Chou, K.C. (2015) Pse-in-One: a web server for generating various modes of pseudo components of DNA, RNA, and protein sequences. *Nucleic Acids Res*, **43**, W65-71.

52. Dong, J., Yao, Z.J., Wen, M., Zhu, M.F., Wang, N.N., Miao, H.Y., Lu, A.P., Zeng, W.B. and Cao, D.S. (2016) BioTriangle: a web-accessible platform for generating various molecular representations for chemicals, proteins, DNAs/RNAs and their interactions. *J Cheminform*, **8**, 34.

53. Dong, J., Yao, Z.J., Zhang, L., Luo, F., Lin, Q., Lu, A.P., Chen, A.F. and Cao, D.S. (2018) PyBioMed: a python library for various molecular representations of chemicals, proteins and DNAs and their interactions. *J Cheminform*, **10**, 16.

54. Jain, A.K., Murty, M.N. and Flynn, P.J. (1999) Data clustering: A review. *Acm Computing Surveys*, **31**, 264-323.

55. Rokach, L. and Maimon, O. (2005) In Maimon, O. and Rokach, L. (eds.), *Data Mining and Knowledge Discovery Handbook*. Springer US, Boston, MA, pp. 321-352.

56. Theodoridis, S. and Koutroumbas, K. (2009) In Theodoridis, S. and Koutroumbas, K. (eds.), *Pattern Recognition (Fourth Edition)*. Academic Press, Boston, pp. 653-700.

57. Filippone, M., Camastra, F., Masulli, F. and Rovetta, S. (2008) A survey of kernel and spectral methods for clustering. *Pattern Recognition*, **41**, 176-190.

58. Enright, A.J., Van Dongen, S. and Ouzounis, C.A. (2002) An efficient algorithm for large-scale detection of protein families. *Nucleic Acids Res*, **30**, 1575-1584.

59. Jain, A.K. (2010) Data clustering: 50 years beyond K-means. *Pattern Recognition Letters*, **31**, 651-666.

60. Frey, B.J. and Dueck, D. (2007) Clustering by passing messages between data points. *Science*, **315**, 972-976.

61. Cheng, Y.Z. (1995) Mean Shift, Mode Seeking, and Clustering. *Ieee Transactions on Pattern Analysis and Machine Intelligence*, **17**, 790-799.

62. Ester, M., Kriegel, H.-P., Sander, r. and Xu, X. (1996), *Proceedings of the Second International Conference on Knowledge Discovery and Data Mining*. AAAI Press, Portland, Oregon, pp. 226-231.

63. Pearson, K. (1901) LIII. On lines and planes of closest fit to systems of points in space. *The London, Edinburgh, and Dublin Philosophical Magazine and Journal of Science*, **2**, 559-572.

64. Blei, D.M., Ng, A.Y. and Jordan, M.I. (2003) Latent dirichlet allocation. **3**, 993–1022.

65. Maaten, L.V.D. (2014) Accelerating t-SNE using tree-based algorithms. *J. Mach. Learn. Res.*, **15**, 3221-3245.
