# Supplementary material for: iFeatureOmega: an integrative platform for engineering, visualization and analysis of features from molecular sequences, structural and ligand data sets
Source: Nucleic Acids Res. 2022 May 7;50(W1):W434–47. doi: 10.1093/nar/gkac351 (PMC9252729; doi:10.1093/nar/gkac351)
Supplement: gkac351_Supplemental_Files [file gkac351_supplemental_files.zip › iFeatureOmega_UserManual_R2_CL.docx]

| **User Manual for *iFeatureOmega***  Zhen Chen^1,2,†^, Xuhan Liu^3,†^, Pei Zhao^4,†^, Chen Li^5,†^, Yanan Wang^5^, Fuyi Li^5^, Tatsuya Akutsu^6^, Chris Bain^7^, Robin B. Gasser^8^, Junzhou Li^1^, Zuoren Yang^4,*^_,_ Xin Gao^9,*^, Lukasz Kurgan^10,*^, and Jiangning Song^5,7,*^  ^1^Collaborative Innovation Center of Henan Grain Crops, Henan Agricultural University, Zhengzhou 450046, China, ^2^Center for Crop Genome Engineering, Henan Agricultural University, Zhengzhou 450046, China, ^3^Drug Discovery and Safety, Leiden Academic Centre for Drug Research, Einsteinweg 55, Leiden, 2333 CC, The Netherlands, ^4^State Key Laboratory of Cotton Biology, Institute of Cotton Research of Chinese Academy of Agricultural Sciences (CAAS), Anyang, 455000, China, ^5^Monash Biomedicine Discovery Institute and Department of Biochemistry and Molecular Biology, Monash University, Melbourne, Victoria 3800, Australia, ^6^Bioinformatics Center, Institute for Chemical Research, Kyoto University, Kyoto 611-0011, Japan ^7^Monash Data Future Institutes, Monash University, Melbourne, Victoria 3800, Australia, ^8^Department of Veterinary Biosciences, Melbourne Veterinary School, The University of Melbourne, Parkville, Victoria 3010, Australia, ^9^Computational Bioscience Research Center (CBRC), Computer, Electrical and Mathematical Sciences and Engineering Division, King Abdullah University of Science and Technology (KAUST), Thuwal 23955, Saudi Arabia, ^10^Department of Computer Science, Virginia Commonwealth University, Richmond, VA, USA  ^†^These authors contributed equally to this work.  ^*^To whom the correspondence should be addressed.  Jiangning Song: Tel: +61-3-9902-9304; Email: [Jiangning.Song@monash.edu](mailto:Jiangning.Song@monash.edu), or Lukasz Kurgan: Tel: +1-804-827-3986; Email: [lkurgan@vcu.edu](mailto:lkurgan@vcu.edu), or Zuoren Yang: Tel: +86-371-5591-2760; Email: [yangzuoren@caas.cn](mailto:yangzuoren@caas.cn), or Xin Gao: Tel: +966-128080323; E-mail: [xin.gao@kaust.edu.sa](mailto:xin.gao@kaust.edu.sa). |
| --- |

Table of Contents

[1. Introduction 3](#_Toc100043347)

[2. Installing and running iFeatureOmega 3](#_Toc100043348)

[Installation 3](#_Toc100043349)

[Running for GUI version 4](#_Toc100043350)

[Running for CLI version 4](#_Toc100043351)

[3. The workflow of iFeatureOmega-GUI 5](#_Toc100043352)

[4. The workflow of iFeatureOmega-CLI 6](#_Toc100043353)

[5. The input and output format of iFeatureOmega 6](#_Toc100043354)

[6. iFeatureOmega-GUI usage 6](#_Toc100043355)

[DNA/RNA/Protein sequences feature descriptor extraction 6](#_Toc100043356)

[Protein structure feature descriptor extraction 8](#_Toc100043357)

[Ligands feature descriptor extraction 8](#_Toc100043358)

[Feature analysis 8](#_Toc100043359)

[Plot with user-defined data 10](#_Toc100043360)

[7. iFeatureOmega-CLI usage 11](#_Toc100043361)

[Extract feature descriptors 11](#_Toc100043362)

[Feature analysis 14](#_Toc100043363)

[Data visualization 16](#_Toc100043364)

[8. Online web server 22](#_Toc100043365)

[9. List of Integrated Feature Descriptors, Feature Analysis Plots in iFeatureOmega 31](#_Toc100043366)

[10. Descriptions of feature descriptors for nucleotide sequences 35](#_Toc100043367)

[11. Commonly Used Feature Descriptors and Parameters for Protein or Peptide Sequences 55](#_Toc100043368)

[12. Commonly Used Feature Descriptors and Parameters for Ligands 77](#_Toc100043369)

[13. Commonly Used Feature Descriptors and Parameters for Protein Structure 79](#_Toc100043370)

[14. References 83](#_Toc100043371)

# 1. Introduction

*iFeatureOmega* is a comprehensive platform for generating, analyzing and visualizing 189 representations for biological sequences, 3D structures and ligands. To the best of our knowledge, *iFeatureOmega* supplies the largest number of feature extraction and analysis approaches for most molecule types compared to other pipelines. Three versions (i.e. *iFeatureOmega-Web*, *iFeatureOmega-GUI* and *iFeatureOmega-CLI*) of *iFeatureOmega* have been made available to cater to both experienced bioinformaticians and biologists with limited programming expertise. *iFeatureOmega* also expands its functionality by integrating 15 feature analysis algorithms (including ten cluster algorithms, three dimensionality reduction algorithms and two feature normalization algorithms) and providing nine types of interactive plots for statistical features visualization (including histogram, kernel density plot, heatmap, boxplot, line chart, scatter plot, circular plot, protein three dimensional structure plot and ligand structure plot). *iFeatureOmega* is an open-source platform for academic purposes. The web server can be accessed through <http://ifeatureomega.erc.monash.edu> and the GUI and CTL versions can be download at: <https://github.com/Superzchen/iFeatureOmega-GUI> and <https://github.com/Superzchen/iFeatureOmega-CLI>, respectively.

# 2. Installing and running *iFeatureOmega*

## Installation

*iFeatureOmega* is an open-source Python-based toolkit, which operates in the Python environment (Python version 3.7.4) and can run on multiple operating systems (e.g. Windows, Mac, and Linux). Prior to installing and running *iFeatureOmega*, all the third-party packages should be installed in the Python environment, including Biopython (v.1.78), Pandas (v.1.3.4), Numpy (v.1.21.4), NetworkX (v.2.5), RDKit (v. 2020.03.3.0), Matplotlib (v.3.4.3), DSSP (v.3.0.0) and MSMS (v.2.6.1). The GUI of *iFeatureOmega-GUI* was implemented by PyQt5 (v.5.9.2). For convenience, we strongly recommend users install the Anaconda Python environment in their local computers, which can be freely downloaded from <https://www.anaconda.com/>. The detailed steps of installing these dependencies are provided as follows:

Step 1. Download and install the anaconda platform:

- Download from: <https://repo.anaconda.com/archive/> and download the software package (version: 2020.02) according to your operating system.

Step 2. Install anaconda environment on your own machine and make sure the “Add Anaconda to the system PATH environment variable” option is selected when installation.

Step 3. Install biopython, rdkit, dssp, msms and networkx

$ conda install biopython

$ conda install -c rdkit rdkit

$ conda install -c saliab dssp

$ conda install -c bioconda msms

$ conda install networkx

**Note:** dssp and msms can not be installed in Windows system.

Step 4. Install *iFeatureOmega* by pip:

- *For GUI version:*

$ pip3 install iFeatureOmegaGUI

- *For CLI version:*
- $ pip3 install iFeatureOmegaCLI

## Running for GUI version

To run *iFeatureOmega-GUI*:

$ python

>>> from iFeatureOmegaGUI import runiFeatureOmegaGUI

>>> runiFeatureOmegaGUI()

Once *iFeatureOmega* has started, the interface will show as demonstrated in **Figure 1**.

## Running for CLI version

The CLI version of *iFeatureOmega* can be import as a python package as follows:

$ python

>>> import iFeatureOmegaCLI

>>> dna = iFeatureOmegaCLI.iDNA('<abs_path>/data_examples/DNA_sequences.txt')

>>> dna.import_parameters('<abs_path>/parameters/DNA_parameters_setting.json')

>>> dna.get_descriptor('Kmer')

Note: <abs_path> indicates the absolute path of iFeatureOmegaCLI package.


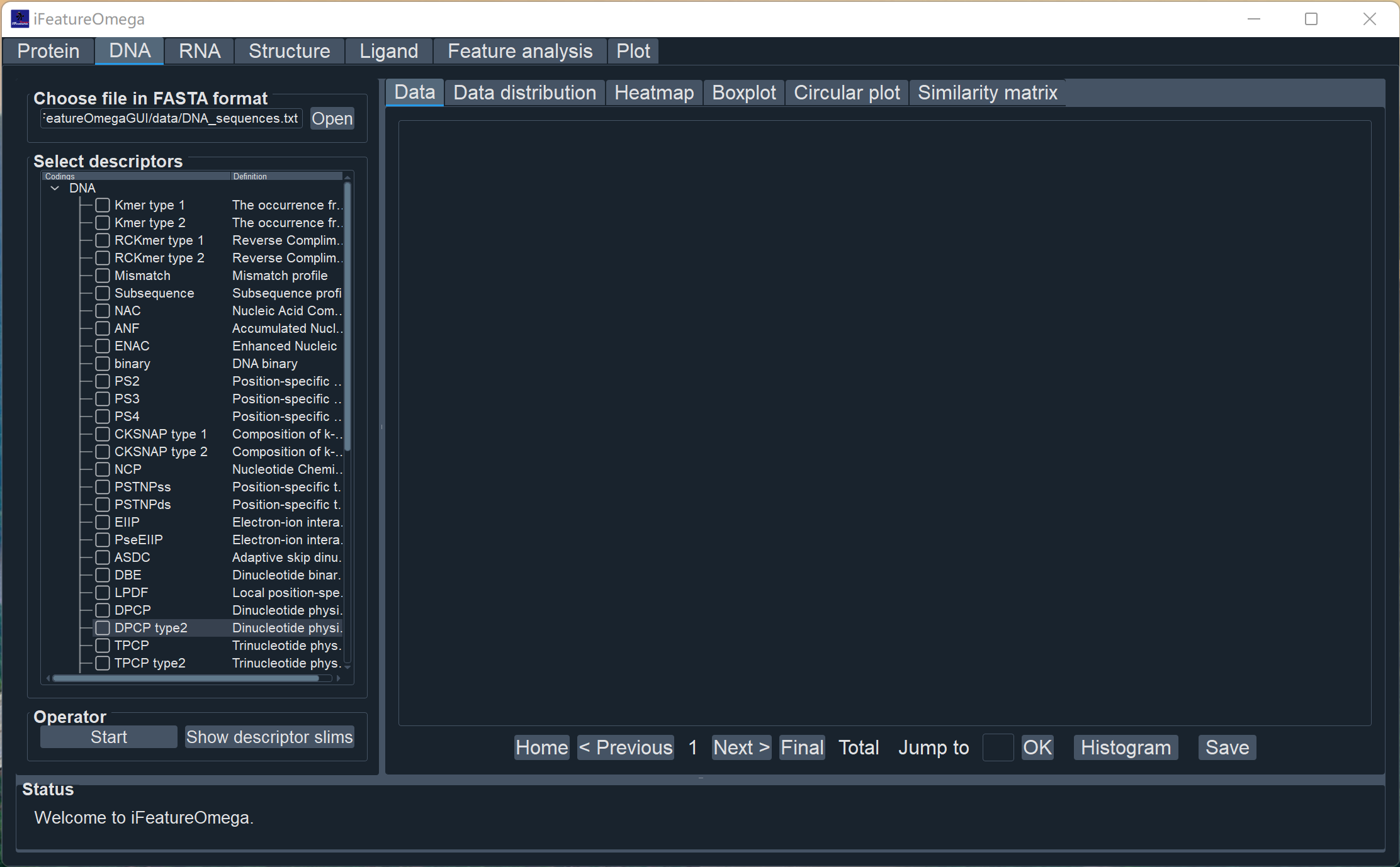


**Figure 1.** The main interface of *iFeatureOmega-GUI*.

# 3. The workflow of *iFeatureOmega-GUI*

Here we provide a step-by-step user instruction to demonstrate the workflow of *iFeatureOmega-GUI* toolkit by running the examples provided in the “examples” directory. Seven tab-widgets are included in the GUI-based version of *iFeatureOmega* (**Figure 1**), and different functionalities are accessible by switching among the tabs. (**Table 1**).

**Table 1**. Functions of the seven tab-widgets in *iFeatureOmega*.

| **Tab-widgets** | **Function** |
| --- | --- |
| *Protein* | 1. Extraction of 71 different types of feature descriptors for protein sequences. |
|  | 1. Data visualization (Histogram and kernel density plot, heatmap, boxplot and circular plot) |
| *DNA* | 1. Extraction of 49 different types of feature descriptors for DNA sequences. |
|  | 1. Data visualization (Histogram and kernel density plot, heatmap, boxplot and circular plot) |
| *RNA* | 1. Extraction of 37 different types of feature descriptors for RNA sequences. 2. Data visualization (Histogram and kernel density plot, heatmap, boxplot and circular plot) |
| *Structure* | 1. Extraction of 14 different types of feature descriptors for protein structures. 2. Data visualization (Histogram and kernel density plot, heatmap, boxplot and circular plot) |
| *Ligand* | 1. Extraction of 18 different types of feature descriptors for protein structures. 2. Data visualization (histogram and kernel density plot, heatmap, Boxplot and circular plot) |
| *Feature analysis* | 1. 15 feature analysis algorithms (ten feature clustering, three dimensionality reduction, and two feature normalization algorithms). 2. Data visualization (Scatter plot, kernel density plot) |
| *Plot* | 1. Generate histogram and kernel density plot, boxplot, heatmap, scatter plot and circular plot by using user-defined data. |

# 4. The workflow of *iFeatureOmega-CLI*

There were seven main python class in *iFeatureOmega-CLI*, including “iProtein”, “iDNA”, “iRNA”, “iStructure” and “iLigand”, which are feature extraction methods for protein sequences, DNA sequences, RNA sequences, protein structures and ligand molecules, respectively. “iAnalysis” class is also equipped for feature analysis, while “iPlot” class is used to generate the corresponding plots.

# 5. The input and output format of *iFeatureOmega*

To calculate features for protein, DNA and RNA sequences, a set of DNA, RNA or protein sequences in FASTA format is used as input. To calculate analyze protein structural features, users need to provide the protein structure of interest, which must comply with the PDB(1) (https://www.rcsb.org/) format by either providing a PDB accession or uploading your own structure file (i.e., in PDB or CIF format). For analyzing ligands, candidate ligand molecules can be provided with SMILES format or SDF format.

# 6. *iFeatureOmega-GUI* usage

Seven tab-widgets are included and different functionalities are accessible by switching among tabs.

## DNA/RNA/Protein sequences feature descriptor extraction

Taking feature descriptor extraction of DNA sequences as an example:

*Step 1: Open the sequences file*

Click the “Open” button and select the DNA sequences file (e.g. “DNA_sequences.txt” in “data” directory of *iFeatureOmega* package).

*Step 2: Select the feature descriptor and configure the descriptor parameters*

Select one or more feature descriptor(s) and set the corresponding parameters with the parameter dialog box.

*Step 3: Run the program*

Click the “Start” button to calculate the descriptor features. The feature encoding and graphical presentation will be displayed in the “Data”, “Data distribution”, “Heatmap”, “Boxplot” and “Circular plot” panels, respectively (**Figure 2**).

*Step 4: Save the results and plots*

Click the “Save” button to save the generated feature encodings. *iFeatureOmega* supports four formats for saving the calculated features, including LIBSVM, CSV, TSV, and WEKA format, so as to facilitate direct use of the features in the following analysis, prediction model construction and the third-party computational tools, such as scikit-learn and WEKA. In addition, *iFeatureOmega* provides the TSV1 format, which includes the sample and feature labels. All the plots in *iFeatureOmega* are generated by the matplotlib library and can be saved to a variety of image formats, such as PNG, JPG, PDF, TIFF etc).


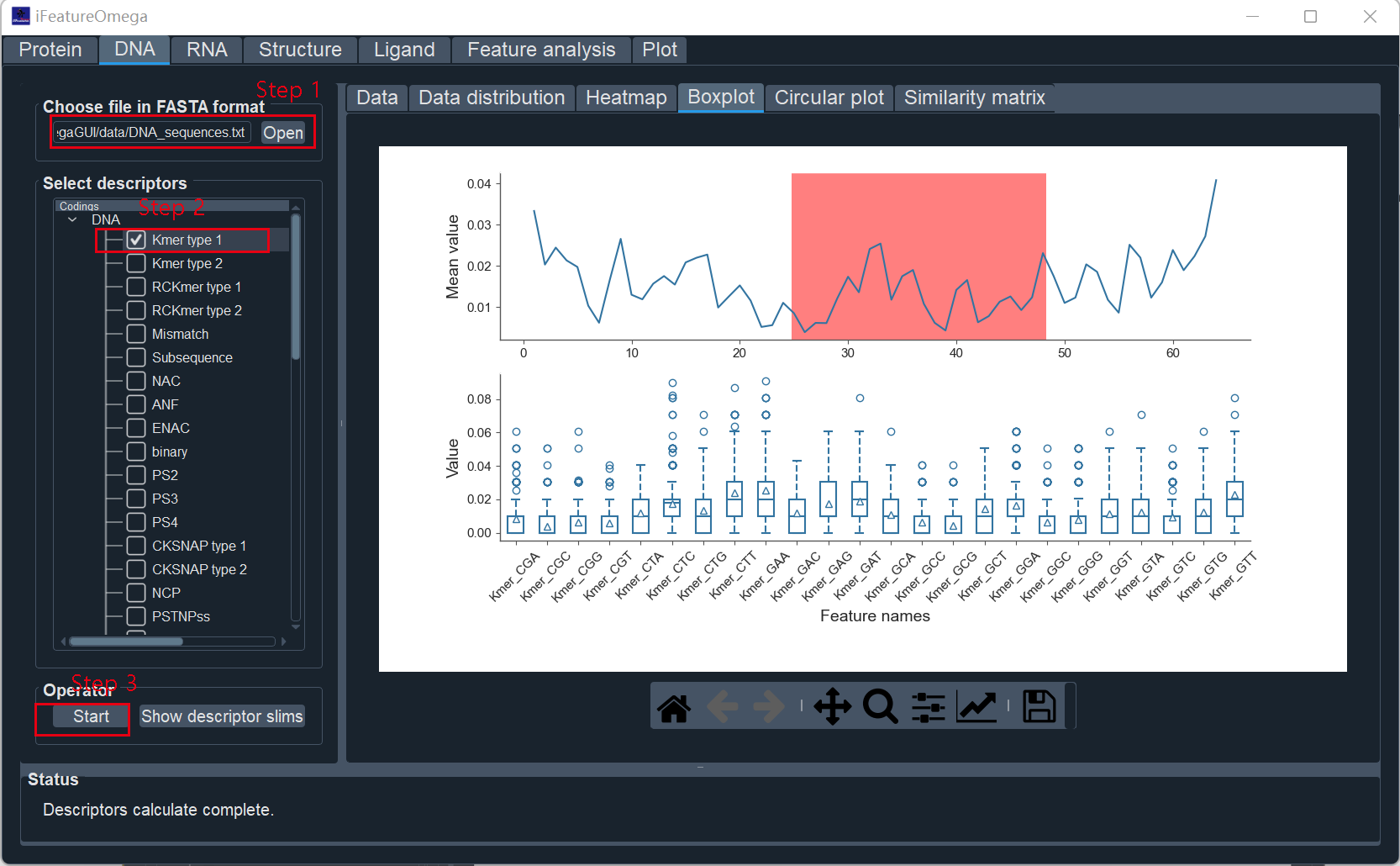


**Figure 2**. An example of extracting feature descriptor.

## Protein structure feature descriptor extraction

*Step 1: Open a PDB file or enter PDB ID*

Click the “Open” button and select a PDB file (e.g. “1iir.pdb” in “data” directory of *iFeatureOmega* package), or enter a PDB ID (e.g. “1iir”) and then click the “Download” button, then the program will download the PDB file automatically.

*Step 2: Select the feature descriptor type and run the program.*

Please refer to the steps 2-4 in “DNA/RNA/Protein sequences feature descriptor extraction”.

## Ligands feature descriptor extraction

*Step 1: Open a file with SMILES format*

Click the “Open” button and select a file (e.g. “Chemical_SMILES.txt” in “data” directory of *iFeatureOmega* package). In this file, each row is a molecule with SMILES format (**Figure 3**).

*Step 2: Select the feature descriptor type and run the program.*

Please refer to the steps step 2-4 in “DNA/RNA/Protein sequences feature descriptor extraction”.


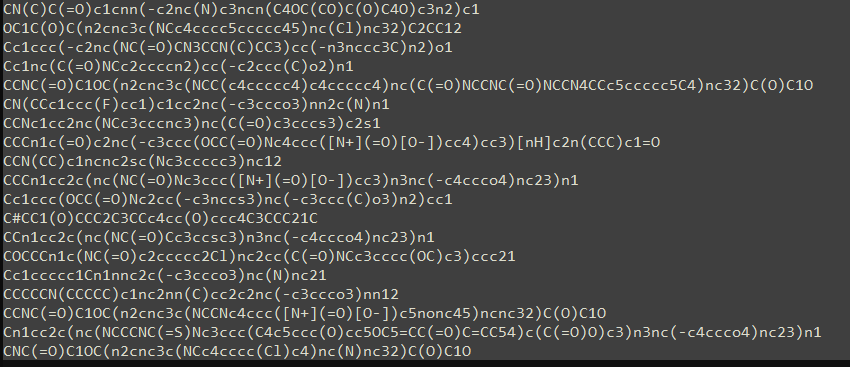


**Figure 3.** An example of the input file for ligand feature descriptor extraction.

## Feature analysis

*iFeatureOmega* provides multiple options to facilitate feature analysis, including ten feature clustering, three dimensionality reduction and two feature normalization (**Table S5** in our paper). The “Feature analysis” panel is used to deploy the feature analysis function. Taking the clustering as an example:

*Step 1: Load data*

There are two ways to load the data for analysis: 1) open a coding file and 2) select the data generated from other panels. Here we load the data from file. Click the “Open [without sample label]” button and open the “data_without_labels.csv” in the “data” directory.

*Step 2: Select the analysis algorithm and set the corresponding parameter(s)*

Select “kmeans” clustering algorithm and set the cluster number as 3.

*Step 3: Run the program*

Click the “Start” button to start the analysis progress. The clustering result and graphical presentation will be displayed in the “Cluster/Dimensionality reduction result” and “Scatter plot” panels, respectively. Here, we used the scatter plot to display the clustering result.

*Step 4: Save the result and plot*

Click the “Save” button to save the generated clustering results (**Figure 4**).


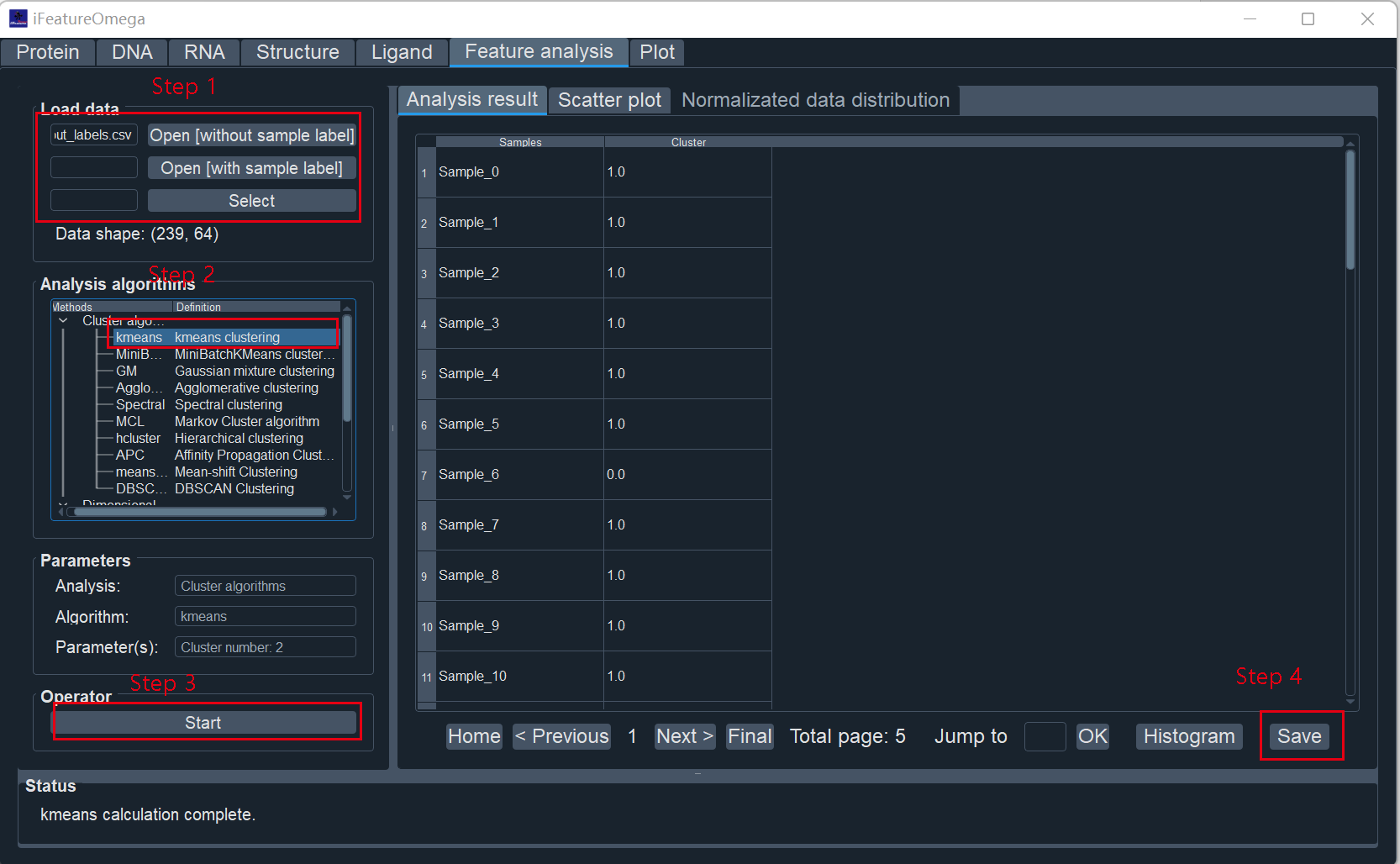


**Figure 4**. An example of implement the clustering algorithm using *iFeatureOmega*.

For feature dimensionality reduction analysis, *iFeatureOmega* also support data with sample labels (i.e. The sample labels should be integers in the first column of the data file):

*Step 1: Load data* *with sample labels for dimensionality reduction analysis*

Click the “Open [with sample label]” button and open the “data_with_labels.csv” in the “data” directory.

*Step 2: Select the analysis algorithm and set the corresponding parameter(s)*

Select “PCA” algorithm and set the cluster number as 2.

*Step 3: Run the program*

Click the “Start” button to start the analysis progress. The dimensionality reduction result and graphical presentation will be displayed in the “Cluster/Dimensionality reduction result” and “Scatter plot” panels, respectively. Each dot represents a sample, and the color of the dots corresponds to the sample category (i.e. Samples with the same label have the same color).

*Step 4: Save the result and plot*

Click the “Save” button to save the generated clustering results.

## Plot with user-defined data

For convenience, *iFeatureOmega* allows generate plots with user-defined data. Take the box plot as an example:

*Step 1: Load data*

Click the “Open [with sample label]” button and open the “data_with_labels.csv” in the “data” directory.

*Step 2: Select plot type*

Select “Box plot”.

*Step 3: Run the program*

Click the “Draw plot” button to start the analysis progress. The plot will be displayed in the “Plot viewer” panel.


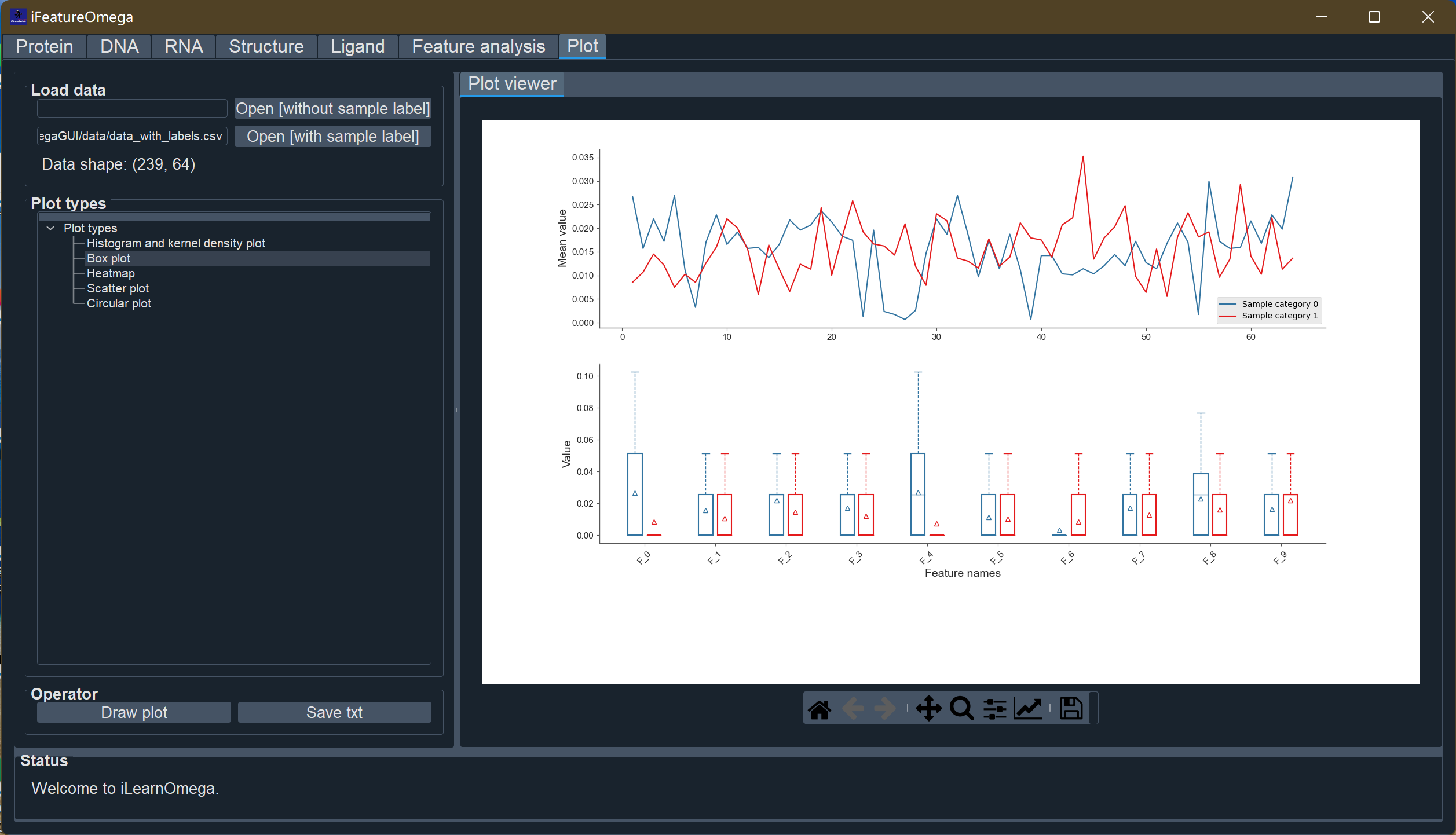


**Figure 5**. An example of boxplot generated by *iFeatureOmega*.

# 7. *iFeatureOmega-CLI* usage

The CLI-based version, which was developed and implemented as a python package. This version accepts the JSON format configuration file, allowing users to conveniently specify the parameter values of individual features and analysis algorithms.

The classes in iFeatureOmega-CLI are:

- *iDNA*: extract feature descriptors for DNA sequences
- *iRNA*: extract feature descriptors for RNA sequences
- *iProtein*: extract feature descriptors for protein sequences
- *iLigand*: extract feature descriptors for chemical molecules.
- *iStructure*: extract feature descriptors for protein structure
- *iAnalysis*: implement feature analysis for extracted feature descriptors
- *iPlot*: generate various statistical plots for generated feature descriptors

## Extract feature descriptors

In each class, we provide the example code, users can run the following command view the example code for “iDNA” class.

$ python

>>> import iFeatureOmegaCLI

>>> print(iFeatureOmegaCLI.iDNA.__doc__)

Take the DNA feature descriptors extraction as an example:

*Step 1: running python and import iFeatureOmega*

Run python from the command line.

*Step 2: import iFeatureOmega*

$ python

>>> import iFeatureOmegaCLI

*Step 3: Load data file*

>>> dna = iFeatureOmegaCLI.iDNA("<abs_path>/data_examples/DNA_sequences.txt")

“iDNA” is used to extract feature descriptors for DNA sequences, and the class should be initialized with the sequence file (i.e. “<abs_path>/data_examples/DNA_sequences.txt” in this example, <abs_path> indicates the absolute path of “data_examples” directory.)

*Step 4: Display the available feature descriptor types*

For “iDNA”, “iRNA”, “iProtein”, “iLigand” and “iStructure”, each of these classes has a function named “display_feature_types”, which is used to display the names of available feature extraction methods (**Figure 6**).


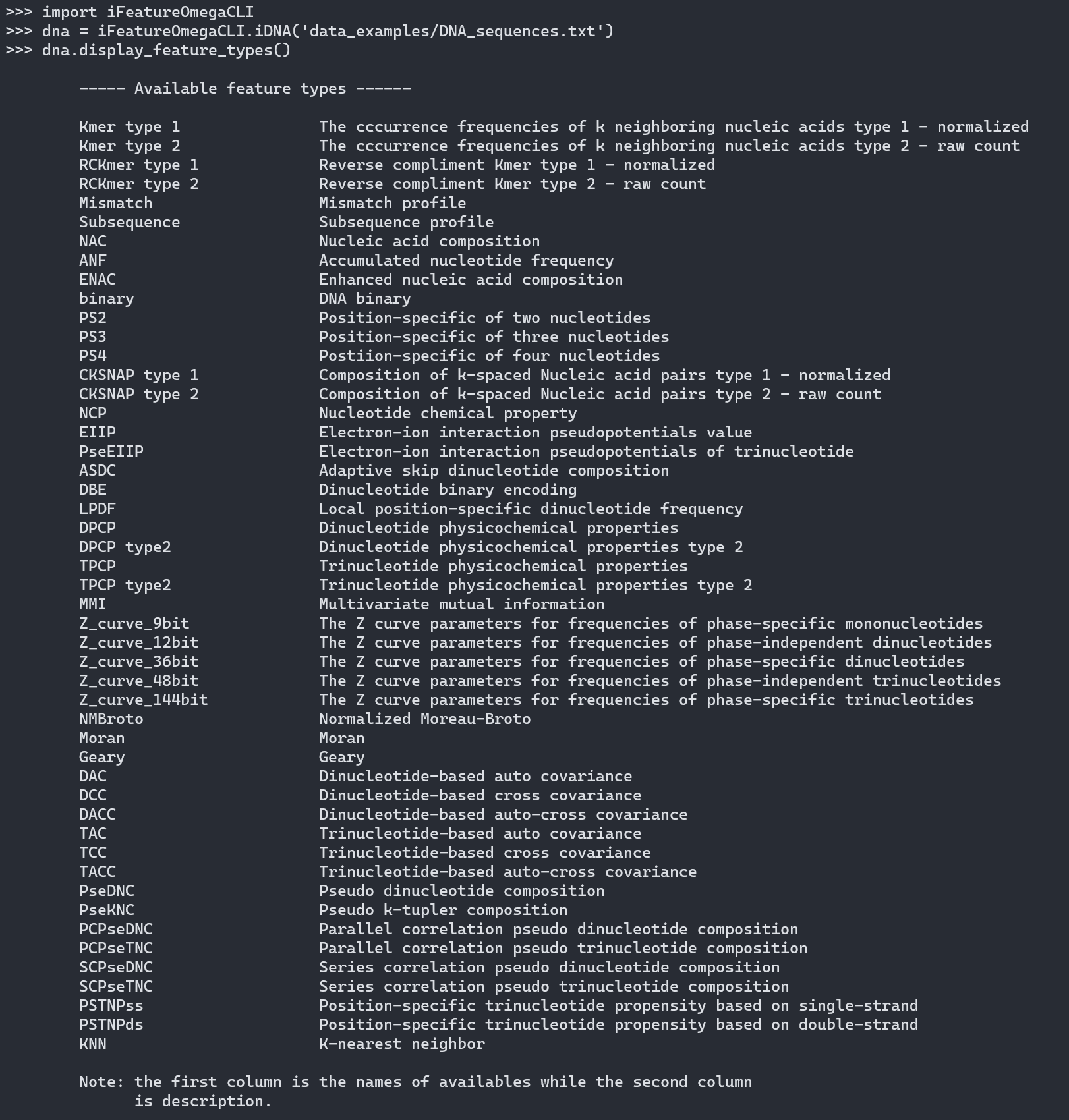


**Figure 6.** A snapshot of the available feature extraction methods in “iDNA” class.

*Step 5: Extract feature descriptor*

We use the “get_descriptor” function to extract the feature descriptors. Take the “Kmer” feature descriptor as an example (**Figure 7**):


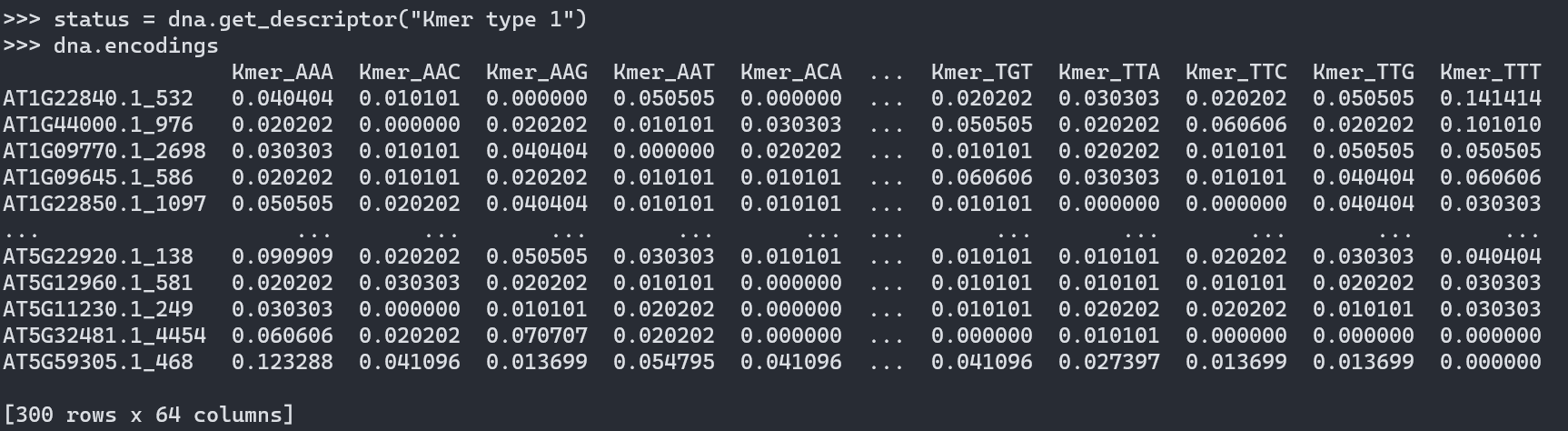


**Figure 7.** A snapshot of DNA feature descriptor extraction.

The calculated feature descriptors will be stored in the “encodings” variable of the “iDNA” class, which is a pandas dataframe.

*Step 6: Save the feature descriptor values to file*

Four functions including “to_csv”, “to_tsv”, “to_svm” and “to_arff”, are provided to save the calculated feature descriptor values to the “CSV”, “TSV”, “SVM” and “arff”, respectively.

>>> dna.to_csv("Kmer.csv", index=False, header=False)

## Feature analysis

Run the following command to view the example code for feature analysis (**Figure 8**):

>>> print(iFeatureOmegaCLI.iAnalysis.__doc__)


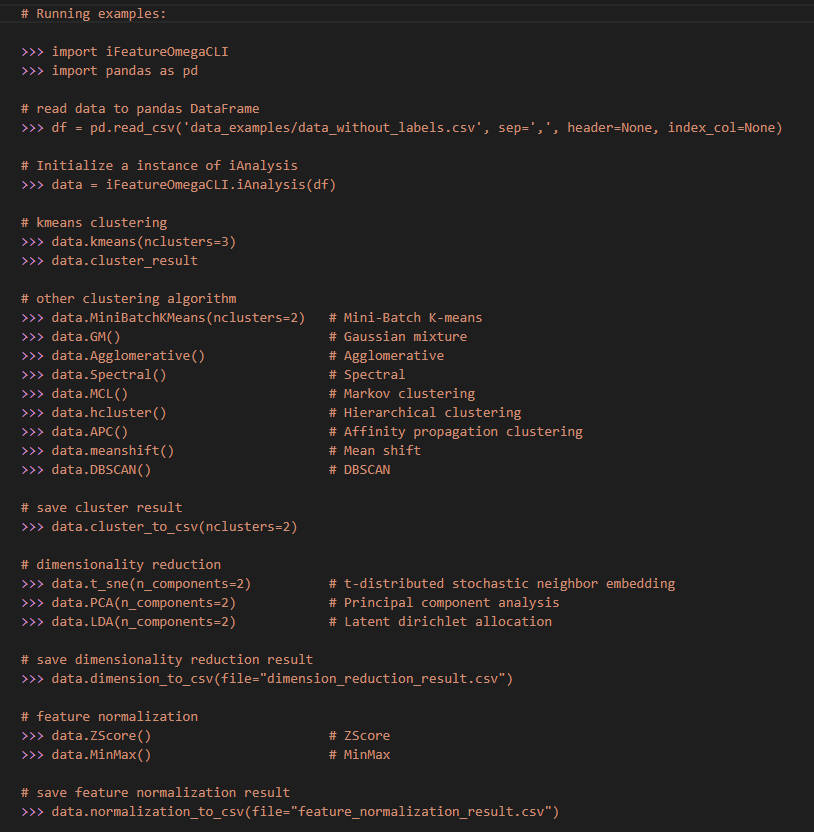


**Figure 8.** A snapshot of running example for “iAnalysis” class.

*Step 1: Open a data file*

>>> import iFeatureOmegaCLI

>>> import pandas as pd

# read data to pandas DataFrame

>>> df = pd.read_csv('data_examples/data_without_labels.csv', sep=',', header=None, index_col=None)

*Step 2: Create an instance for “iAnalysis” class*

# Initialize an instance of iAnalysis

>>> data = iFeatureOmegaCLI.iAnalysis(df)

*Step 3: Implement an analysis algorithm and save analysis result*

>>> data.kmeans(nclusters=3) # k-means clustering

>>> data.MiniBatchKMeans(nclusters=2) # Mini-Batch K-means

>>> data.GM() # Gaussian mixture

>>> data.Agglomerative() # Agglomerative

>>> data.Spectral() # Spectral

>>> data.MCL() # Markov clustering

>>> data.hcluster() # Hierarchical clustering

>>> data.APC() # Affinity propagation clustering

>>> data.meanshift() # Mean shift

>>> data.DBSCAN() # DBSCAN

# save cluster result

>>> data.cluster_to_csv(nclusters=2)

# dimensionality reduction

>>> data.t_sne(n_components=2) # t-distributed stochastic neighbor embedding

>>> data.PCA(n_components=2) # Principal component analysis

>>> data.LDA(n_components=2) # Latent dirichlet allocation

# save dimensionality reduction result

>>> data.dimension_to_csv(file="dimension_reduction_result.csv")

# feature normalization

>>> data.ZScore() # ZScore

>>> data.MinMax() # MinMax

# save feature normalization result

>>> data.normalization_to_csv(file="feature_normalization_result.csv")

## Data visualization

Run the following command to view the running code for data visualization (**Figure 9**):

>>> print(iFeatureOmegaCLI.iPlot.__doc__)


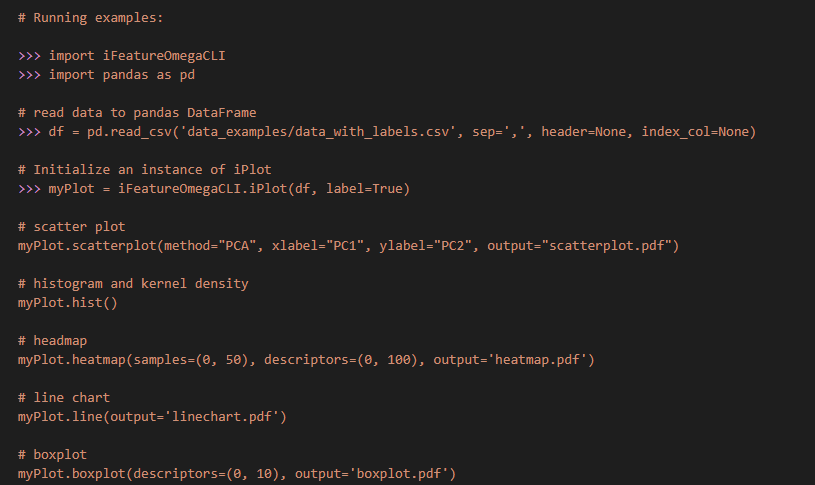


**Figure 9.** A snapshot of running example for “iPlot” class.

*Step 1: Open a data file*

>>> df = pd.read_csv('data_examples/data_with_labels.csv', sep=',', header=None, index_col=None)

*Step 2. Initialize an instance of “iPlot”*

>>> myPlot = iFeatureOmegaCLI.iPlot(df, label=True)

Note: The first column of the data file we be taken as sample labels when label is True.

*Step 3. Scatter plot*

# scatter plot

myPlot.scatterplot(method="PCA", xlabel="PC1", ylabel="PC2", output="scatterplot.pdf")

For scatter plot, the dimensionality reduction algorithm was used to decompose the data to two-dimensional space. And the dimension reduced data will be plotted as points in a plane. Three dimensionality reduction algorithms can be selected (i.e. PCA, t_sne and LDA).

The parameters of “scatterplot”:

- *method*: dimensionality reduction algorithm (select from PCA, t_sne, LDA)
- *xlabel*: the name of abscissa label of scatter plot
- *ylabel*: the name of ordinate label of scatter plot
- *output*: the name of output file


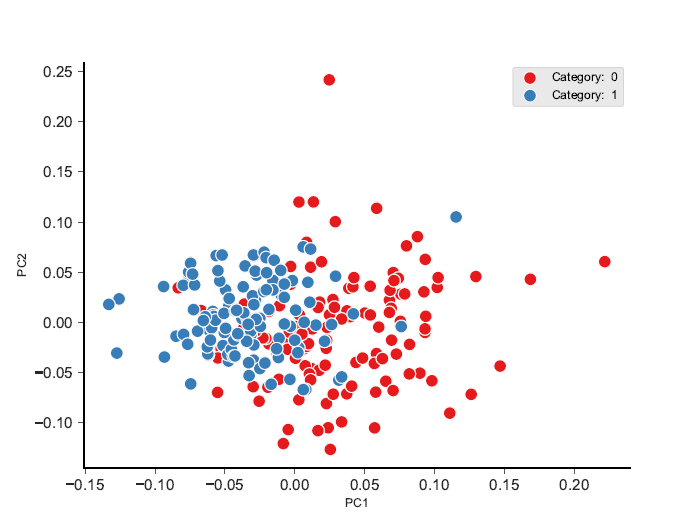


**Figure 10.** An example of scatter plot.

Step 4. *Histogram and kernel density plot*

# histogram and kernel density

myPlot.hist()


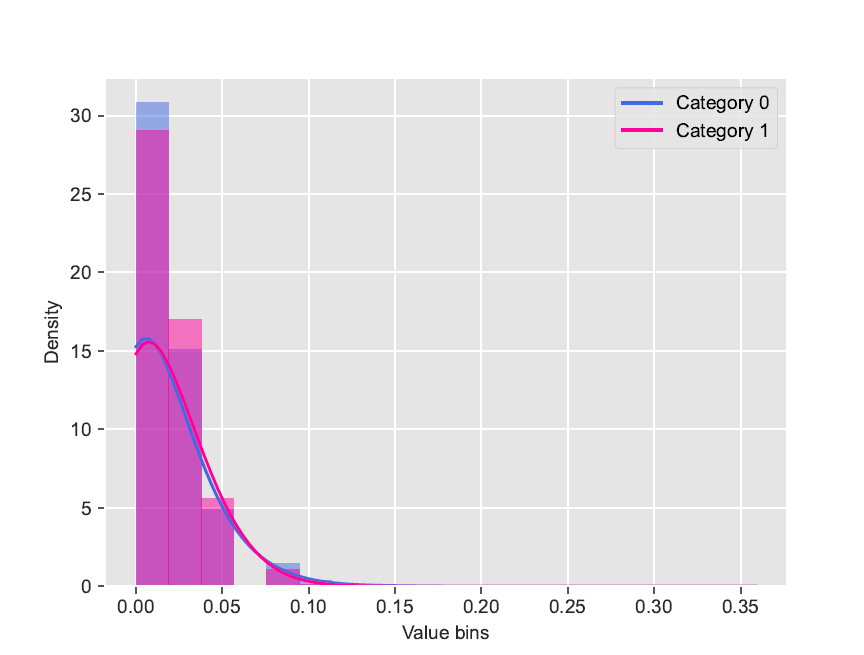


**Figure 11.** An example of histogram and kernel density plot.

*Step 5. Heatmap*

# heatmap

myPlot.heatmap(samples=(0, 50), descriptors=(0, 100), output='heatmap.pdf')

The parameters of “heatmap”:

- *samples*: the range of displayed samples (i.e. rows of the data matrix)
- *descriptors*: the range of displayed descriptors (i.e. columns of the data matrix)
- *output*: the name of output file


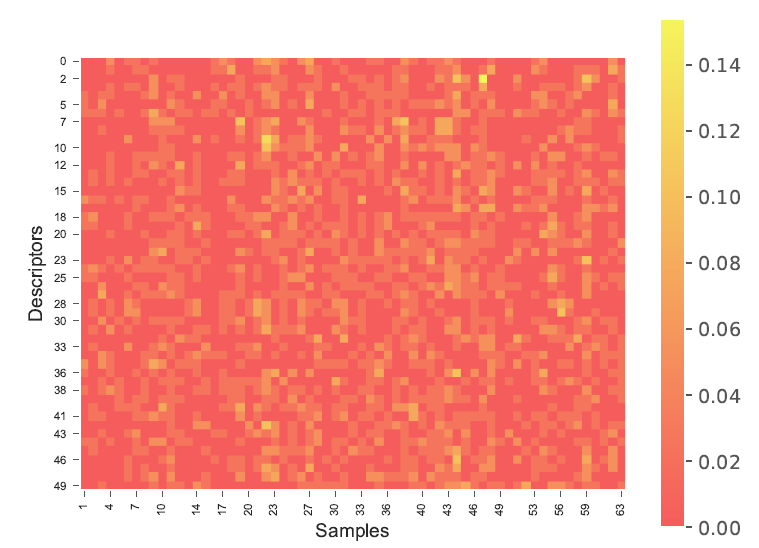


**Figure 12.** An example of heatmap.

*Step 6. Line chart*

# line chart

myPlot.line(output='linechart.pdf')


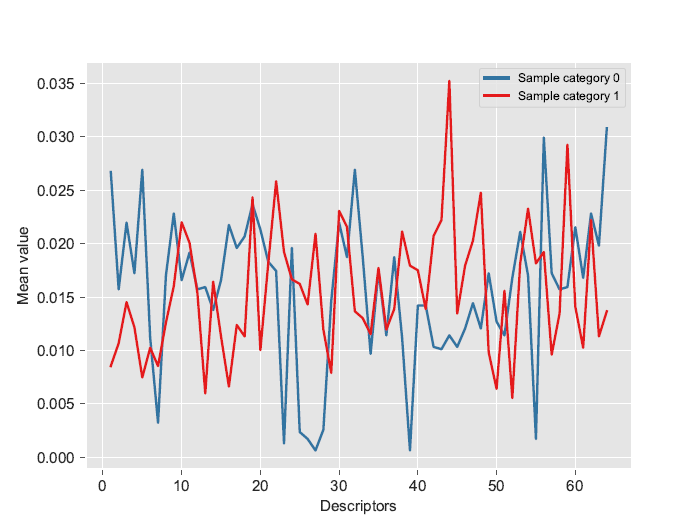


**Figure 13.** An example of line chart.

*Step 7. Boxplot*

# boxplot

myPlot.boxplot(descriptors=(0, 10), output='boxplot.pdf')

The parameters of “boxplot”:

- *descriptors*: the range of displayed descriptors (i.e. columns of the data matrix)


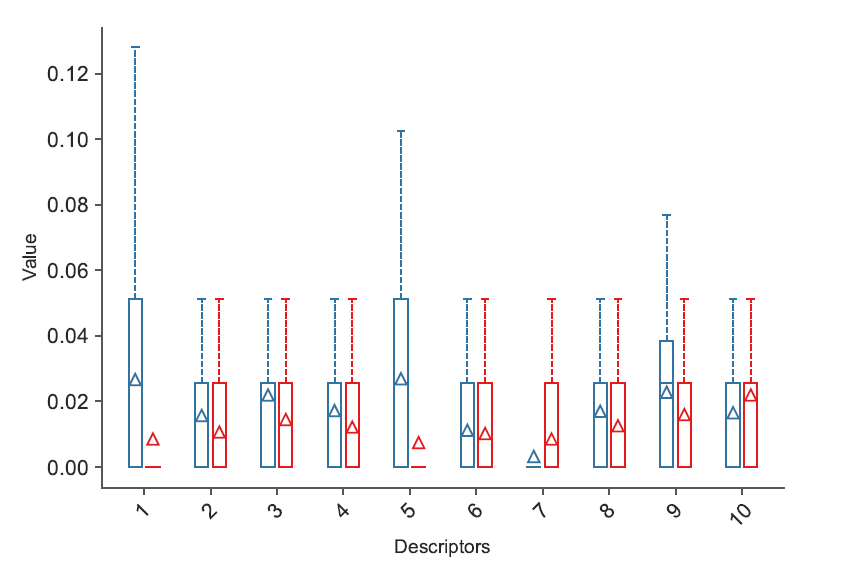


**Figure 14**. An example of boxplot.

# 8. Online web server

The *iFeatureOmega* server is freely accessible at <https://ifeatureomega.erc.monash.edu/>, which resides on the Nectar (The National eResearch Collaboration Tools and Resources) infrastructure and managed by the eResearch Centre at Monash University. The *iFeatureOmega* server was implemented based on ‘Linux+Apache+Django’ framework and is equipped with 16 cores, 64 GB memory and a 2 TB hard disk. The server has been tested across five commonly used browsers, including Internet Explorer (version ≥7.0), Microsoft Edge, Mozilla Firefox, Google Chrome and Safari. The step-by-step of usage instructions is as follows:

Type “<http://ifeatureomega.erc.monash.edu>” on your browser.
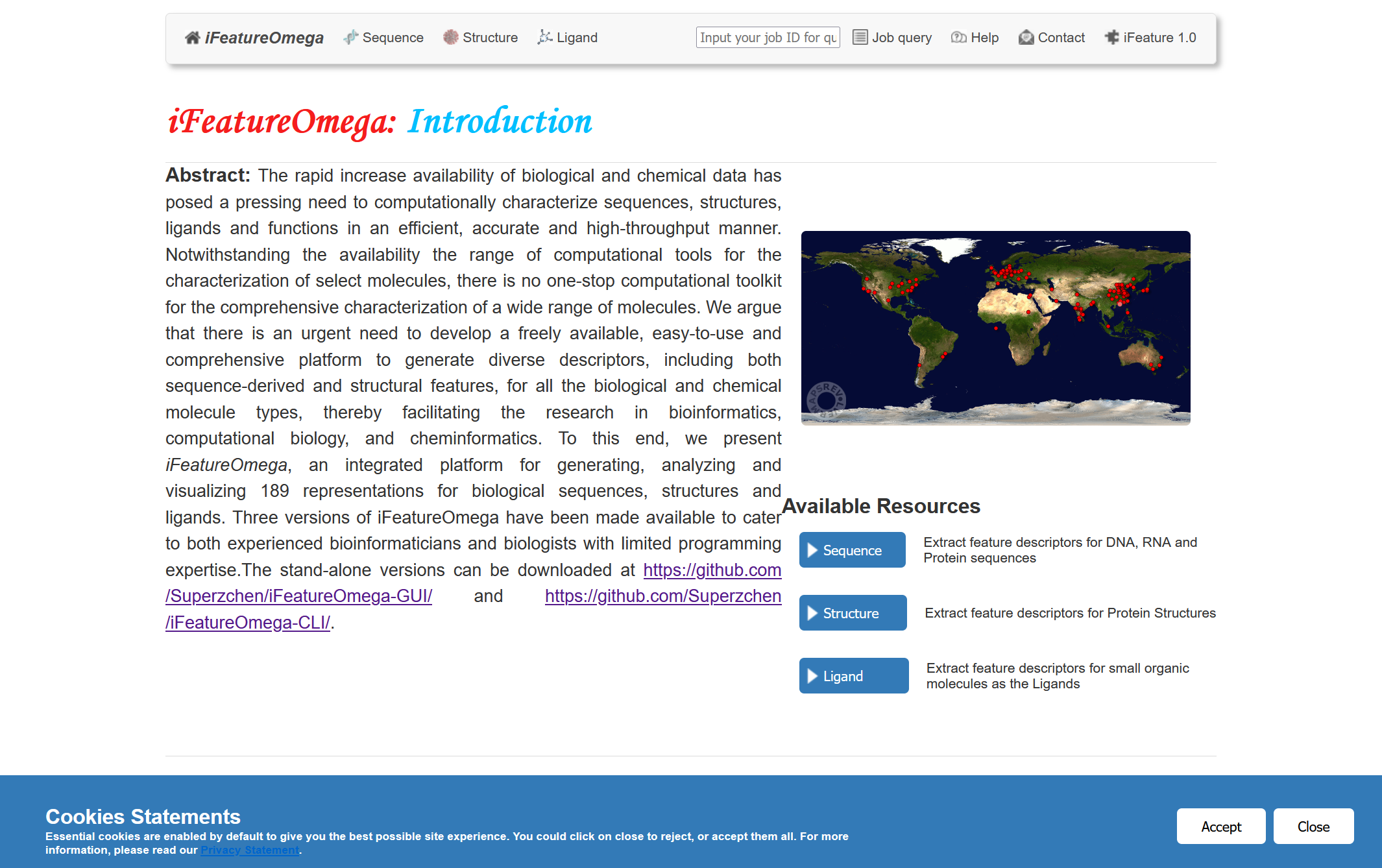


Five webpages have been designed and implemented and are accessible via the navigation bar at the top of the website.

Take ligand page as an example:

*Step 1: Click “Ligand” in the navigation bar and open the page for ligand molecule feature extraction.*


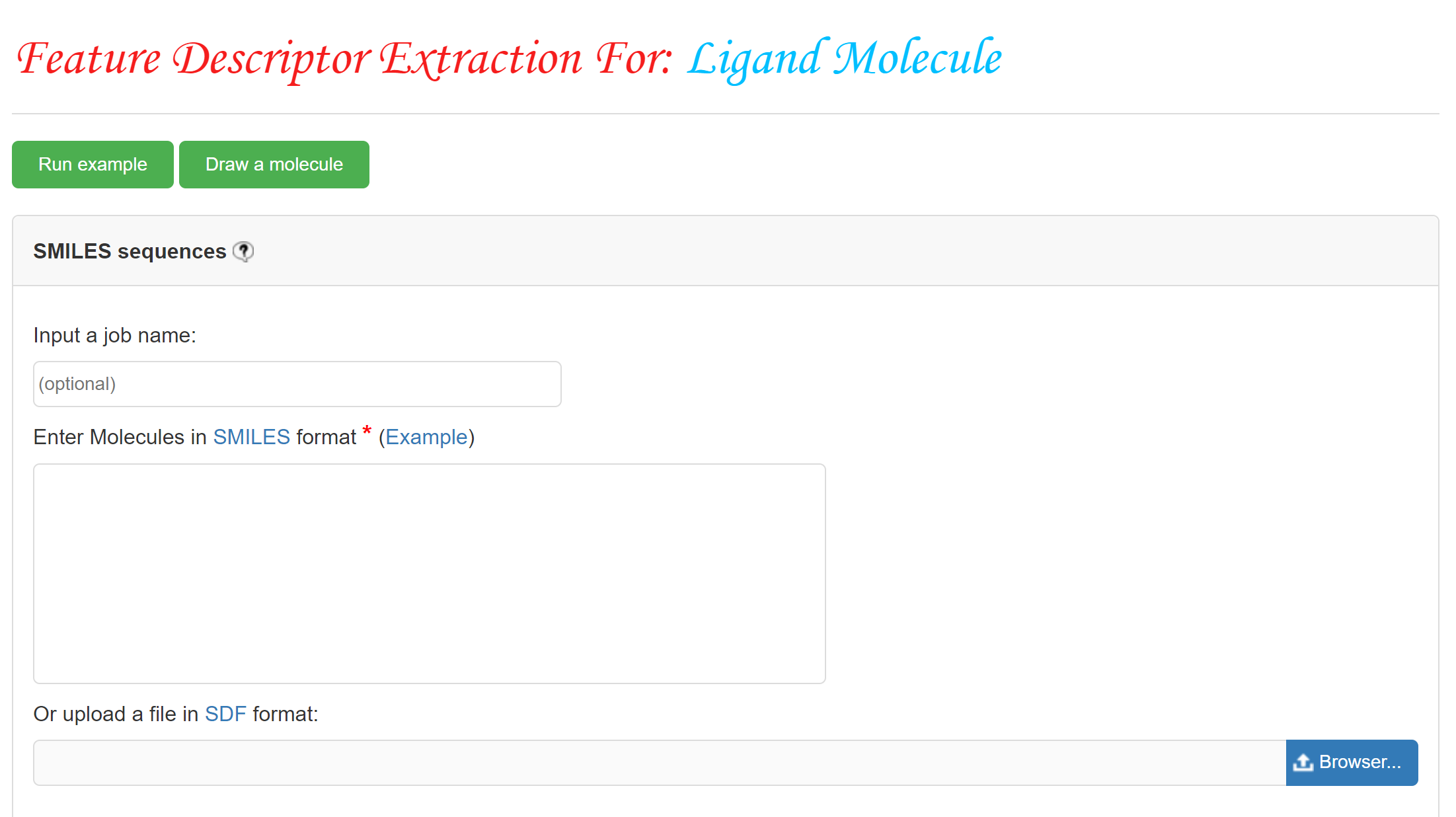


*Step 2. Paste ligands in SMILES format or upload a file with SDF format.*


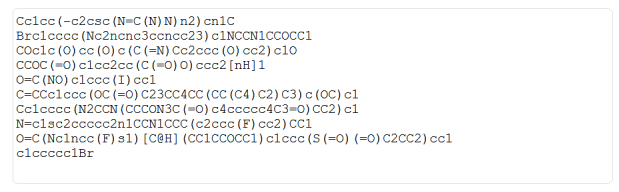


*Step 2: Select the descriptor type.*


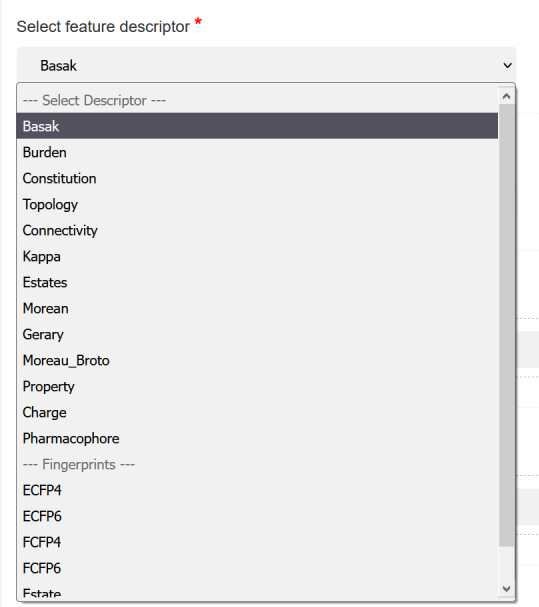


*Step 3: Select the clustering method.*


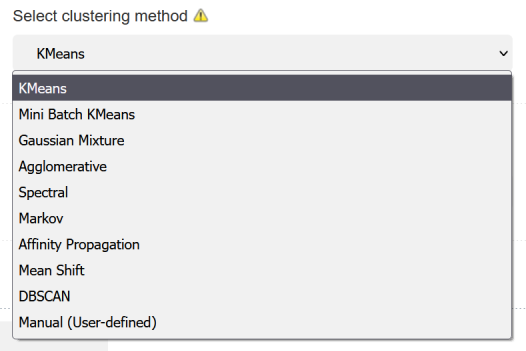


*Step 4: Select the feature normalization algorithm.*


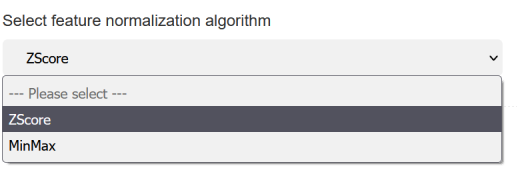


*Step 5: Select the dimension reduction methods.*


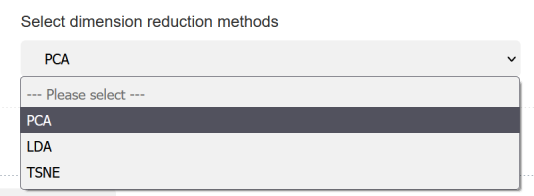


Finally, click the 'Submit' button to calculate the descriptors and run the feature analysis algorithms.

*Step 7. Wait for the results.*


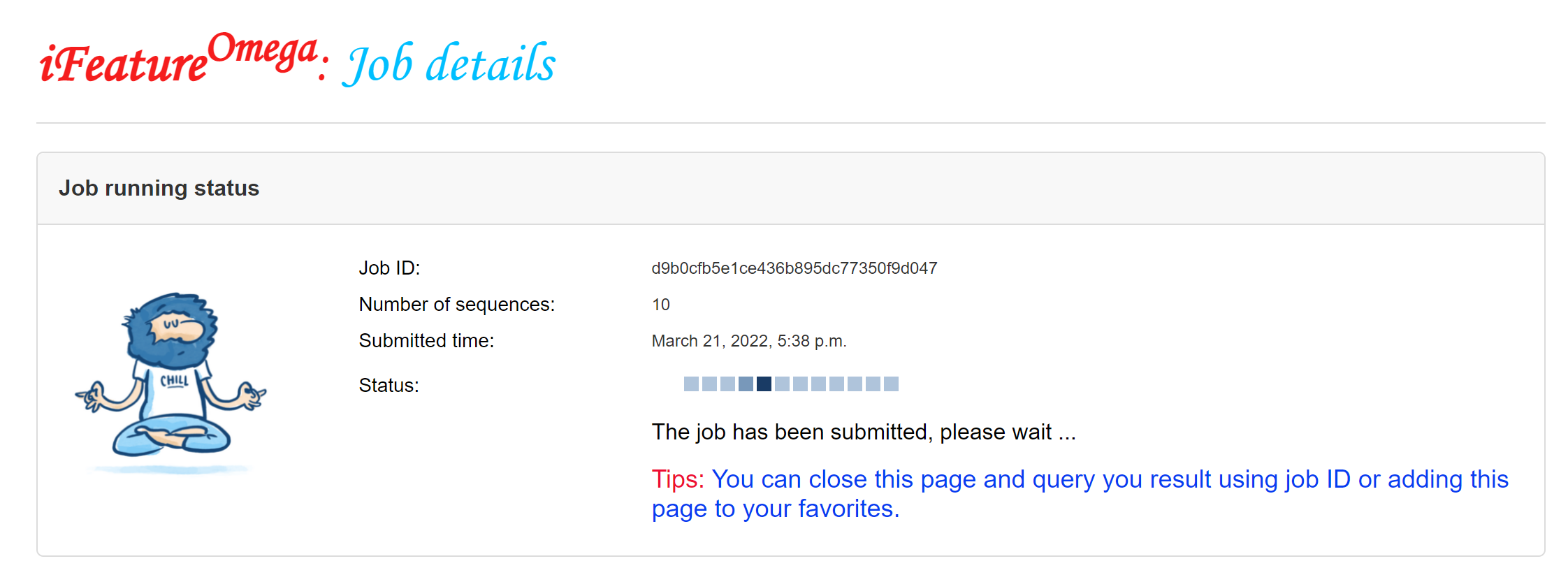


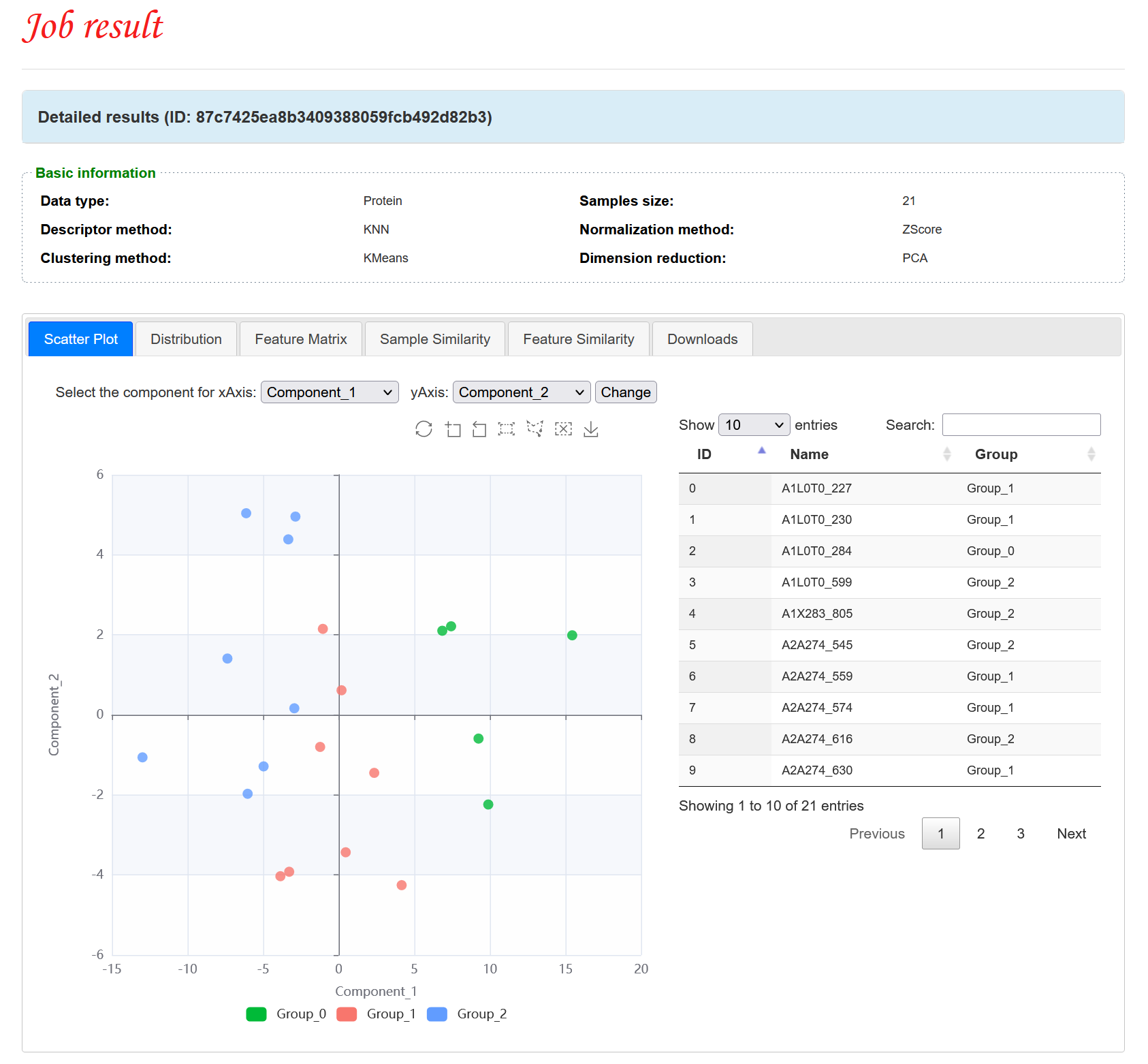


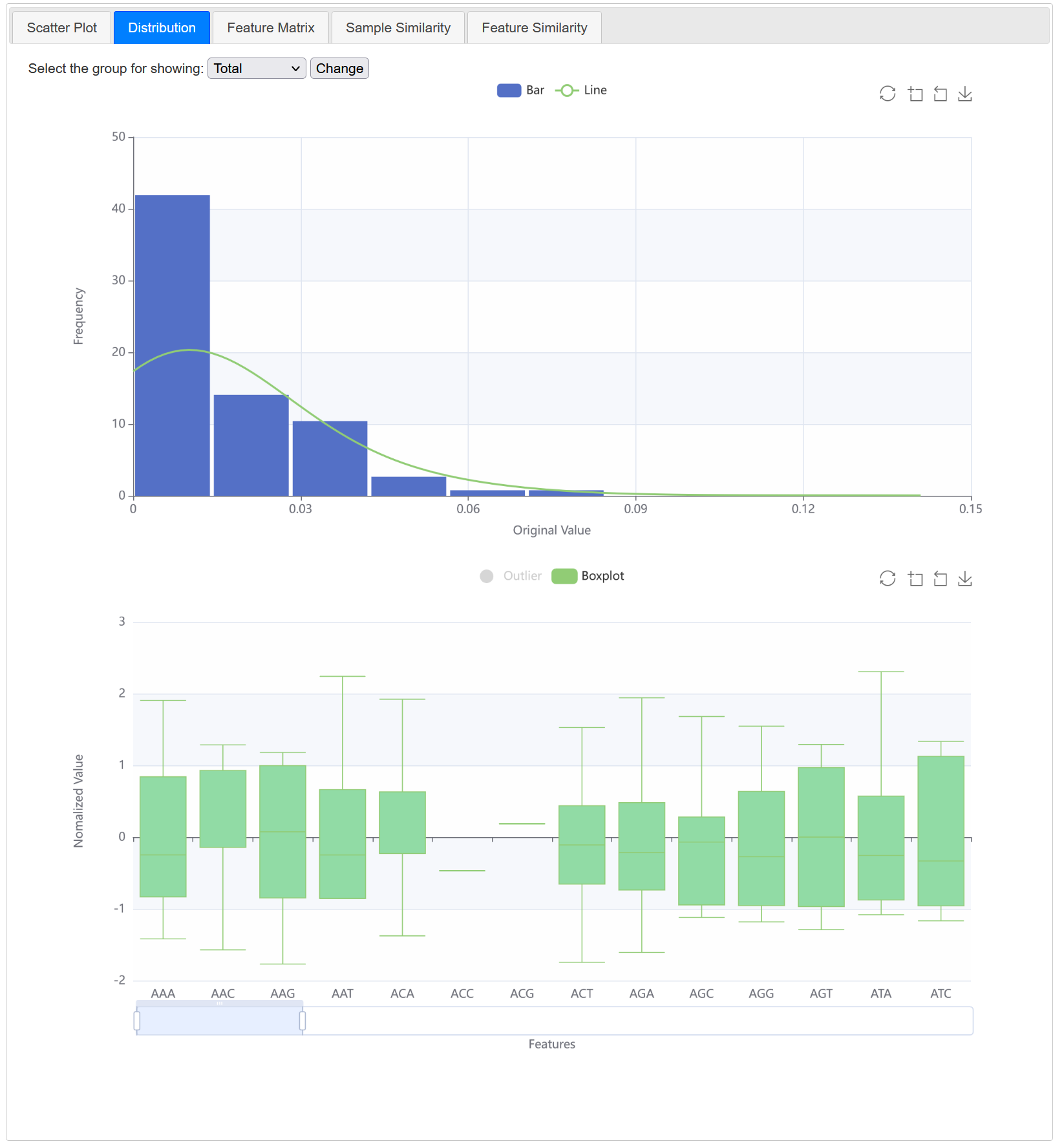


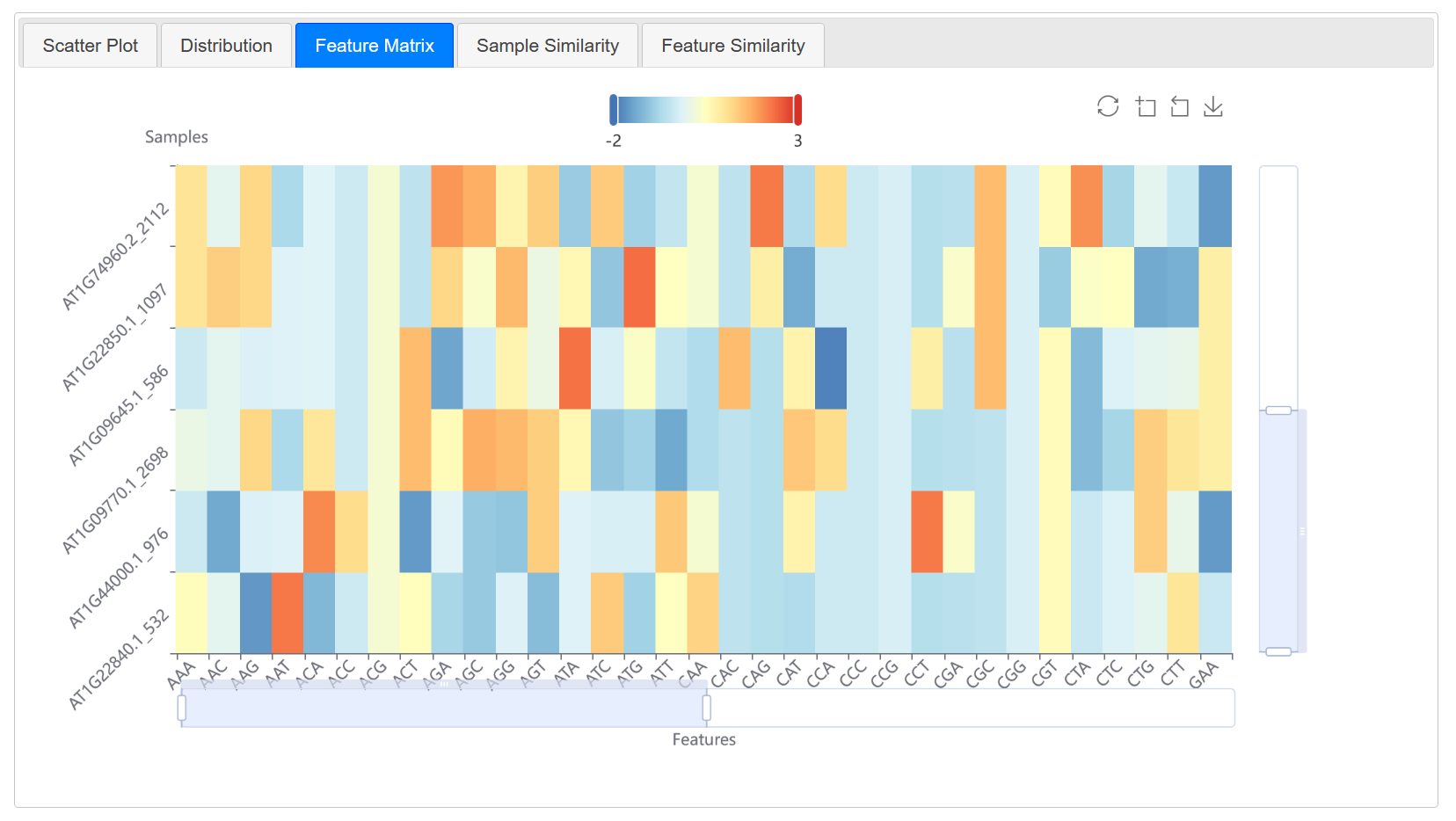


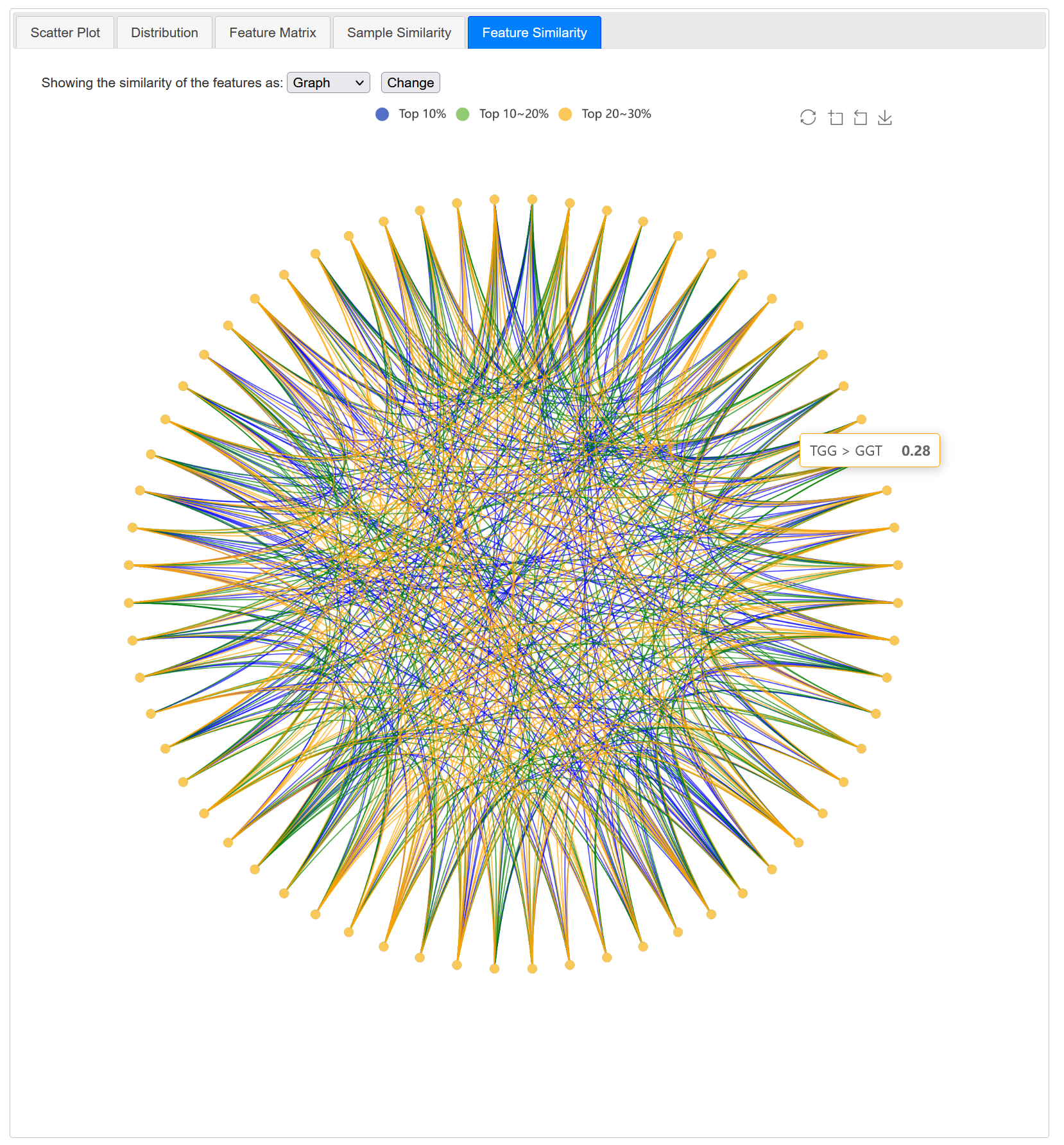


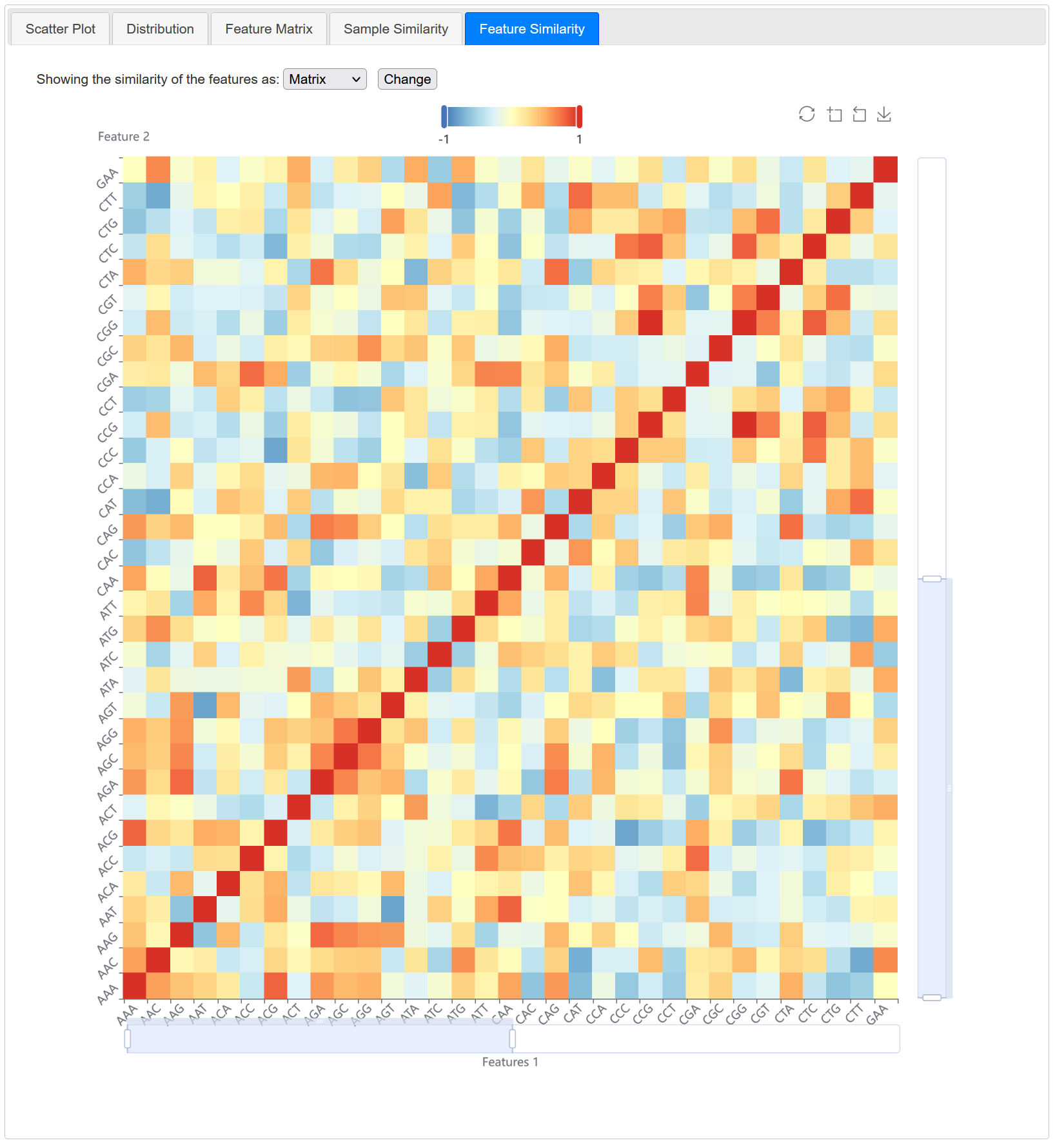


*Step 8: Query your results.*

You can query your jobs by inputting the job ID in the query form. An incorrect ID will return an error message.


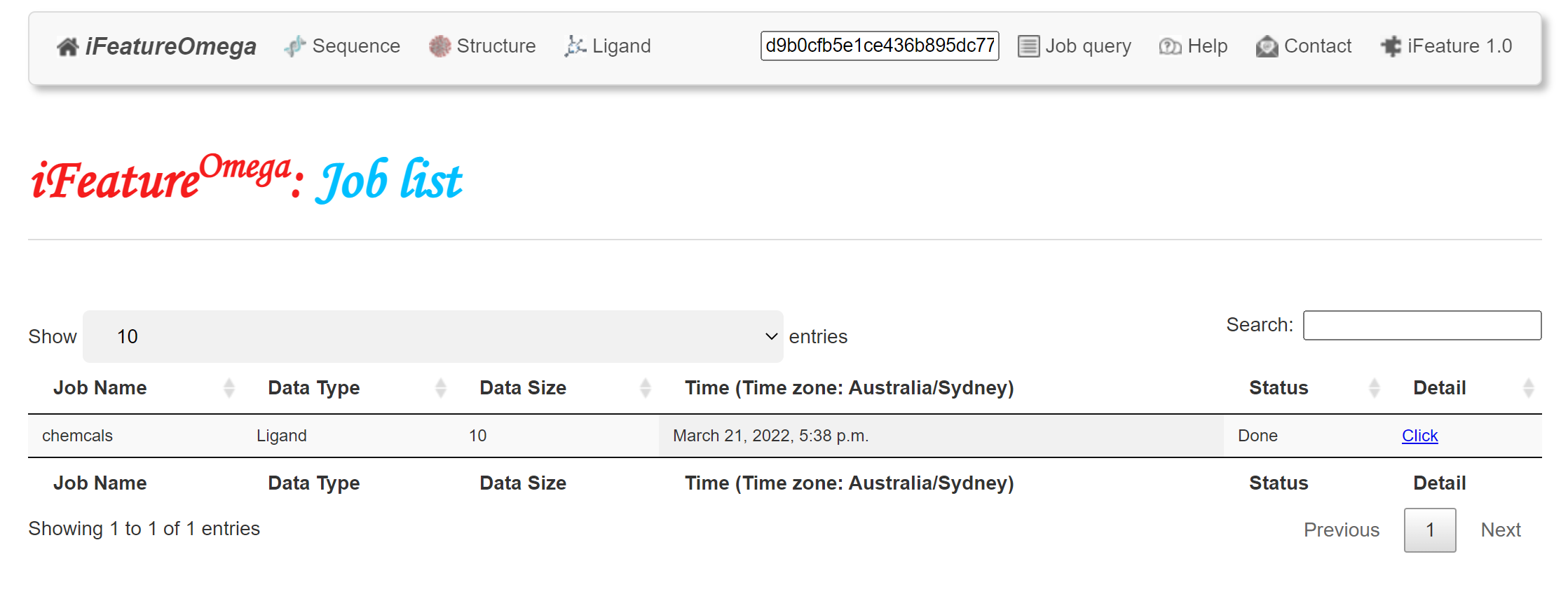


# 9. List of Integrated Feature Descriptors, Feature Analysis Plots in iFeatureOmega

**Table 2.** Feature descriptors calculated by *iFeatureOmega* for protein sequences

| **Feature group** | **Features (Abbreviation)** | **Reference** |
| --- | --- | --- |
| Amino acid composition | Amino acid composition (AAC) | (2) |
|  | Enhanced amino acid composition (EAAC) | (3,4) |
|  | Composition of *k*-spaced amino acid pairs (CKSAAP type 1 and type 2) | (5,6) |
|  | Kmer (dipeptides and tripeptides) composition (DPC type 1 and type 2 and TPC type 1 and type 2) | (2,7) |
|  | Dipeptide deviation from expected mean (DDE) | (7) |
|  | Composition (CTDC) | (8-12) |
|  | Transition (CTDT) | (8-12) |
|  | Distribution (CTDD) | (8-12) |
|  | Conjoint triad (CTriad) | (13) |
|  | Conjoint *k*-spaced Triad (KSCTriad) | (3,4) |
|  | Adaptive skip dipeptide composition (ASDC) | (14) |
|  | PseAAC of distance-pairs and reduced alphabet (DistancePair) | (15,16) |
| Grouped amino acid composition | Grouped amino acid composition (GAAC) | (3,4) |
|  | Enhanced grouped amino acid composition (EGAAC) | (3,4) |
|  | Composition of *k*-spaced amino acid group pairs (CKSAAGP type 1 and type 2) | (3,4) |
|  | Grouped dipeptide composition (GDPC type 1 and type 2) | (3,4) |
|  | Grouped tripeptide composition (GTPC type 1 and type 2) | (3,4) |
| Autocorrelation | Moran (Moran) | (17,18) |
|  | Geary (Geary) | (19) |
|  | Normalized Moreau-Broto (NMBroto) | (20) |
|  | Auto covariance (AC) | (21-23) |
|  | Cross covariance (CC) | (21-23) |
|  | Auto-cross covariance (ACC) | (21-23) |
| Quasi-sequence-order | Sequence-order-coupling number (SOCNumber) | (24-26) |
|  | Quasi-sequence-order descriptors (QSOrder) | (24-26) |
| Pseudo-amino acid composition | Pseudo-amino acid composition (PAAC) | (27,28) |
|  | BPAAC (APAAC) | (27,28) |
|  | Pseudo *K*-tuple reduced amino acids composition (PseKRAAC type 1 to type 16) | (29) |
| Residue representation | Binary - 20bit (binary) | (30,31) |
|  | Binary - 6bit (binary_6bit) | (16,32) |
|  | Binary - 5bit (binary_5bit_type 1 and type 2) | (16,33) |
|  | Binary - 3bit (binary_3bit_type 1 to type 7) | (14) |
|  | Learn from alignments (AESNN3) | (16,34) |
|  | Overlapping property features - 10 bit (OPF_10bit) | (14) |
|  | Overlapping property features - 7 bit (OPF_7bit type 1 to type 3) | (14) |
| Physicochemical property | AAindex (AAindex) | (35) |
| BLOSUM matrix | BLOSUM62 (BLOSUM62) | (36) |
| Z-Scales index | Z-Scales (ZSscale) | (37) |
| Similarity-based descriptor | K-nearest neighbor (KNN) | (38) |

**Table 3**. Feature descriptors calculated by *iFeatureOmega* for DNA and RNA sequences

| **Feature group** | **Features (Abbreviation)** | **Sequence type** | **Reference** |
| --- | --- | --- | --- |
| Nucleic acid composition | Nucleic acid composition (NAC) | DNA/RNA | (4) |
|  | Enhanced nucleic acid composition (ENAC) | DNA/RNA | (4) |
|  | *k*-spaced nucleic acid pairs (CKSNAP type 1 and type 2) | DNA/RNA | (4) |
|  | Basic kmer (Kmer type 1 and type 2) | DNA/RNA | (39) |
|  | Reverse complement kmer (RCKmer type 1 and type 2) | DNA | (40,41) |
|  | Accumulated nucleotide frequency (ANF) | DNA/RNA | (42) |
|  | Nucleotide chemical property (NCP) | DNA/RNA | (42) |
|  | The occurrence of kmers, allowing at most m mismatches (Mismatch) | DNA/RNA | (16) |
|  | The occurrences of kmers, allowing non-contiguous matches (Subsequence) | DNA/RNA | (16) |
|  | Adaptive skip dinucleotide composition (ASDC) | DNA/RNA | (14) |
|  | Local position-specific dinucleotide frequency (LPDF) | DNA/RNA | (43) |
|  | The Z curve parameters for frequencies of phase-specific mononucleotides (Z_curve_9bit) | DNA/RNA | (44) |
|  | The Z curve parameters for frequencies of phase-independent dinucleotides (Z_curve_12bit) | DNA/RNA | (44) |
|  | The Z curve parameters for frequencies of phase-specific dinucleotides (Z_curve_36bit) | DNA/RNA | (44) |
|  | The Z curve parameters for frequencies of phase-independent trinucleotides (Z_curve_48bit) | DNA/RNA | (44) |
|  | The Z curve parameters for frequencies of phase-specific trinucleotides (Z_curve_144bit) | DNA/RNA | (44) |
| Position-specific of n-nucleotides | Binary (binary) | DNA/RNA | (30,31) |
|  | Dinucleotide binary encoding (DBE) | DNA/RNA | (43) |
|  | Position-specific of two nucleotides (PS2) | DNA/RNA | (16,45) |
|  | Position-specific of three nucleotides (PS3) | DNA/RNA | (16,45) |
|  | Position-specific of four nucleotides (PS4) | DNA/RNA | (16,45) |
|  | Position-specific trinucleotide propensity based on single-strand (PSTNPss) | DNA/RNA | (46,47) |
|  | Position-specific trinucleotide propensity based on double-strand (PSTNPds) | DNA | (46,47) |
| Electron-ion interaction pseudopotentials | Electron-ion interaction pseudopotentials value (EIIP) | DNA | (48,49) |
|  | Electron-ion interaction pseudopotentials of trinucleotide (PseEIIP) | DNA | (48,49) |
| Autocorrelation and cross-covariance | Dinucleotide-based auto covariance (DAC) | DNA/RNA | (21-23) |
|  | Dinucleotide-based cross covariance (DCC) | DNA/RNA | (21-23) |
|  | Dinucleotide-based auto-cross covariance (DACC) | DNA/RNA | (21-23) |
|  | Trinucleotide-based auto covariance (TAC) | DNA | (21) |
|  | Trinucleotide-based cross covariance (TCC) | DNA | (21) |
|  | Trinucleotide-based auto-cross covariance (TACC) | DNA | (21) |
|  | Moran (Moran) | DNA/RNA | (17,18) |
|  | Geary (Geary) | DNA/RNA | (19) |
|  | Normalized Moreau-Broto (NMBroto) | DNA/RNA | (20) |
| Physicochemical property | Dinucleotide physicochemical properties (DPCP type 1 and type 2) | DNA/RNA | (50) |
|  | Trinucleotide physicochemical properties (TPCP type 1 and type 2) | DNA | (50) |
| Mutual information | Multivariate mutual information (MMI) | DNA/RNA | (51) |
| Pseudo nucleic acid composition | Pseudo dinucleotide composition (PseDNC) | DNA/RNA | (21,52) |
|  | Pseudo *k*-tupler composition (PseKNC) | DNA/RNA | (21,52) |
|  | Parallel correlation pseudo dinucleotide composition (PCPseDNC) | DNA/RNA | (21,52) |
|  | Parallel correlation pseudo trinucleotide composition (PCPseTNC) | DNA | (21,52) |
|  | Series correlation pseudo dinucleotide composition (SCPseDNC) | DNA/RNA | (21,52) |
|  | Series correlation pseudo trinucleotide composition (SCPseTNC) | DNA | (21,52) |
| Similarity-based descriptor | *K*-nearest neighbor (KNN) | DNA/RNA | (51) |

**Table 4.** Feature descriptors calculated by *iFeatureOmega* for ligands

| **Feature group** | **Features (Abbreviation)** | **Reference** |
| --- | --- | --- |
| Constitution | Molecular constitutional descriptors | (53,54) |
| Topology | Topological descriptors | (53,54) |
|  | Molecular connectivity indices | (53,54) |
| E-State | E‑state descriptors | (53,54) |
| Basak | Basak descriptors | (53,54) |
| Burden | Burden descriptors | (53,54) |
| Kappa | Kappa shape descriptors | (53,54) |
| Autocorrelation | Moreau–Broto autocorrelation | (53,54) |
|  | Moran autocorrelation | (53,54) |
|  | Geary autocorrelation | (53,54) |
| Charge | Charge descriptors | (53,54) |
| Property | Molecular property | (53,54) |
| Pharmacophore | Potential pharmacophore point descriptors | (53,54) |
| MOE-type | MOE‑type descriptors | (53,54) |
| Fingerprints | MACCS fingerprints | (53,54) |
|  | Morgan fingerprints | (53,54) |
|  | E-state fingerprints | (53,54) |

**Table 5**. Feature descriptors calculated by *iFeatureOmega* for protein structures.

| **Descriptor group** | **Descriptor (Abbreviation)** |
| --- | --- |
| Amino acids composition | Amino acids content type 1 (AAC_type1) |
|  | Amino acids" content type 2 (AAC_type2) |
| Grouped amino acids composition | Grouped amino acids content type 1 (GAAC_type1) |
|  | Grouped amino acids content type 2 (GAAC_type2) |
| Secondary structure | Secondary structure elements (3) type 1 (SS3_type1) |
|  | Secondary structure elements (3) type 2 (SS3_type2) |
|  | Secondary structure elements (8) type 1 (SS8_type1) |
|  | Secondary structure elements (8) type 2 (SS8_type2) |
| Half sphere exposure | Half sphere exposure α (HSE_CA) |
|  | Half sphere exposure β (HSE_CB) |
| Residue depth | Residue depth (Residue depth) |
| Atom composition | Atom content type 1 (AC_type1) |
|  | Atom content type 2 (AC_type2) |
| Network-based index | Network-based index |

**Table 6.** The feature analysis approaches provided in iFeatureOmega

| **Method** | **Algorithm (Abbreviation)** | **Reference** |
| --- | --- | --- |
| Clustering | *k*-means (kmeans) | (55,56) |
|  | Mini-Batch *K*-means (MiniBatchKMeans) | (55,56) |
|  | Gaussian mixture (GM) | (55,56) |
|  | Agglomerative (Agglomerative) | (57) |
|  | Spectral (Spectral) | (58) |
|  | Markov clustering (MCL) | (59) |
|  | Hierarchical clustering (hcluster) | (55,60) |
|  | Affinity propagation clustering (APC) | (61) |
|  | Mean shift (meanshift) | (62) |
|  | DBSCAN (dbscan) | (63) |
| Dimensionality reduction | Principal component analysis (PCA) | (64) |
|  | Latent dirichlet allocation (LDA) | (65) |
|  | *t*-distributed stochastic neighbor embedding (*t*_SNE) | (66) |
| Feature normalization | ZScore (ZScore) | (4) |
|  | MinMax (MinMax) | (4) |

**Table 7**. Graphical display options in *iFeatureOmega*

| **Category** | **Type** | **Purpose** |
| --- | --- | --- |
| Feature descriptor visualization | Histogram | Display data distribution |
|  | Kernel density plot | Display data distribution |
|  | Heatmap | Display data distribution |
|  | Boxplot | Display data distribution |
|  | Line chart | Display data distribution |
|  | Scatter plot | Display clustering and dimensionality reduction result |
|  | Circular plot | Display the correlation of samples or descriptors |
| Protein structure visualization |  | Display the 3-D protein structure |
| Ligand structure visualization |  | Display chemical structure |

**10. Descriptions of feature descriptors for nucleotide sequences**

Let us assume that a nucleotide sequence with *L* residues can be generally represented as {*R_1_*, *R_2_*, …, *R_L_*}, where *R_i_* represents the base at the *i-th* position in the sequence. The following commonly used feature descriptors can be described as follows:

**Kmer type 1**

For kmer type 1 descriptor, the DNA or RNA sequences are represented as the occurrence frequencies of *k* neighboring nucleic acids (39), which has been successfully applied to human gene regulatory sequence prediction (40) and enhancer identification (39). The Kmer type 1(*k*=3) descriptor can be calculated as:

$f\left( t \right)=\frac{N(t)}{N}$*, t ∈ {AAA, AAC, AAG, …, TTT},*

where *N(t)* is the number of kmer type *t*, while *N* is the length of a nucleotide sequence. The Kmer descriptor has been successfully applied to lncRNA prediction (67,68).

*Parameter(s)*:

- *Kmer size: The number of neighboring nucleic acids, default: 3.*

**Kmer type 2**

For the kmer type 2 descriptor, the DNA or RNA sequences are represented as the raw count of *k* neighboring nucleic acids (39).

*Parameter(s)*:

- *Kmer size: The number of neighboring nucleic acids, default: 3.*

**RCKmer type 1 and type 2 (Reverse complement kmer type 1 and type 2)**

The reverse complement kmer type 1 and type 2 (40,41) is a variant of kmer descriptor, in which the kmers are not expected to be strand-specific. For instance, for a DNA sequence, there are 16 types of 2-mers (i.e. 'AA', 'AC', 'AG', 'AT', 'CA', 'CC', 'CG', 'CT', 'GA', 'GC', 'GG', 'GT', 'TA', 'TC', 'TG', 'TT'), ‘TT’ is reverse complement with ‘AA’. After removing the reverse compliment kmers, there are only 10 distinct kmers in the reverse complement kmer approach ('AA', 'AC', 'AG', 'AT', 'CA', 'CC', 'CG', 'GA', 'GC', 'TA'). It has been used to predict the *in vivo* signature of human gene regulatory sequences (40) and human nucleosome occupancy (41). RCKmer type 1 calculates the reverse complement kmer frequency, while RCKmer type 2 calculates the reverse complement kmer raw count.

*Parameter(s)*:

- *Kmer size: The number of neighboring nucleic acids, default: 3.*

**Mismatch (The occurrence of kmers, allowing at most m mismatches)**

The mismatch profile also calculates the occurrences of kmers (16), but allows max *m* inexact matching (*m* < *k*). There are two parameters for this descriptor, *k* neighboring nucleic acids and *m* inexact matching. The mismatch descriptor is defined as:

$$f_{k,m}=(\sum_{j=0}^{m} c_{1,j},\sum_{j=0}^{m} c_{2,j},\ldots,\sum_{j=0}^{m} c_{4^{k},j},),$$

where *c_i,j_* represents the occurrences of *i*-th kmer type with *j* mismatches, *i* = 1, 2, 3, …, 4*^k^*; *j* = 0, 1, 2, …, *m*. The mismatch descriptor has been successfully applied to protein classification prediction (69), B-cell epitopes identification (70) and transposon-derived piRNA prediction (71).

*Parameter(s)*:

- *Kmer size: The number of k neighboring nucleic acids, default: 3.*
- *Mismatch value: the nucleotide number of inexact matching (< kmer size), default: 1.*

**Subsequence (The occurrences of kmers, allowing non-contiguous matches)**

The subsequence descriptor allows non-contiguous matching (16). For example, the 3-mer “AAC” in the sequence “AACTACG”. By exact and non-contiguous matching, we can obtain AAC, AA-C, A-AC, A-AC (“-” means the gap in non-contiguous matching). AAC is the exact form of “AAC”, and AA-C, A-AC, A-AC are non-contiguous forms of “AAC”. The occurrences of non-contiguous forms are penalized with their length *l* and the factor *δ* (0 ≤ *δ* ≤ 1), defined as *δ^l^*. Therefore, the occurrence of “AAC” in above example is 1 + 2*δ*^6^ + *δ*^5^. The subsequence descriptor has been successfully applied to B-cell epitopes identification (70), transposon-derived piRNA prediction (71).

*Parameter(s)*:

- *Kmer size: the number of k neighboring nucleic acids, default: 3.*
- *Delta value: the penalty factor, range from 0 to 1, default: 0.*

**NAC (Nucleic Acid Composition)**

The Nucleic Acid Composition (NAC) encoding calculates the frequency of each nucleic acid type in a nucleotide sequence. The frequencies of all 4 natural nucleic acids (i.e. “ACGT or U”) can be calculated as:

$f\left( t \right)=\frac{N(t)}{N}$*, t* ∈ {A, C, G, T(U)},

where *N*(*t*) is the number of nucleic acid type *t*, while *N* is the length of a nucleotide sequence.

**Di-Nucleotide Composition (DNC)**

The Di-Nucleotide Composition gives 16 descriptors. It is defined as:

$$D\left( r,s \right)=\frac{N_{rs}}{N-1}, r,s \in\{A,C,G,T(U)\}$$

where *N_rs_* is the number of di-nucleotide represented by nucleic acid types *r* and *s*.

**Tri-Nucleotide Composition (TNC)**

The Tri-Nucleotide Composition gives 64 descriptors. It is defined as:

$$D\left( r,s,t \right)=\frac{N_{rst}}{N-2}, r,s,t \in\{A,C,G,T(U)\}$$

where *N_rst_* is the number of tri-nucleotide represented by nucleic acid types *r, s* and *t*.

**ANF (Accumulated nucleotide frequency)**

The Accumulated Nucleotide Frequency (ANF) encoding (42) include the nucleotide frequency information and the distribution of each nucleotide in the RNA sequence, the density *d_i_* of any nucleotide *s_i_* at position *i* in RNA sequence by the following formula:

$$d_{i}=\frac{1}{|s_{i}|}\sum_{j=1}^{l} f\left( s_{i} \right), f\left( q \right)= \left\{ \begin{aligned} 1 if s_{i}=q \\ 0 other case \end{aligned} \right.,$$

where *l* is the sequence length, |*S_i_*| is the length of the *i*-th prefix string {*s_1_*, *s_2_*, …, *s_i_*} in the sequence, *q* ∈ {A, C, G or U}. Suppose an example sequence “UCGUUCAUGG”. The density of ‘U’ is 1 (1/1), 0.5 (2/4), 0.6 (3/5), 0.5 (4/8) at positions 1, 4, 5, and 8, respectively. The density of ‘C’ is 0.5 (1/2), 0.33 (2/6) at positions 2 and 6, respectively. The density of ‘G’ is 0.33 (1/3), 0.22 (2/9), 0.3 (3/10) at positions 3, 9, and 10, respectively. The density of ‘A’ is 0.14 (1/7) at position 7.

By integrating both the nucleotide chemical property and accumulated nucleotide information, the sample sequence “UCGUUCAUGG” can be represented by {(0, 0, 1, 1), (0, 1, 0, 0.5), (1, 0, 0, 0.33), (0, 0, 1, 0.5), (0, 0, 1, 0.6), (0, 1, 0, 0.33), (1, 1, 1, 0.14), (0, 0, 1, 0.5), (1, 0, 0, 0.22), (1, 0, 0, 0.3)}. By doing so, not only the chemical property was considered, but also the long-range sequence order information was incorporated. Therefore, the samples in the benchmark dataset were encoded in terms of both nucleotide chemical property and nucleotide densities. The ANF descriptor has been successfully applied to N(6)-methyldenosine site prediction (42).

**ENAC (Enhanced nucleic acid composition)**

The Enhanced Nucleic Acid Composition (ENAC) calculates the NAC based on the sequence window of fixed length (the default value is 5) that continuously slides from the 5’ to 3’ terminus of each nucleotide sequence and can be usually applied to encode the nucleotide sequence with an equal length, which has been successfully applied to N(6)-methyldenosine site prediction (72).

*Parameter(s)*:

- *Sliding window size: the length of the sliding window, default: 5.*

**Binary (also termed one-hot)**

In the Binary encoding, each amino acid is represented by a 4-dimensional binary vector, e.g. A is encoded by (1000), C is encoded by (0100), G is encoded by (0010) and T(U) is encoded by (0001), respectively. This encoding scheme is often used to encode nucleotide sequence with an equal length. The binary descriptor has been successfully applied to N(6)-methyldenosine sites prediction (72), RNA pseudouridylation sites prediction (73), DNA- and RNA-binding proteins prediction (74) and noncoding variants effect prediction (75).

**PS2 (Position-specific of two nucleotides)**

There are 4 × 4 = 16 pairs of adjacent pairwise nucleotides, such as AA/AT/AG …, thus a single variable representing one such pair gets one-hot (i.e. binary) encoded into 16 binary variables (16). For example, e.g. AA is encoded by (1000000000000000), AC is encoded by (0100000000000000) …, and the sequence AAC is encoded as (10000000000000000100000000000000). PS2 has been successfully applied to off-target effects of CRISPR-Cas9 prediction (45). Both PS3 and PS4 are encoded for three adjacent nucleotides (4 × 4 × 4 = 64) and four adjacent nucleotides (4 × 4 × 4 × 4 = 256) in a similar way.

**CKSNAP type 1 (Composition of *k*-spaced nucleic acid pair type 1)**

The CKSNAP type 1 feature encoding calculates the frequency of nucleic acid pairs separated by any *k* nucleic acid (*k* = 0, 1, 2, … , 5) (4). Taking *k* = 0 as an example, there are 16 0-spaced nucleic acid pairs (i.e. 'AA', 'AC', 'AG', 'AT', 'CA', 'CC', 'CG', 'CT', 'GA', 'GC', 'GG', 'GT', 'TA', 'TC', 'TG', 'TT'). Then, a feature vector can be defined as:

$$(\frac{N_{AA}}{N_{total}}, \frac{N_{Ac}}{N_{total}}, \frac{N_{AG}}{N_{total}}, \ldots, \frac{N_{TT}}{N_{total}},)_{16}.$$

The value of each descriptor denotes the composition of the corresponding nucleic acid pair in the nucleotide sequence. For instance, if the nucleic acid pair AA appears *m* times in the nucleotide sequence, the composition of the nucleic acid pair AA is equal to *m* divided by the total number of *0*-spaced nucleic acid pairs (*N_total_*) in the nucleotide sequence. For *k* = 0, 1, 2, 3, 4 and 5, the value of *N_total_* is *P* – 1, *P* – 2, *P* – 3, *P* – 4, *P* – 5 and *P* – 6 for a nucleotide sequence of length *P*, respectively.

*Parameter(s)*:

- *Gap value:* *the length of k-spaced nucleic acid, default is 5.*

**CKSNAP type 2 (Composition of *k*-spaced nucleic acid pairs type 2)**

The CKSNAP type 2 feature encoding calculates the raw counts of nucleic acid pairs separated by any *k* nucleic acid (*k* = 0, 1, 2, … , 5) (4).

**NCP (Nucleotide chemical property)**

There are four different kinds of nucleotides in RNA, i.e., adenine (A), guanine (G), cytosine (C) and uracil (U). Each nucleotide has different chemical structure and chemical binding. The four kinds of nucleotides can be classified into three different groups in terms of these chemical properties (**Table 8**).

**Table 8.** Chemical structure of each nucleotide (42).

| Chemical property | Class | Nucleotides |
| --- | --- | --- |
| Ring Structure | Purine | A, G |
|  | Pyrimidine | C, U |
| Functional Group | Amino | A, C |
|  | Keto | G, U |
| Hydrogen Bond | Strong | C, G |
|  | Weak | A, U |

Based on chemical properties, A can be represented by coordinates (1, 1, 1), C can be represented by coordinates (0, 1, 0), G can be represented by coordinates (1, 0, 0), U can be represented by coordinates (0, 0, 1). The NCP descriptor has been successfully applied to N(6)-methyldenosine site prediction (42).

**EIIP (Electron-ion interaction pseudopotentials)**

Dragutin (49) came up with electron-ion interaction pseudopotentials (EIIP) value of nucleotides A, G, C, T (A: 0.1260, C: 0.1340, G: 0.0806, T:0.1335). The EIIP directly use the EIIP value represent the nucleotide in the DNA sequence. Therefore, the dimension of the EIIP descriptor is the length of the DNA sequence. The EIIP descriptor has been successfully applied to DNA N4-methylcytosine site prediction (76) and subcellular location prediction (77).

**PseEIIP (Electron-ion interaction pseudopotentials of trinucleotide)**

In these encoding, let EIIP_A_, EIIP_T_, EIIP_G_, and EIIP_C_ denote the EIIP values of nucleotides A, T, G and C, respectively. Then, the mean EIIP value of trinucleotides in each sample to construct feature vector, which can be formulated as:

$$V=\left[ {EIIP}_{AAA}\cdot f_{AAA},{EIIP}_{AAC}\cdot f_{AAC}, \ldots,{EIIP}_{TTT}\cdot f_{TTT} \right],$$

where *f_xyz_* is the normalized frequency of the *i*-th trinucleotide, *EIIP_xyz_* = *EIIP_x_*+ *EIIP_y_* + *EIIP_z_* expresses the *EIIP* value of one trinucleotide and X, Y, Z∈ [A, C, G, T]. Obviously, the dimension of vector *V* is 64. The EIIP descriptor has been successfully applied to 5-Methylcytosine prediction (78) and non-coding RNA promoter identification (79).

**ASDC (Adaptive skip dinucleotide composition)**

The adaptive skip dipeptide composition is a modified dinucleotide composition, which sufficiently considers the correlation information present not only between adjacent residues but also between intervening residues (80). For given a sequence, the feature vector for ASDC is represented by:

$$ASDC=\left( f_{v1},f_{v1},\ldots,f_{v16} \right),$$

where *f_vi_* is calculated by

$$f_{vi}=\frac{\sum_{g=1}^{L-1} O_{i}^{g}}{\sum_{i=1}^{16} \sum_{g=1}^{L-1} O_{i}^{g}},$$

where *f_vi_* denotes the occurrence frequency of all possible dinucleotide with ≤ *L*-1 intervening nucleotides. The ASDC descriptor has been successfully applied to anti-cancer peptide (80) and cell-penetrating peptide predictions (81).

**DBE (Dinucleotide binary encoding)**

The dinucleotide binary encoding descriptor encapsulates the positional information of the dinucleotide at each position in the sequence. There are a total of 16 possible dinucleotides. In this descriptor, each dinucleotide can be encoded into a 4-dimensional 0/1 vector. For example, AA is encoded as (0,0,0,0); AT is encoded as (0,0,0,1); AC is encoded as (0,0,1,0); and so forth, GG is encoded as (1,1,1,1). Therefore, using the dinucleotide binary encoding, we can yield a 160 (=40×4)-dimensional 0/1 vector for the given sequence. The DBE descriptor has been successfully applied to N6-Methyladenosine site prediction (43).

**LPDF (Local position-specific dinucleotide frequency)**

The local position-specific dinucleotide frequency descriptor can be denoted as (*f_2_*, *f_3_*, …, *f_l_*), where *f_i_* is calculated as follows:

$$f=\frac{1}{\left| N_{i} \right|}C\left( X_{i-1}X_{i} \right), 2\leq i\leq l,$$

where *l* is the length of the given sequence, |*N_i_*| is the length of the *i*-th prefix string {*X_1_X_2_…X_i_*} in the sequence, and C (*X_i-1_X_i_*) is the occurrence number of the dinucleotide *X_i-1_X_i_* in position *i* of the *i*-th prefix string. The LPDF descriptor has been successfully applied to N6-Methyladenosine site prediction (43).

**DPCP (Dinucleotide physicochemical properties)**

The dinucleotide physicochemical properties descriptor can be devised as:

$$V=\left[ {DPCP}_{AA}\times f_{AA}, {DPCP}_{AC}\times f_{Ac}, \ldots,{DPCP}_{TT}\times f_{TT} \right],$$

where *DPCP_i_* is one of the *i*-th physicochemical properties of a dinucleotide. The 148 physicochemical properties for DNA dinucleotide and 22 physicochemical properties for RNA dinucleotide are listed in **Tables 9** and **10**, respectively. The DPCP descriptor has been successfully applied to DNA N4-Methylcytosine sites prediction (50).

*Parameter(s)*:

- *Di-DNA physicochemical indices:* *the names of used physicochemical dinucleotides indices. (For DNA sequences)*
- *Di-RNA physicochemical indices:* *the names of used physicochemical dinucleotides indices. (For RNA sequences)*

**Table 9.** The names of the 148 physicochemical dinucleotides indices for DNA.

| Base stacking | Protein induced deformability | B-DNA twist | Propeller twist | Duplex stability:(freeenergy) |
| --- | --- | --- | --- | --- |
| Duplex tability(disruptenergy) | Protein DNA twist | Stabilising energy of Z-DNA | Aida_BA_transition | Breslauer_dS |
| Electron_interaction | Hartman_trans_free_energy | Lisser_BZ_transition | Polar_interaction | SantaLucia_dG |
| Sarai_flexibility | Stability | Stacking_energy | Sugimoto_dS | Watson-Crick_interaction |
| Twist | Shift | Slide | Rise | Twist stiffness |
| Tilt stiffness | Shift_rise | Twist_shift | Enthalpy1 | Twist_twist |
| Shift2 | Tilt3 | Tilt1 | Slide (DNA-protein complex) 1 | Tilt_shift |
| Twist_tilt | Roll_rise | Stacking energy | Stacking energy1 | Propeller Twist |
| Roll11 | Rise (DNA-protein complex) | Roll2 | Roll3 | Roll1 |
| Slide_slide | Enthalpy | Shift_shift | Flexibility_slide | Minor Groove Distance |
| Rise (DNA-protein complex)1 | Roll (DNA-protein complex)1 | Entropy | Cytosine content | Major Groove Distance |
| Twist (DNA-protein complex) | Purine (AG) content | Tilt_slide | Major Groove Width | Major Groove Depth |
| Free energy6 | Free energy7 | Free energy4 | Free energy3 | Free energy1 |
| Twist_roll | Flexibility_shift | Shift (DNA-protein complex) 1 | Thymine content | Tip |
| Keto (GT) content | Roll stiffness | Entropy1 | Roll_slide | Slide (DNA-protein complex) |
| Twist2 | Twist5 | Twist4 | Tilt (DNA-protein complex)1 | Twist_slide |
| Minor Groove Depth | Persistance Length | Rise3 | Shift stiffness | Slide3 |
| Slide2 | Slide1 | Rise1 | Rise stiffness | Mobility to bend towards minor groove |
| Dinucleotide GC Content | A-philicity | Wedge | DNA denaturation | Bending stiffness |
| Free energy5 | Breslauer_dG | Breslauer_dH | Shift (DNA-protein complex) | Helix-Coil_transition |
| Ivanov_BA_transition | Slide_rise | SantaLucia_dH | SantaLucia_dS | Minor Groove Width |
| Sugimoto_dG | Sugimoto_dH | Twist1 | Tilt | Roll |
| Twist7 | Clash Strength | Roll_roll | Roll (DNA-protein complex) | Adenine content |
| Direction | Probability contacting nucleosome core | Roll_shift | Shift_slide | Shift1 |
| Tilt4 | Tilt2 | Free energy8 | Twist (DNA-protein complex)1 | Tilt_rise |
| Free energy2 | Stacking energy2 | Stacking energy3 | Rise_rise | Tilt_tilt |
| Roll4 | Tilt_roll | Minor Groove Size | GC content | Inclination |
| Slide stiffness | Melting Temperature1 | Twist3 | Tilt (DNA-protein complex) | Guanine content |
| Twist6 | Major Groove Size | Twist_rise | Rise2 | Melting Temperature |
| Free energy | Mobility to bend towards major groove | Bend |  |  |

**Table10.** The names of the 22 physicochemical dinucleotides indices for RNA.

| Shift (RNA) | Hydrophilicity (RNA) | Hydrophilicity (RNA) | GC content | Purine (AG) content |
| --- | --- | --- | --- | --- |
| Keto (GT) content | Adenine content | Guanine content | Cytosine content | Thymine content |
| Slide (RNA) | Rise (RNA) | Tilt (RNA) | Roll (RNA) | Twist (RNA) |
| Stacking energy (RNA) | Enthalpy (RNA) | Entropy (RNA) | Free energy (RNA) | Free energy (RNA) |
| Enthalpy (RNA) | Entropy (RNA) |  |  |  |

**DPCP type2 (Dinucleotide physicochemical properties)**

For DPCP type2 descriptor, it can be encoded into the following matrix:

$$V=\left[ \begin{matrix} {PC}^{1}(N_{1}N_{2}) & \ldots& {PC}^{1}(N_{L-1}N_{L}) \\ \ldots& \ldots& \ldots\\ {PC}^{j}(N_{1}N_{2}) & \ldots& {PC}^{j}(N_{L-1}N_{L}) \end{matrix} \right],$$

where *PC_j_*(*N_i_*, *N_i+1_*) is the *j*-th physicochemical dinucleotides value of the dinucleotide *N_i_N_i+1_*. *L* is the length of sequence. The 148 physicochemical properties for DNA dinucleotide and 22 physicochemical properties for RNA dinucleotide are listed in **Tables 9** and **10**, respectively. The DPCP type2 descriptor has been successfully applied to DNA N4-Methylcytosine sites prediction (51).

*Parameter(s)*:

- *Di-DNA physicochemical indices:* *the names of used physicochemical dinucleotides indices. (For DNA sequences)*
- *Di-RNA physicochemical indices:* *the names of used physicochemical dinucleotides indices. (For RNA sequences)*

**TPCP (Trinucleotide physicochemical properties**)

The trinucleotide physicochemical properties descriptor can be devised as:

$$V=\left[ {TPCP}_{AAA}\times f_{AAA}, {TPCP}_{AAC}\times f_{AAC}, \ldots,{DPCP}_{TTT}\times f_{TTT} \right],$$

where *TPCP_i_* is one of the *i*-th physicochemical properties of a trinucleotide listed in **Table 11**, and *f_NNN_* denotes the trinucleotide normalized frequency. The 12 physicochemical properties for DNA trinucleotide are listed in **Table 11**. The TPCP descriptor has been successfully applied to DNA N4-Methylcytosine site prediction (50).

*Parameter(s)*:

- *Tri-DNA physicochemical indices:* *the names of used physicochemical trinucleotides indices.*

**Table 11.** The names of the 12 physicochemical trinucleotides indices for DNA.

| Dnase I | Bendability (DNAse) | Bendability (consensus) | Trinucleotide GC Content |
| --- | --- | --- | --- |
| Nucleosome positioning | Consensus_roll | Consensus-Rigid | Dnase I-Rigid |
| MW-Daltons | MW-kg | Nucleosome | Nucleosome-Rigid |

**TPCP type2 (Trinucleotide physicochemical properties**)

For TPCP type2 descriptor, it can be encoded into the following matrix:

$$V=\left[ \begin{matrix} {PC}^{1}(N_{1}N_{2}N_{3}) & \ldots& {PC}^{1}(N_{L-2}N_{L-1}N_{L}) \\ \ldots& \ldots& \ldots\\ {PC}^{j}(N_{1}N_{2}N_{3}) & \ldots& {PC}^{j}(N_{L-2}N_{L-1}N_{L}) \end{matrix} \right]$$

Where *PC_j_*(*N_i_*, *N_i+1_,N_i+2_*) is the *j*-th physicochemical value of the trinucleotide *N_i_N_i+1_,N_i+2_*. *L* is the length of sequence. The 12 physicochemical properties for DNA trinucleotide are listed in **Table 11**. The TPCP type2 descriptor has been successfully applied to DNA N4-Methylcytosine site prediction (51).

*Parameter(s)*:

- *Tri-DNA physicochemical indices:* *the names of used physicochemical trinucleotides indices.*

**MMI (Multivariate mutual information)**

In order to use multivariate mutual information on a DNA/RNA sequence, the ‘2-mer’ set T2 = { AA, AC, AG, AT, CC, CG, CT, GG, GT, TT} and ‘3-mer’ set T3 = {AAA, AAC, AAG, AAT, ACC, ACG, ACT, AGG, AGT, ATT, CCC, CCG, CCT, CGG, CGT, CTT, GGG, GGT, GTT and TTT} were defined, and then the mutual information can be calculated as follows:

$$I\left( N_{1}N_{2} \right)=f\left( N_{1}N_{2} \right)ln\frac{f\left( N_{1}N_{2} \right)}{f(N_{1})f(N_{2})}$$

$I\left( N_{1}N_{2}N_{3} \right)=f\left( N_{1}N_{2} \right)ln\frac{f\left( N_{1}N_{2} \right)}{f(N_{1})f(N_{2})}+\frac{f\left( N_{1}N_{3} \right)}{f(N_{3})}ln\frac{f\left( N_{1}N_{3} \right)}{f(N_{3})}$-$\frac{f\left( N_{1}N_{2}N_{3} \right)}{f(N_{2}N_{3})}ln\frac{f\left( N_{1}N_{2}N_{3} \right)}{f(N_{2}N_{3})}$

where *f(N_i_)* is the frequency of *N_i_* in the sequence; *f(N_i_, N_j_)* is the frequency of the T2’s element *N_i_N_j_*; *f(N_i_, N_j_, N_k_)* are the frequency of the T3’s element *N_i_N_j_N_k_*. The MMI descriptor has been successfully applied to DNA N4-Methylcytosine site prediction (51,82).

**Z_curve_9bit (The Z curve parameters for frequencies of phase-specific mononucleotides)**

The frequencies of bases A, C, G and T occurring in an open reading frame or a fragment of DNA sequence with base at position 1, 4, 7, …; 2, 5, 8, …; 3, 6, 9, …, are denoted by *a_1_*, *c_1_*, *g_1_*, *t_1_*; *a_2_,* *c_2_*, *g_2_*, *t_2_*; *a_3_*, *c_3_*, *g_3_*, *t_3_,* respectively. They are in fact the frequencies of bases at the 1st, 2nd and 3rd codon positions. *a_i_*, *c_i_*, *g_i_*, *t_i_* are mapped onto a point *P_i_* in a three-dimensional space *V_i_*, *i*=1, 2, 3. The coordinates of *P_i_*, denoted by *x_i_*, *y_i_*, *z_i_*, are determined by the Z-transform of DNA sequences:

$$\left\{ \begin{matrix} x_{i}=\left( a_{i}+g_{i} \right)-\left( c_{i}+t_{i} \right), \\ y_{i}=\left( a_{i}+c_{i} \right)-\left( g_{i}+t_{i} \right), \\ \begin{matrix} z_{i}=\left( a_{i}+t_{i} \right)-\left( g_{i}+c_{i} \right), \\ x_{i},y_{i}, z_{i},\in\left[ -1, 1 \right], i=1,2,3. \end{matrix} \end{matrix} \right.$$

**Z_curve_12bit (The Z curve parameters for frequencies of phase-independent di-nucleotides)**

The Z_curve_12bit descriptor consider the frequency of dinucleotides, denoted by *p(XY)*, where X, Y = A, C, G and T. This descriptor can be calculated as follows:

$$\left\{ \begin{matrix} x_{X}=\left( p(XA)+p(XG) \right)-\left( p(XC)+p(XT) \right), \\ y_{X}=\left( p(XA)+p(XC) \right)-\left( p(XG)+p(XT) \right), \\ \begin{matrix} z_{X}=\left( p(XA)+p(XT) \right)-\left( p(XG)+p(XC) \right), \\ X=A,C,G,T, \end{matrix} \end{matrix} \right.$$

**Z_curve_36bit (The Z curve parameters for frequencies of phase-specific di-nucleotides)**

Using similar notations, the Z_curve_36bit descriptor can be calculated as follows:

$$\left\{ \begin{matrix} x_{X}^{k}=\left( p^{k}(XA)+p^{k}(XG) \right)-\left( p^{k}(XC)+p^{k}(XT) \right), \\ y_{X}^{k}=\left( p^{k}(XA)+p^{k}(XC) \right)-\left( p^{k}(XG)+p^{k}(XT) \right), \\ \begin{matrix} z_{X}^{k}=\left( p^{k}(XA)+p^{k}(XT) \right)-\left( p^{k}(XG)+p^{k}(XC) \right), \\ X=A,C,G,T;k=1,2,3, \end{matrix} \end{matrix} \right.$$

**Z_curve_48bit (The Z curve parameters for frequencies of phase-independent tri-nucleotides)**

Using similar notations, the Z_curve_48bit descriptor can be calculated as follows:

$$\left\{ \begin{matrix} x_{XY}=\left( p(XYA)+p(XYG) \right)-\left( p(XYC)+p(XYT) \right), \\ y_{XY}=\left( p(XYA)+p(XYC) \right)-\left( p(XYG)+p(XYT) \right), \\ \begin{matrix} z_{XY}=\left( p(XYA)+p(XYT) \right)-\left( p(XYG)+p(XYC) \right), \\ X=A,C,G,T;Y=A, C,G,T \end{matrix} \end{matrix} \right.$$

**Z_curve_144bit (The Z curve parameters for frequencies of phase-specific tri-nucleotides)**

Using similar notations, the Z_curve_144bit descriptor can be calculated as follows:

$$\left\{ \begin{matrix} x_{XY}^{k}=\left( p^{k}(XYA)+p^{k}(XYG) \right)-\left( p^{k}(XYC)+p^{k}(XYT) \right), \\ y_{XY}^{k}=\left( p^{k}(XYA)+p^{k}(XYC) \right)-\left( p^{k}(XYG)+p^{k}(XYT) \right), \\ \begin{matrix} z_{XY}^{k}=\left( p^{k}(XYA)+p^{k}(XYT) \right)-\left( p^{k}(XYG)+p^{k}(XYC) \right), \\ X=A,C,G,T;Y=A,C,G,T;k=1,2,3. \end{matrix} \end{matrix} \right.$$

The Z_curve descriptor has been successfully applied to short coding sequence identification of human genes (44).

**Autocorrelation**

The Autocorrelation encoding (21) can transform the nucleotide/protein sequences of different lengths into fixed-length vectors by measuring the correlation between any two properties. Autocorrelation encoding can generate two kinds of variables (i.e. The autocorrelation (AC) between the same property, and the cross-covariance (CC) between two different properties). There are six types of autocorrelation encodings for nucleotide sequence, including dinucleotide-based auto covariance (DAC), dinucleotide-based cross covariance (DCC), dinucleotide-based auto-cross covariance (DACC), trinucleotide-based auto covariance (TAC), trinucleotide-based cross covariance (TCC), and trinucleotide-based auto-cross covariance (TACC). The used 148 physicochemical properties for DNA dinucleotide and 22 physicochemical properties for RNA dinucleotide are listed in **Tables 9** and **10**, respectively. The used 12 physicochemical properties for DNA trinucleotide are listed in **Table 11**.

**DAC (Dinucleotide-based auto covariance)**

The Dinucleotide-based Auto Covariance (DAC) encoding (21) measures the correlation of the same physicochemical index between two dinucleotide separated by a distance of *lag* along the sequence. The DAC can be calculated as:

$$DAC\left( u, lag \right)=\sum_{i=1}^{L-lag-1} (\left( P_{u}\left( R_{i}R_{i+1} \right)-\bar{P_{u}} \right)(P_{u}\left( R_{i+lag}R_{i+lag+1} \right)-\bar{P_{u}})/(L-lag-1)),$$

where *u* is a physicochemical index, *L* is the length of the nucleotide sequence,$P_{u}\left( R_{i}R_{i+1} \right)$ is the numerical value of the physicochemical index *u* for the dinucleotide *R_i_R_i+1_* at position *i*, $\bar{P_{u}}$ is the average value for physicochemical index *u* along the whole sequence:

$$\bar{P_{u}}=\sum_{j=1}^{L-1} P_{u}\left( R_{j}R_{j+1} \right)/(L-1).$$

The dimension of the DAC feature vector is *N*×LAG, where *N* is the number of physicochemical indices and LAG is the maximum of *lag* (*lag* = 1, 2, …, LAG).

*Parameter(s)*:

- *Di-DNA physicochemical indices:* *the names of used physicochemical dinucleotides indices. (For DNA sequences)*
- *Di-RNA physicochemical indices:* *the names of used physicochemical dinucleotides indices. (For RNA sequences)*
- *Lag value: the number of nucleotides between two di-nucleotides.*

**DCC (Dinucleotide-based Cross Covariance)**

The Dinucleotide-based Cross Covariance (DCC) encoding (21) measures the correlation of two different physicochemical indices between two dinucleotides separated by *lag* nucleic acids along the sequence. The DCC encoding is calculated as:

$$\mathrm{DCC}\left( u_{1},u_{2},lag \right)=\sum_{i=1}^{L-lag-1} \left( P_{u_{1}}\left( R_{i}R_{i+1} \right)-\bar{P}_{u_{1}} \right)(P_{u_{2}}\left( R_{i+lag}R_{i+lag+1} \right)-\bar{P}_{u_{2}})/(L-lag-1),$$

where *u_1_* and *u_2_* are different physicochemical indices, *L* is the length of the nucleotide sequence, $P_{u_{a}}\left( R_{i}R_{i+1} \right)$ is the numerical value of the physicochemical index *u_a_* for the dinucleotide *R_i_R_i+1_* at position *i,* $\bar{P}_{u_{a}}$ is the average value for physicochemical index *u_a_* along the whole sequence:

$$\bar{P}_{u_{a}}=\sum_{j=1}^{L-1} P_{u_{a}}\left( R_{j}R_{j+1} \right)/(L-1).$$

The dimension of the DCC feature vector is *N*×(*N*-1)×LAG, where *N* is the number of physicochemical indices and LAG is the maximum of *lag* (*lag* = 1, 2, …, LAG).

*Parameter(s)*:

- *Di-DNA physicochemical indices:* *the names of used physicochemical dinucleotides indices. (For DNA sequences)*
- *Di-RNA physicochemical indices:* *the names of used physicochemical dinucleotides indices. (For RNA sequences)*
- *Lag value: the number of nucleotides between two di-nucleotides.*

**DACC (Dinucleotide-based Auto-Cross Covariance)**

The Dinucleotide-based Auto-Cross Covariance (DACC) encoding (21) is a combination of DAC and DCC encoding. Thus, the dimension of the DACC encoding is *N*×*N*×LAG, where *N* is the number of physicochemical indices and LAG is the maximum of the *lag* (*lag* = 1, 2, …, LAG).

*Parameter(s)*:

- *Di-DNA physicochemical indices:* *the names of used physicochemical dinucleotides indices. (For DNA sequences)*
- *Di-RNA physicochemical indices:* *the names of used physicochemical dinucleotides indices. (For RNA sequences)*
- *Lag value: the number of nucleotides between two di-nucleotides.*

*.*

**TAC (Trinucleotide-based Auto Covariance)**

The Trinucleotide-based Auto Covariance (TAC) encoding measures the correlation of the same physicochemical index between trinucleotides separated by lag nucleic acids along the sequence, and can be calculated as:

$$TAC\left( lag, u \right)=\sum_{i=1}^{L-lag-2} (P_{u}\left( R_{i}R_{i+1}R_{i+2} \right)-\bar{P}_{u})(P_{u}\left( R_{i+lag}R_{i+lag+1}R_{i+lag+2} \right)-\bar{P}_{u})/(L-lag-2),$$

where *u* is a physicochemical index, *L* is the length of the nucleotide sequence,$P_{u}\left( R_{i}R_{i+1}R_{i+2} \right)$ is the numerical value of the physicochemical index *u* for the trinucleotide *R_i_R_i+1_R_i+2_* at position *i*, $\bar{P_{u}}$ is the average value for physicochemical index *u* along the whole sequence:

$$\bar{P_{u}}=\sum_{j=1}^{L-2} P_{u}\left( R_{i}R_{i+1}R_{i+2} \right)/(L-2).$$

The dimension of the TAC feature vector is *N*×LAG, where *N* is the number of physicochemical indices and LAG is the maximum of *lag* (*lag* = 1, 2, …, LAG).

*Parameter(s)*:

- *Tri-DNA physicochemical indices:* *the names of used physicochemical trinucleotides indices.*
- *Lag value: the number of nucleotides between two tri-nucleotides.*

**TCC (Trinucleotide-based Cross Covariance)**

The Trinucleotide-based Cross Covariance (TCC) encoding measures the correlation of two different physicochemical indices between two trinucleotides separated by *lag* nucleic acids along the sequence. The TCC encoding can be calculated as:

$$\mathrm{DCC}\left( u_{1},u_{2},lag \right)=\sum_{i=1}^{L-lag-2} \left( P_{u_{1}}\left( R_{i}R_{i+1}R_{i+2} \right)-\bar{P}_{u_{1}} \right)(P_{u_{2}}\left( R_{i+lag}R_{i+lag+1}R_{i+lag+2} \right)-\bar{P}_{u_{2}})/(L-lag-2)$$

where *u_1_* and *u_2_* are different physicochemical indices, *L* is the length of the nucleotide sequence, $R_{i}R_{i+1}R_{i+2}$ is the numerical value of the physicochemical index *u_a_* for the dinucleotide *R_i_R_i+1_R_i+2_* at position *i,* $\bar{P}_{u_{a}}$ is the average value for physicochemical index *u_a_* along the whole sequence:

$$\bar{P}_{u_{a}}=\sum_{j=1}^{L-1} P_{u_{a}}\left( R_{j}R_{j+1}R_{j+2} \right)/(L-2)$$

The dimension of the DCC feature vector is *N*×(*N*-1)×LAG, where *N* is the number of physicochemical indices and LAG is the maximum of *lag* (*lag* = 1, 2, …, LAG).

*Parameter(s)*:

- *Tri-DNA physicochemical indices:* *the names of used physicochemical trinucleotides indices.*
- *Lag value: the number of nucleotides between two tri-nucleotides.*

**TACC (Trinucleotide-based Auto-Cross Covariance)**

Like DAC encoding, the Trinucleotide-based Auto-Cross Covariance (TACC) encoding (21) is a combination of TAC and TACC encoding. Thus, the dimension of the TACC encoding is *N*×*N*×LAG, where *N* is the number of physicochemical indices and LAG is the maximum of the *lag* (*lag* = 1, 2, …, LAG). There are three types of autocorrelation encodings for protein sequence, including auto covariance (AC), cross covariance (CC), auto-cross covariance (ACC), The amino acid properties used here are different types of amino acids index, which is retrieved from the AAindex Database (83) available at <http://www.genome.jp/dbget/aaindex.html>. The Autocorrelation descriptor has been successfully applied to protein fold recognition (22) and protein-protein interaction prediction (23).

*Parameter(s)*:

- *Tri-DNA physicochemical indices:* *the names of used physicochemical trinucleotides indices.*
- *Lag value: the number of nucleotides between two tri-nucleotides.*

**PseDNC (Pseudo dinucleotide composition)**

The Pseudo Dinucleotide Composition (PseDNC) encoding (84) incorporate contiguous local sequence-order information and the global sequence-order information into the feature vector of the nucleotide sequence. The PseDNC encoding is defined:

$$D={[d_{1},d_{2},\ldots,d_{16},d_{16+1},\ldots,d_{16+1},\ldots,d_{16+\lambda}]}^{T},$$

where

$$d_{k}=\left\{ \begin{aligned} \frac{f_{k}}{\sum_{i=1}^{16} f_{i}+w\sum_{j=1}^{\lambda} \theta_{j}}, (1\leq k\leq16) \\ \frac{w\theta_{k-16}}{\sum_{i=1}^{16} f_{i}+w\sum_{j=1}^{\lambda} \theta_{j}}, (17\leq k\leq16+\lambda) \end{aligned}, \right.$$

where *f_k_* (k=1, 2, …, 16) is the normalized occurrence frequency of dinucleotide in the nucleotide sequence, λ represent the highest counted rank (or tie) of the correlation along the nucleotide sequence, *w* is the weight factor ranged from 0 to 1, and *θ_j_* (*j* =1,2, …, λ) is the *j*-tier correlation factor and is defined:

$$\left\{ \begin{aligned} {\theta_{1}=\frac{1}{L-2}\sum_{i=1}^{L-2} \Theta\left( R_{i}R_{i+1},R_{i+1}R_{i+2} \right) \atop\theta_{2}=\frac{1}{L-3}\sum_{i=1}^{L-3} \Theta\left( R_{i}R_{i+1},R_{i+2}R_{i+3} \right)} \\ {\theta_{3}=\frac{1}{L-4}\sum_{i=1}^{L-4} \Theta\left( R_{i}R_{i+1},R_{i+3}R_{i+4} \right) \atop\cdots} \\ \theta_{\lambda}=\frac{1}{L-1-\lambda}\sum_{i=1}^{L-1-\lambda} \Theta\left( R_{i}R_{i+1},R_{i+\lambda}R_{i+\lambda+1} \right) \end{aligned}(\lambda<L) \right.$$

where the correlation function is defined:

$$\Theta\left( R_{i}R_{i+1},R_{j+1}R_{j+1} \right)=\frac{1}{\mu}\sum_{u=1}^{\mu} {[P_{u}\left( R_{i}R_{i+1} \right)-P_{u}\left( R_{j}R_{j+1} \right)]}^{2}$$

where *μ* is the number of physicochemical indices. $P_{u}\left( R_{i}R_{i+1} \right)$ is the numerical value of the *u*-th (*u*=1, 2, …, *μ*) physicochemical index of the dinucleotide $R_{i}R_{i+1}$ at position *i* and $P_{u}\left( R_{j}R_{j+1} \right)$ represents the corresponding value of the dinucleotide $R_{j}R_{j+1}$ at position *j*. The PseDNC descriptor has been successfully applied to recombination spot identification (84).

*Parameter(s)*:

- *Lambda value: the highest counted rank of the correlation along the nucleotide sequence, default is 2.*
- *Weight value: weight factor, ranged from 0 to 1, default is 0.1.*

**PCPseTNC (Parallel correlation pseudo trinucleotide composition)**

The Parallel Correlation Pseudo Trinucleotide Composition (PCPseTNC) encoding (85,86) is defined as:

$$D={[d_{1},d_{2},\ldots,d_{64},d_{64+1},\ldots,d_{64+\lambda}]}^{T},$$

where

$$d_{k}=\left\{ \begin{aligned} \frac{f_{k}}{\sum_{i=1}^{64} f_{i}+w\sum_{j=1}^{\lambda} \theta_{j}} , (1\leq k\leq64) \\ \frac{w\theta_{k-64}}{\sum_{i=1}^{64} f_{i}+w\sum_{j=1}^{\lambda} \theta_{j}} , (65\leq k\leq64+\lambda) \end{aligned} \right.,$$

where *f_k_* (k=1, 2, …, 64) is the normalized occurrence frequency of trinucleotide in the DNA sequence, λ represent the highest counted rank (or tie) of the correlation along the DNA sequence, *w* is the weight factor ranged from 0 to 1, and *θ_j_* (*j* =1,2, …, λ) is the *j*-tier correlation factor and is defined:

$$\left\{ \begin{aligned} {\theta_{1}=\frac{1}{L-3}\sum_{i=1}^{L-3} \Theta\left( R_{i}R_{i+1}R_{i+2},R_{i+1}R_{i+2}R_{i+3} \right) \atop\theta_{2}=\frac{1}{L-4}\sum_{i=1}^{L-4} \Theta\left( R_{i}R_{i+1}R_{i+2},R_{i+2}R_{i+3}R_{i+4} \right)} \\ {\theta_{3}=\frac{1}{L-5}\sum_{i=1}^{L-5} \Theta\left( R_{i}R_{i+1}R_{i+2},R_{i+3}R_{i+4}R_{i+5} \right) \atop\cdots} \\ \theta_{\lambda}=\frac{1}{L-2-\lambda}\sum_{i=1}^{L-2-\lambda} \Theta\left( R_{i}R_{i+1}R_{i+2},R_{i+\lambda}R_{i+\lambda+1}R_{i+\lambda+2} \right) \end{aligned}(\lambda<L) \right.,$$

where the correlation function is defined:

$$\Theta\left( R_{i}R_{i+1}R_{i+2},R_{j+1}R_{j+1}R_{j+2} \right)=\frac{1}{\mu}\sum_{u=1}^{\mu} {[P_{u}\left( R_{i}R_{i+1}R_{i+2} \right)-P_{u}\left( R_{j}R_{j+1}R_{j+2} \right)]}^{2},$$

where *μ* is the number of physicochemical indices. $P_{u}\left( R_{i}R_{i+1}R_{i+2} \right)$ is the numerical value of the *u*-th (*u*=1, 2, …, *μ*) physicochemical index of the dinucleotide $R_{i}R_{i+1}R_{i+2}$ at position *i* and $P_{u}\left( R_{j}R_{j+1}R_{j+2} \right)$ represents the corresponding value of the dinucleotide $R_{j}R_{j+1}R_{j+2}$ at position *j*. The PCPseTNC descriptor has been successfully applied to recombination spot identification (86).

*Parameter(s)*:

- *Lambda value: the highest counted rank of the correlation along the nucleotide sequence, default is 2.*
- *Weight value: weight factor, ranged from 0 to 1, default is 0.1.*
- *Tri-DNA physicochemical indices:* *the names of used physicochemical trinucleotides indices.*

**SCPseDNC (Series correlation pseudo dinucleotide composition)**

The Series Correlation Pseudo Dinucleotide Composition (SCPseDNC) encoding (85) is defined as:

$$D={[d_{1},d_{2},\ldots,d_{16},d_{16+1},\ldots,d_{16+\lambda},d_{16+\lambda+1},\ldots,d_{16+\lambda\Lambda}]}^{T},$$

where

$$d_{k}=\left\{ \begin{aligned} \frac{f_{k}}{\sum_{i=1}^{16} f_{i}+w\sum_{j=1}^{\lambda} \theta_{j}}, (1\leq k\leq16) \\ \frac{w\theta_{k-16}}{\sum_{i=1}^{16} f_{i}+w\sum_{j=1}^{\lambda\Lambda} \theta_{j}}, (17\leq k\leq16+\lambda\Lambda) \end{aligned}, \right.$$

where *f_k_* (k=1, 2, …, 16) is the normalized occurrence frequency of dinucleotide in the nucleotide sequence, λ represent the highest counted rank (or tie) of the correlation along the nucleotide sequence, *w* is the weight factor ranged from 0 to 1, Λ is the number of physicochemical indices and *θ_j_* (*j* =1,2, …, λ) is the *j*-tier correlation factor and is defined:

$$\left\{ \begin{aligned} {\theta_{1}=\frac{1}{L-3}\sum_{i=1}^{L-3} J_{i,i+1}^{1} \atop\theta_{2}=\frac{1}{L-3}\sum_{i=1}^{L-3} J_{i,i+1}^{2}} \\ {\ldots\atop\theta_{\Lambda}=\frac{1}{L-3}\sum_{i=1}^{L-3} J_{i,i+1}^{\Lambda}} \\ {\begin{matrix} \ldots\\ \theta_{\lambda\Lambda}=\frac{1}{L-\lambda-2}\sum_{i=1}^{L-\lambda-2} J_{i,i+\lambda}^{\Lambda-1} \end{matrix} \atop\theta_{\lambda\Lambda}=\frac{1}{L-\lambda-2}\sum_{i=1}^{L-\lambda-2} J_{i,i+\lambda}^{\Lambda}} \end{aligned}(\lambda<L-2) \right.,$$

where the correlation function is defined:

$$\left\{ \begin{aligned} J_{i,i+m}^{\zeta}=P_{u}(R_{i}R_{i+1})P_{u}(R_{i+m}R_{i+m+1}) \\ \zeta=1,2,\ldots,\Lambda;m=1,2,\ldots,\lambda;i=1,2,\ldots,L-\lambda-2 \end{aligned} \right.,$$

where *μ* is the number of physicochemical indices. $P_{u}\left( R_{i}R_{i+1} \right)$ is the numerical value of the *u*-th (*u*=1, 2, …, *μ*) physicochemical index of the dinucleotide $R_{i}R_{i+1}$ at position *i* and $P_{u}\left( R_{j}R_{j+1} \right)$ represents the corresponding value of the dinucleotide $R_{j}R_{j+1}$ at position *j*. The SCPseDNC descriptor has been successfully applied to recombination spot identification (86).

*Parameter(s)*:

- *Lambda value: the highest counted rank of the correlation along the nucleotide sequence, default is 2.*
- *Weight value: weight factor, ranged from 0 to 1, default is 0.1.*
- *Di-DNA physicochemical indices:* *the names of used physicochemical dinucleotides indices. (For DNA sequences)*
- *Di-RNA physicochemical indices:* *the names of used physicochemical dinucleotides indices. (For RNA sequences)*

**SCPseTNC (Series correlation pseudo trinucleotide composition)**

The Series Correlation Pseudo Trinucleotide Composition (SCPseTNC) encoding (85) is defined as:

$$D={[d_{1},d_{2},\ldots,d_{64},d_{64+1},\ldots,d_{64+\lambda},d_{64+\lambda+1},d_{64+\lambda+1},\ldots,d_{64+\lambda\Lambda}]}^{T},$$

where

$$d_{k}=\left\{ \begin{aligned} \frac{f_{k}}{\sum_{i=1}^{64} f_{i}+w\sum_{j=1}^{\lambda} \theta_{j}} , (1\leq k\leq64) \\ \frac{w\theta_{k-64}}{\sum_{i=1}^{64} f_{i}+w\sum_{j=1}^{\lambda\Lambda} \theta_{j}} , (65\leq k\leq64+\lambda\Lambda) \end{aligned}, \right.$$

where *f_k_* (*k*=1, 2, …, 64) is the normalized occurrence frequency of trinucleotide in the DNA sequence, λ represent the highest counted rank (or tie) of the correlation along the DNA sequence, *w* is the weight factor ranged from 0 to 1, Λ is the number of physicochemical indices and *θ_j_* (*j* =1,2, …, λ) is the *j*-tier correlation factor and is defined:

$$\left\{ \begin{aligned} {\theta_{1}=\frac{1}{L-4}\sum_{i=1}^{L-4} J_{i,i+1}^{1} \atop\theta_{2}=\frac{1}{L-4}\sum_{i=1}^{L-4} J_{i,i+1}^{2}} \\ {\ldots\atop\theta_{\Lambda}=\frac{1}{L-4}\sum_{i=1}^{L-4} J_{i,i+1}^{\Lambda}} \\ {\begin{matrix} \ldots\\ \theta_{\lambda\Lambda}=\frac{1}{L-\lambda-3}\sum_{i=1}^{L-\lambda-3} J_{i,i+\lambda}^{\Lambda-1} \end{matrix} \atop\theta_{\lambda\Lambda}=\frac{1}{L-\lambda-3}\sum_{i=1}^{L-\lambda-3} J_{i,i+\lambda}^{\Lambda}} \end{aligned}(\lambda<L-3) \right.,$$

where the correlation function is defined:

$$\left\{ \begin{aligned} J_{i,i+m}^{\zeta}=P_{u}(R_{i}R_{i+1})P_{u}(R_{i+m}R_{i+m+1}R_{i+m+2}) \\ \zeta=1,2,\ldots,\Lambda;m=1,2,\ldots,\lambda;i=1,2,\ldots,L-\lambda-3 \end{aligned} \right.,$$

where *μ* is the number of physicochemical indices. $P_{u}\left( R_{i}R_{i+1}R_{i+2} \right)$ is the numerical value of the *u*-th (*u*=1, 2, …, *μ*) physicochemical index of the dinucleotide $R_{i}R_{i+1}R_{i+2}$ at position *i* and $P_{u}\left( R_{j}R_{j+1}R_{j+2} \right)$ represents the corresponding value of the dinucleotide $R_{j}R_{j+1}R_{j+2}$ at position *j*.

*Parameter(s)*:

- *Lambda value: the highest counted rank of the correlation along the nucleotide sequence, default is 2.*
- *Weight value: weight factor, ranged from 0 to 1, default is 0.1.*
- *Tri-DNA physicochemical indices:* *the names of used physicochemical trinucleotides indices.*

**KNN (K-Nearest Neighbors)**

The *K*-Nearest Neighbor for peptides (KNN) descriptor (38) requires an extra training file and a label file. The training file is used to calculate the top *K*-Nearest Neighbor peptides by calculating the similarity score of two peptide sequences.

**PSTNPss (Position-specific trinucleotide propensity based on single-strand)**

The Position-specific trinucleotide propensity based on single-strand (PSTNPss) (87,88) uses a statistical strategy based on single-stranded characteristics of DNA or RNA. There are 4^3^ = 64 trinucleotides: AAA, AAC, AAG, ..., TTT(UUU). Thus, for an *L* bp sample, its details of the trinucleotides position specificity can be expressed by the following 64 × (*L*-2) matrix:

$$Z=\left[ \begin{matrix} z_{1,1} & z_{1,2} & \begin{matrix} \cdots& z_{1,L-2} \end{matrix} \\ z_{2,1} & z_{2,2} & \begin{matrix} \cdots& z_{2,L-2} \end{matrix} \\ \begin{matrix} \vdots\\ z_{64,1} \end{matrix} & \begin{matrix} \vdots\\ z_{64,2} \end{matrix} & \begin{matrix} \begin{matrix} \cdots\\ \cdots\end{matrix} & \begin{matrix} \vdots\\ z_{64,L-2} \end{matrix} \end{matrix} \end{matrix} \right]$$

where

$$z_{i,j}=F^{+}\left( {3mer}_{i} | j \right)-F^{-}\left( {3mer}_{i} | j \right), i=1,2,\ldots,64;j=1,2,\ldots L-2$$

$F^{+}\left( {3mer}_{i} | j \right)$ and $F^{-}\left( {3mer}_{i} | j \right)$ denote the frequency of the *i*-th trinucleotide (3mer_i_) at the *j*-th position appear in the positive (*S^+^*) and negative (*S^−^*) data sets, respectively. In the formula, 3mer_1_ equals AAA,3mer_2_ equals AAC, …, 3mer_64_ equals TTT.

Therefore, the sample can be expressed as:

$$S=\left[ \emptyset_{1,}\emptyset_{2},{\ldots,\emptyset}_{L-2} \right]^{T}$$

where T is the operator of transpose and *ϕ_u_* was defined as follows:

$$\emptyset_{u}=\left\{ \begin{matrix} \begin{matrix} z_{1,u}, when N_{u}N_{u+1}N_{u+2}=AAA \\ z_{2,u}, when N_{u}N_{u+1}N_{u+2}=AAG \end{matrix} \\ \begin{matrix} \vdots\\ z_{64,u}, when N_{u}N_{u+1}N_{u+2}=TTT \end{matrix} \end{matrix} \right.$$

**PSTNPds (Position-specific trinucleotide propensity based on double-strand)**

The position-specific trinucleotide propensity based on double-strand (PSTNPss) (87,88) using a statistical strategy based on double-stranded characteristics of DNA according to complementary base pairing, so they have more evident statistical features. At this point, we deem A and T as identical, the same to C and G. Thus, for each sample, it can be converted into a sequence contained A and T only.

# 11. Commonly Used Feature Descriptors and Parameters for Protein or Peptide Sequences

**AAC (Amino Acid Composition)**

The Amino Acid Composition (AAC) encoding (2) calculates the frequency of each amino acid type in a protein or peptide sequence.

**EAAC (Enhanced Amino Acid Composition)**

The Enhanced Amino Acid Composition (EAAC) feature calculates the AAC based on the sequence window of fixed length that continuously slides from the N- to C-terminus of each peptide and can be usually applied to encode the peptides with an equal length.

*Parameters:*

- *Sliding window size:* the length of the sliding window, default is 5.

**EGAAC (Enhanced GAAC)**

The Enhanced GAAC (EGAAC) is also for the first time proposed in this work. It calculates GAAC in windows of fixed length continuously sliding from the N- to C-terminal of each peptide and is usually applied to peptides with an equal length.

*Parameters:*

- *Sliding window size:* the length of the sliding window, default is 5.

**CKSAAP type 1 (Composition of *k*-spaced Amino Acid Pairs type 1)**

The CKSAAP type 1 feature encoding calculates the frequency of amino acid pairs separated by any *k* residues (*k* = 0, 1, 2, … , 5. The default maximum value of *k* is 5) (5,6,89,90).

*Parameters:*

- *Gap value:* *the length of k-spaced amino acids, default is 3.*

**CKSAAP type 2 (Composition of *k*-spaced Amino Acid Pairs type 2)**

The CKSAAP type 2 feature encoding calculates the raw count of amino acid pairs separated by any *k* residues.

*Parameters:*

- *Gap value:* *the length of k-spaced amino acids, default is 3.*

**CKSAAGP type 1 (Composition of k-Spaced Amino Acid Group Pairs type 1)**

CKSAAGP type 1 is a variation of the CKSAAP descriptor, which is our own proposal. It calculates the frequency of amino acid group pairs separated by any *k* residues.

*Parameters:*

- *Gap value:* *the length of k-spaced amino acids, default is 3.*

**CKSAAGP type 2 (Composition of *k*-Spaced Amino Acid Group Pairs type 2)**

It calculates the raw count of amino acid group pairs separated by any *k* residues.

*Parameters:*

- *Gap value:* *the length of k-spaced amino acids, default is 3.*

**DPC type 1 (Di-Peptide Composition type 1)**

The Dipeptide Composition (7) gives 400 descriptors. It is defined as:

$$D(r,s)=\frac{N_{rs}}{N-1}, r,s\in\{A,C,D,\ldots Y\}$$

where *N_rs_* is the number of dipeptides represented by amino acid types r and s.

**DPC type 2 (Di-Peptide Composition type 2)**

DPC type 2 calculate the raw count of the 400 di-peptides in a sequence.

**GDPC type 1 (Grouped Di-Peptide Composition type 1)**

The Grouped Di-Peptide Composition encoding is another variation of the DPC descriptor. It is composed of a total of 25 descriptors that are defined as:

$$f(r,s) = \frac{N_{rs}}{N-1}, r, s \in\{g1, g2, g3,g4,g5\}$$

where *N_rs_* is the number of tripeptides represented by amino acid type groups *r* and *s*, *N* is the length of a protein or peptide sequence.

**GDPC type 2 (Grouped Di-Peptide Composition type 2)**

GDPC type 2 calculates the raw count of the 25 grouped amino acid pairs.

**TPC type 1 (Tri-Peptide Composition type 1)**

The Tripeptide Composition (TPC) (2) gives 8000 descriptors, defined as:

$$f(r,s,t) = \frac{N_{rst}}{N-2}, r, s, t \in\{A, C, D,..., Y\}$$

where *N_rst_* is the number of tripeptides represented by amino acid types *r*, *s* and *t.*

**TPC type 2 (Tri-Peptide Composition type 2)**

TPCP type 2 calculates the raw count of the 8000 tripeptides.

**GTPC type 1 (Grouped Tri-Peptide Composition type 1)**

The Grouped Tri-Peptide Composition encoding is also a variation of TPC descriptor, which generates 125 descriptors, defined as:

$$f(r,s,t) = \frac{N_{rst}}{N-2}, r, s, t \in\{g1, g2, g3,g4,g5\}$$

where *N_rst_* is the number of tripeptides represented by amino acid type groups *r*, *s* and *t*. *N* is the length of a protein or peptide sequence.

**GTPC type 2 (Grouped Tri-Peptide Composition type 2)**

GTPC type 2 calculates the raw count of the 125 grouped amino acid tripeptides.

**DDE (Dipeptide Deviation from Expected Mean)**

The Dipeptide Deviation from Expected Mean feature vector (7) is constructed by computing three parameters, i.e. dipeptide composition (*D_c_*), theoretical mean (*T_m_*), and theoretical variance (*T_v_*). The above three parameters and the DDE are computed as follows. *D_c_(r,s)*, the dipeptide composition measure for the dipeptide ‘*rs’*, is given as

$$D_{c}(r,s)=\frac{N_{rs}}{N-1}, r,s\in\{A,C,D,\ldots Y\}$$

where *N_rs_* is the number of dipeptides represented by amino acid types *r* and *s* and *N* is the length of the protein or peptide. *T_m_(r,s)*, the theoretical mean, is given by:

$$T_{m}(r,s)=\frac{C_{r}}{C_{N}}\times\frac{C_{s}}{C_{N}}$$

where *C_r_* is the number of codons that code for the first amino acid and *C_s_* is the number of codons that code for the second amino acid in the given dipeptide ‘*rs*’. *C_N_* is the total number of possible codons, excluding the three stop codons (i.e., 61). *T_v_* (*r,s*), the theoretical variance of the dipeptide ‘*rs*’, is given by:

$$T_{v}(r,s)=\frac{T_{m}(r,s)(1-T_{m}(r,s))}{N-1}$$

Finally, *DDE(r,s)* is calculated as:

$$DDE(r,s)=\frac{D_{c}(r,s)-T_{m}(r,s)}{\sqrt{T_{v}(r,s)}}$$

**Binary**

In the Binary encoding (31,91), each amino acid is represented by a 20-dimensional binary vector, e.g.

A is encoded by (10000000000000000000), C is encoded by (01000000000000000000), …, Y is encoded by (00000000000000000001), respectively. This encoding scheme is often used to encode peptides with an equal length.

**Binary_6bit**

In this descriptor, the 6-letter exchange group {*e_1_*, *e_2_*, *e_3_*, *e_4_*, *e_5_*, *e_6_*} is adopted to represent a protein sequence (16,32), where *e_1_*∈{H, R, K}, *e_2_*∈{D, E, N, D}, *e_3_*∈{C}, *e_4_*∈{S, T, P, A, G}, *e_5_*∈{M, I, L, V}, *e_6_*∈{F, Y, W}. Exchange groups represent conservative replacements through evolution. These exchange groups are effectively equivalence classes of amino acids and are derived from PAM. For example, the protein sequence PVKTNVK can be represented as *e_4_e_5_e_1_e_4_e_2_e_5_e_1_*. Then, each group is represented by a 6-dimensional binary vector, e.g. *e_1_* is encoded by (100000), *e_2_* is encoded by (010000), …, *e_6_* is encoded by (000001).

**Binary_5bit_type 1**

Like binary_6bit descriptor, the 5-letter amino acid group {*e_1_*, *e_2_*, *e_3_*, *e_4_*, *e_5_*} is adopted to represent a protein sequence, and each group is represented by a 5-dimensional binary vector (16,33). *e_1_*∈{G, A, V, L, M, I}, *e_2_*∈{F, Y, W}, *e_3_*∈{K, R, H}, *e_4_*∈{D, E}, *e_5_*∈{S, T, C, P, N, Q}. Then, each group is represented by a 5-dimensional binary vector, e.g. *e_1_* is encoded by (10000), *e_2_* is encoded by (01000), …, *e_5_* is encoded by (00001).

**Binary_5bit_type 2**

For this descriptor, it is based on all the possible ways that ones and zeros can be combined in a five-bit unit. There are 32 possible ways to represent 20 amino acids. When the representations with no or all ones and those with 1 or 4 ones are removed there are exactly twenty representations. And A is encoded by (00011), C (00101), D (00110), E (00111), F (01001), G (01010), H (01011), I (01100), K (01101), L (01110), M (10001), N (10010), P (10011), Q (10100), R (10101), S (10110), T (11000), V (11001), W (11010), Y (11100).

**Binary_3bit_type 1-7**

For this descriptor, the 3-letter amino acid group {*e_1_*, *e_2_*, *e_3_*} is adopted to represent a protein sequence, and each group is represented by a 3-dimensional binary vector (80). Then, each group is represented by a 5-dimensional binary vector, e.g. *e_1_* is encoded by (100), *e_2_* is encoded by (010), *e_3_* is encoded by (001). The division of the amino acids based on physicochemical properties (PRAM900101) in **Table 12**. The difference of the type 1 to type 7 subtypes is the different division of amino acids. The division of amino acids for type 2 to 7 is based on “Normalized van der Waals volume”, “Polarity”, “Polarizability”, “Charge”, “Secondary structure” and “Solvent accessibility” in **Table 12**.

**Table 12.** Amino acid physicochemical attributes and the division of the amino acids into three groups according to each attribute.

| **Attribute** | **Division** | | |
| --- | --- | --- | --- |
| Hydrophobicity_PRAM900101 | Polar: RKEDQN | Neutral: GASTPHY | Hydrophobicity: CLVIMFW |
| Hydrophobicity_ARGP820101 | Polar: QSTNGDE | Neutral: RAHCKMV | Hydrophobicity: LYPFIW |
| Hydrophobicity_ZIMJ680101 | Polar: QNGSWTDERA | Neutral: HMCKV | Hydrophobicity: LPFYI |
| Hydrophobicity_PONP930101 | Polar: KPDESNQT | Neutral: GRHA | Hydrophobicity: YMFWLCVI |
| Hydrophobicity_CASG920101 | Polar: KDEQPSRNTG | Neutral: AHYMLV | Hydrophobicity: FIWC |
| Hydrophobicity_ENGD860101 | Polar: RDKENQHYP | Neutral :SGTAW | Hydrophobicity: CVLIMF |
| Hydrophobicity_FASG890101 | Polar: KERSQD | Neutral: NTPG | Hydrophobicity: AYHWVMFLIC |
| Normalized van der Waals volume | Volume range: 0-2.78  GASTPD | Volume range: 2.95-94.0  NVEQIL | Volume range: 4.03-8.08  MHKFRYW |
| Polarity | Polarity value: 4.9-6.2  LIFWCMVY | Polarity value: 8.0-9.2  PATGS | Polarity value: 10.4-13.0  HQRKNED |
| Polarizability | Polarizability value: 0-1.08  GASDT | Polarizability value: 0.128-120.186  GPNVEQIL | Polarizability value: 0.219-0.409  KMHFRYW |
| Charge | Positive: KR | Neutral: ANCQGHILMFPSTWYV | Negative: DE |
| Secondary structure | Helix: EALMQKRH | Strand: VIYCWFT | Coil: GNPSD |
| Solvent accessibility | Buried: ALFCGIVW | Exposed: PKQEND | Intermediate: MPSTHY |

**AESNN3 (Learn from alignments)**

For this descriptor, each amino acid type is described using a three-dimensional vector. Values are taken from the three hidden units from the neural network trained on structure alignments (16,34). The values are listed in **Table 13**.

**Table 13.** AESNN3 values learning from alignments.

| **Amino acids** | **AESNN3 values** | | |
| --- | --- | --- | --- |
| A | -0.99 | -0.61 | 0.00 |
| R | 0.28 | -0.99 | -0.22 |
| N | 0.77 | -0.24 | 0.59 |
| D | 0.74 | -0.72 | -0.35 |
| C | 0.34 | 0.88 | 0.35 |
| Q | 0.12 | -0.99 | -0.99 |
| E | 0.59 | -0.55 | -0.99 |
| G | -0.79 | -0.99 | 0.10 |
| H | 0.08 | -0.71 | 0.68 |
| I | -0.77 | 0.67 | -0.37 |
| L | -0.92 | 0.31 | -0.99 |
| K | -0.63 | 0.25 | 0.50 |
| M | -0.80 | 0.44 | -0.71 |
| F | 0.87 | 0.65 | -0.53 |
| P | -0.99 | -0.99 | -0.99 |
| S | 0.99 | 0.40 | 0.37 |
| T | 0.42 | 0.21 | 0.97 |
| W | -0.13 | 0.77 | -0.90 |
| Y | 0.59 | 0.33 | -0.99 |
| V | -0.99 | 0.27 | -0.52 |

**Moran**

The autocorrelation descriptors are defined based on the distribution of amino acid properties along the sequence (17,19,20). The amino acid properties used here are different types of amino acids index, which is retrieved from the AAindex Database (83) available at <http://www.genome.jp/dbget/aaindex.html/>. All the amino acid indices are centralized and standardized prior to the calculation:

$P_{r} = \frac{P_{r} - \overline{P}}{\sigma}$,

where is the average of the properties of the 20 amino acids and σ is the standard deviation of the properties of the 20 amino acids. and σ can be calculated as follows:

$\bar{P} = \frac{\sum_{r=1}^{20} P_{r}}{20} , \sigma=\sqrt{\frac{1}{20}\sum_{r=1}^{20} (P_{r}-\bar{P})^{2}}$.

The Moran autocorrelation descriptors (17,18) can thus be defined as:

$I(d) = \frac{\frac{1}{N-d}\sum_{i=1}^{N-d} (P_{i}-\bar{P}^{'})(P_{i+d}-\bar{P}^{'})}{\frac{1}{N}\sum_{i=1}^{N} (P_{i}-\bar{P}^{'})^{2}}, d = 1,2,3...,nlag$,

where *d* is the lag of the autocorrelation, *nlag* is the maximum value of the lag, *P_i_* and *P_i+d_* are the properties of the amino acids at positions *i* and *i + d*, respectively. $\overline{P}'$ is the average of the considered property *P* over the entire sequence of length *N* and is calculated as:

$\bar{P}' = \frac{\sum_{i=1}^{N} P_{i}}{N}$.

The Moran descriptor has been successfully applied to membrane protein type prediction (17) and protein secondary structural content prediction (18).

*Parameters:*

- *Physicochemical properties for proteins: the names of used physicochemical amino acids indices.*

**Geary**

The Geary autocorrelation descriptors for a protein or peptide sequence are defined as:

$C(d) = \frac{\frac{1}{2(N-d)}\sum_{i=1}^{N-d} (P_{i}-P_{i+d})^{2}}{\frac{1}{N-1}\sum_{i=1}^{N} (P_{i}-\bar{P}')^{2}}, d=1,2,...,nlag$,

where *d*, *P*, *P_i_* and *P_i+d_*, *nlag* have the same definitions as described above. The Geary descriptor has been successfully applied to population structure inferring (19).

*Parameters:*

- *Physicochemical properties for proteins: the names of used physicochemical amino acids indices.*

**NMBroto (Normalized moreau-broto autocorrelation)**

The Moreau-Broto autocorrelation descriptors are defined as follows:

$AC(d)=\sum_{i=1}^{N-d} P_{i}\times P_{i+d} , d=1,2,...,nlag$.

The normalized Moreau-Broto autocorrelation descriptors are thus defined as:

$ATS(d)=\frac{AC(d)}{N-d} , d=1,2,...,nlag$.

The NMBroto descriptor has been successfully applied to protein helix content prediction (20).

*Parameters:*

- *Physicochemical properties for proteins: the names of used physicochemical amino acids indices.*

**CTD (Composition/Transition/Distribution)**

The Composition, Transition and Distribution (CTD) features represent the amino acid distribution patterns of a specific structural or physicochemical property in a protein or peptide sequence (8-12). 13 types of physicochemical properties have been previously used for computing these features (**Table 12**). These include hydrophobicity, normalized Van der Waals Volume, polarity, polarizability, charge, secondary structures and solvent accessibility. These descriptors are calculated according to the following procedures: (i) The sequence of amino acids is transformed into a sequence of certain structural or physicochemical properties of residues; (ii) Twenty amino acids are divided into three groups for each of the seven different physicochemical attributes based on the main clusters of the amino acid indices of Tomii and Kanehisa (92). The groups of amino acids are listed in **Table 12**. The Composition/Transition/Distribution descriptors has been successfully applied to protein folding class prediction (10,11), enzyme family classification (9), RNA-binding protein prediction (12), protein structural prediction (92) and anti-cancer peptide prediction (80).

**CTDC**

Taking the hydrophobicity attribute as an example, all amino acids are divided into three groups: polar, neutral and hydrophobic (**Table 12**). The Composition descriptor consists of three values: the global compositions (percentage) of polar, neutral and hydrophobic residues of the protein. An illustrated example of this encoding scheme is provided in the following **Figure 15**. The Composition descriptor can be calculated as follows:

$C(r)=\frac{N(r)}{N} , r\in\{polar,neutral,hydrophobic\}$,

where *N(r)* is the number of amino acid type *r* in the encoded sequence and *N* is the length of the sequence.

**CTDT**

The Transition descriptor T also consists of three values (10,11): A transition from the polar group to the neutral group is the percentage frequency with which a polar residue is followed by a neutral residue or a neutral residue by a polar residue. Transitions between the neutral group and the hydrophobic group and those between the hydrophobic group and the polar group are defined in a similar way. The transition descriptor can then be calculated as:

$T(r,s)=\frac{N(r,s)+N(s,r)}{N-1}, r,s\in\{(polar,neutral),(neutral,hydrophobic),(hydrophobic,polar)\}$,

where *N(r,s)* and *N(s,r)* are the numbers of dipeptides encoded as “*rs*” and “*sr*” respectively in the sequence, while *N* is the length of the sequence. An illustrated example of this encoding scheme is provided in the following **Figure** **15.**


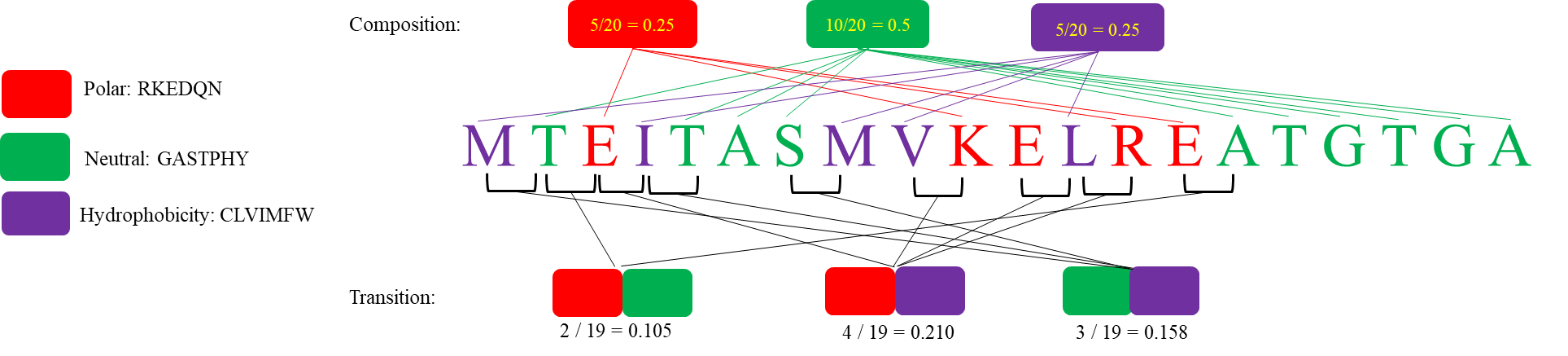


**Figure 15**. An example of the calculation of composition and transition descriptors. This example uses the hydrophobicity attribute.

**CTDD**

The Distribution descriptor consists of five values for each of the three groups (polar, neutral and hydrophobic) (10,11), namely the corresponding fraction of the entire sequence, where the first residue of a given group is located, and where 25, 50, 75 and 100% of occurrences are contained.

For example, we start with the first residue up to and including the residue that marks 25/50/75/100% of occurrences for residues of any given group and then we simply divide the position of this residue by the length of the entire sequence.

**CTriad (Conjoint triad)**

The Conjoint Triad descriptor (CTriad) considers the properties of one amino acid and its vicinal amino acids by regarding any three continuous amino acids as a single unit (13). First, the protein sequence is represented by a binary space (*V, F*), where *V* denotes the vector space of the sequence features, and each feature (*V_i_*) represents a sort of triad type; *F* is the number vector corresponding to *V*, where *f_i_*, the value of the *i-*th dimension of *F*, is the number of type *V_i_* appearing in the protein sequence. For the amino acids that have been catalogued into seven classes, the size of *V* should be equal to 7ⅹ7ⅹ7=343. Accordingly, *i* = 1, 2, 3, …, 343. An illustrated example of this encoding scheme is provided in the following **Figure 16**. In principle, the longer a protein sequence, the higher the probability to have larger values of *f_i_*, confounding the comparison of proteins with different lengths. Thus, we define a new parameter, *d_i_*, by normalizing *f_i_* with the following equation:

$d_{i}=\frac{f_{i}-\min\{f_{1},f_{2},...,f_{343}\}}{\max\{f_{1},f_{2},...,f_{343}\}}$.

The CTriad descriptors has been successfully applied to protein-protein interaction prediction (13).


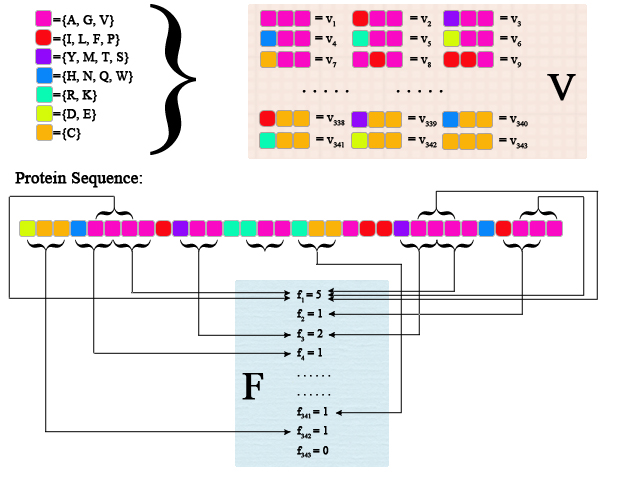


**Figure 16**. Schematic diagram for constructing the vector space (*V, F*) of a given protein sequence. *V* is the vector space of the sequence features; each feature (*V_i_*) represents a triad composed of three consecutive amino acids; *F* is the number vector corresponding to *V*, and the value of the *i-*th entry of *F*, denoted *f_i_*, is the number of occurrences that the triad associated with *V_i_* appearing in the protein sequence. The figure was adapted from the Supplementary Figure in (13).

**KSCTriad (Conjoint k-spaced Triad)**

The *k*-Spaced Conjoint Triad (KSCTriad) descriptor is based on the Conjoint CTriad descriptor, which not only calculates the numbers of three continuous amino acid units, but also considers the continuous amino acid units that are separated by any *k* residues (The default maximum value of *k* is set to 5). For example, AxRxT is a 1-spaced triad. Thus, the dimensionality of the KSCTriad encoded feature vector is 343×(*k*+1). An illustrated example of this encoding scheme is provided in **Figure 17**.


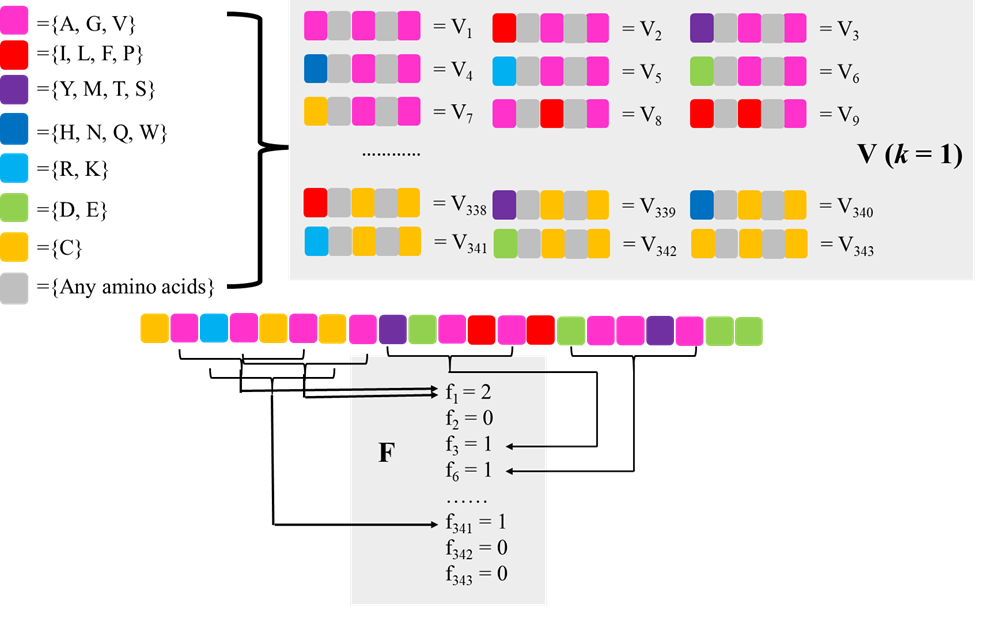


**Figure 17**. A schematic diagram for constructing the vector space (V, F) of protein sequence (*k*=1).

*Parameters:*

- *Gap value:* *the length of k-spaced amino acids, default is 3.*

**SOCNumber (Sequence-Order-Coupling Number)**

The *d*-th rank sequence-order-coupling number is defined as:

$\tau_{d}=\sum_{i=1}^{N-d} (d_{i,i+d})^{2}, d =1,2,3,...,nlag$,

where *d_i,i+d_* is the entry in a given distance matrix describing a distance between the two amino acids at position *i* and *i + d, nlag* denotes the maximum value of the lag (default value: 30) and *N* is the length of a protein or peptide sequence. As distance matrix both the Schneider-Wrede physicochemical distance matrix (26) used by Kuo-Chen Chou, and the chemical distance matrix by Grantham (93) are used. Accordingly, the descriptor dimension will be *nlag*ⅹ2. The quasi-sequence-order descriptors described next also utilizes the two matrices. An illustrated example of this encoding scheme is provided in the following **Figure 18**.

Note: the length of the protein must be not less than the maximum value of *nlag*.

*Parameter(s)*:

- *Lag value: the maximum value of the lag, default value is 3.*


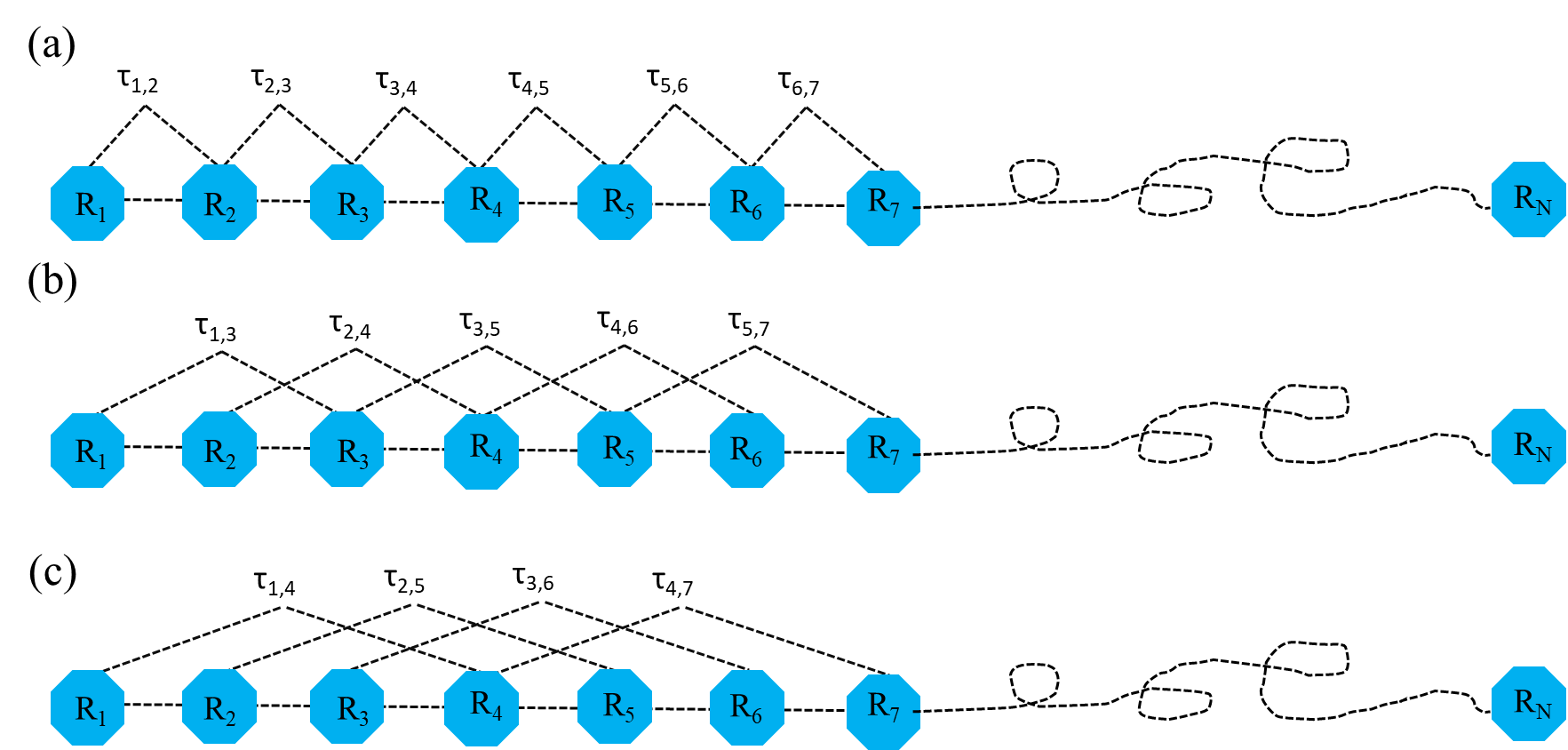


**Figure 18**. A schematic drawing to show (a) the 1st-rank, (b) the 2nd-rank, and (3) the 3rd-rank sequence-order-coupling mode along a protein sequence. (a) reflects the coupling mode between all the most adjacent residues, (b) shows the coupling between the adjacent plus one residue, and (c) shows the coupling between the adjacent plus two residues. This figure is adapted from (24).

**QSOrder (Quasi-sequence-order)**

For each amino acid type, a quasi-sequence-order descriptor can be defined as:

$$X_{r}=\frac{f_{r}}{\sum_{r=1}^{20} f_{r}+w\sum_{d=1}^{nlag} \tau_{d}} , r= 1,2,...,20$$

where *f_r_* is the normalized occurrence of amino acid type *r* and *w* is a weighting factor (*w* = 0.1), *nlag* and $\tau_{d}$have the same definitions as described above. These are the first 20 quasi-sequence-order descriptors. The other 30 quasi-sequence-order descriptors are defined as:

$X_{d}=\frac{w\tau_{d}-20}{\sum_{r=1}^{20} f_{r}+w\sum_{d=1}^{nlag} \tau_{d}} , d=21,22,...,20+nlag$.

The SOCNumber and QSOrder descriptors have been successfully applied to protein subcellular location prediction (25,26).

*Parameter(s)*:

- *Lag value: the maximum value of the lag, default value is 3.*
- *Weight factor: the weight factor value, range from 0 to 1, and default is 0.05.*

**PAAC (Pseudo-Amino Acid Composition)**

This group of descriptors has been proposed in (27,28). Let $H_{1}^{o}(i)$, $H_{2}^{o}(i)$,$M^{o}\left( i \right)$ for *i* = 1, 2, 3, … 20 be the original hydrophobicity values, the original hydrophilicity values and the original side chain masses of the 20 natural amino acids, respectively. They are converted to the following quantities by a standard conversion:

$H_{1}(i)=\frac{H_{1}^{o}(i)-\frac{1}{20}\sum_{i=1}^{20} H_{1}^{o}(i)}{\sqrt{\frac{\sum_{i=1}^{20} [H_{1}^{o}(i)-\frac{1}{20}\sum_{i=1}^{20} H_{1}^{o}(i)]^{2}}{20}}}$,

where $H_{2}^{o}(i)$ and $M^{o}(i)$ are normalized as $H_{2}(i)$ and $M(i)$ in the same manner. An example of the correlation function is provided in the following **Figure 19**.


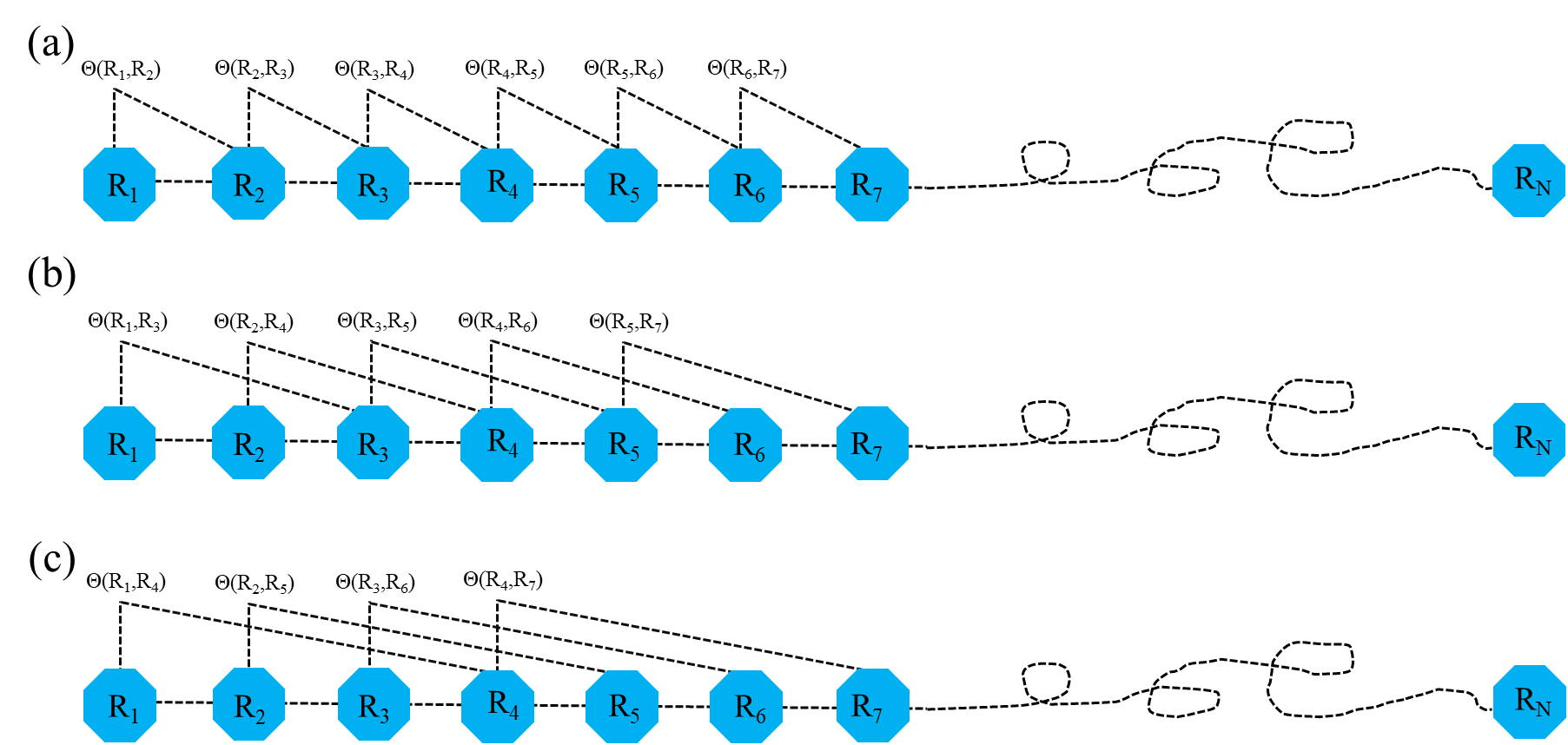


**Figure 19**. A schematic illustration showing (a) the first-tier, (b) the second-tier, and (3) the third-tier sequence order correlation mode along a protein sequence. (a) reflects the coupling mode between all the most adjacent residues, (b) shows the coupling between the adjacent plus one residue, and (c) shows the coupling between the adjacent plus two residues. This figure is adapted from (27) for illustration purposes.

Next, a correlation function can be defined as:

$\Theta(R_{i},R_{j})=\frac{1}{3}\{[H_{1}(R_{i})-H_{1}(R_{j})]^{2}+[H_{2}(R_{i})-H_{2}(R_{j})]^{2}+[M(R_{i})-M(R_{j})]^{2}\}$.

This correlation function is actually an averaged value for the three amino acid properties: hydrophobicity value, hydrophilicity value and side chain mass. Therefore, we can extend this definition of correlation function for one amino acid property or for a set of *n* amino acid properties.

For one amino acid property, the correlation can be defined as:

$\Theta(R_{i},R_{j})=[H_{1}(R_{i})-H_{1}(R_{j})]^{2}$,

where *H(R_i_)* is the amino acid property of amino acid *R_i_* after standardization.

For a set of *n* amino acid properties, it can be defined as:

$\Theta(R_{i},R_{j})=\frac{1}{n}\sum_{n=1}^{n} [H_{k}(R_{i})-H_{k}(R_{j})]^{2}$,

where *H_k_(R_i_)* is the *k*-th property in the amino acid property set for amino acid *R_i_*.

A set of descriptors called sequence order-correlated factors are defined as:

$\theta_{1}=\frac{1}{N-1}\sum_{i=1}^{N-1} \Theta(R_{i},R_{i+1})$,

$\theta_{2}=\frac{1}{N-2}\sum_{i=1}^{N-2} \Theta(R_{i},R_{i+2})$,

$\theta_{3}=\frac{1}{N-3}\sum_{i=1}^{N-3} \Theta(R_{i},R_{i+3})$,

…,

$\theta_{\lambda}=\frac{1}{N-\lambda}\sum_{i=1}^{N-\lambda} \Theta(R_{i},R_{i+\lambda})$,

where λ (λ < *N*) is an integer parameter to be chosen. Let *f_i_* be the normalized occurrence frequency of amino acid *i* in the protein sequence. Then, a set of 20 + λ descriptors called the pseudo-amino acid composition for a protein sequence can be defines as:

$X_{c}=\frac{f_{c}}{\sum_{r=1}^{20} f_{r}+w\sum_{j=1}^{\lambda} \theta_{j}} , (1<c<20)$,

$X_{c}=\frac{w\theta_{c-20}}{\sum_{r=1}^{20} f_{r}+w\sum_{j=1}^{\lambda} \theta_{j}} , (21<c<20+\lambda)$,

where *w* is the weighting factor for the sequence-order effect and is set to *w* = 0.05 as suggested by Chou *et al*. (27).

*Parameter(s)*:

- *Lambda value: integer, should be small than sequence length, default is 2.*
- *Weight value: weight factor, ranged from 0 to 1, default is 0.05.*

**APAAC (Amphiphilic Pseudo-Amino Acid Composition)**

Amphiphilic Pseudo-Amino Acid Composition (APAAC) was proposed in (27,28). The definition of this set of features is similar to the PAAC descriptors. Using *H_1_(i)* and *H_2_(j)* as previously defined, the hydrophobicity and hydrophilicity correlation functions are defined as:

$\begin{matrix} H_{i,j}^{1}=H_{1}(i)H_{1}(j) \\ H_{i,j}^{2}=H_{2}(i)H_{2}(j) \end{matrix}$,

respectively. An illustrated example of the correlation functions is provided in the following **Figure 20**. Thus, sequence order factors can be defined as:

$$\begin{matrix} \tau_{1}=\frac{1}{N-1}\sum_{i=1}^{N-1} H_{i,i+1}^{1} \\ \tau_{2}=\frac{1}{N-1}\sum_{i=1}^{N-1} H_{i,i+1}^{2} \\ \tau_{3}=\frac{1}{N-2}\sum_{i=1}^{N-2} H_{i,i+2}^{1} \\ \tau_{4}=\frac{1}{N-2}\sum_{i=1}^{N-2} H_{i,i+2}^{2} \\ ... \\ \tau_{2\lambda-1}=\frac{1}{N-\lambda}\sum_{i=1}^{N-\lambda} H_{i,i+\lambda}^{1} \\ \tau_{2\lambda}=\frac{1}{N-\lambda}\sum_{i=1}^{N-\lambda} H_{i,i+\lambda}^{2} \end{matrix}$$

Then, Amphiphilic Pseudo-Amino Acid Composition (APAAC) is defined as:

$$\begin{matrix} P_{c}=\frac{f_{c}}{\sum_{r=1}^{20} f_{r}+w\sum_{j=1}^{2\lambda} \tau_{j}} , (1<c<20) \\ P_{c}=\frac{\omega\tau_{u}}{\sum_{r=1}^{20} f_{r}+w\sum_{j=1}^{2\lambda} \tau_{j}} , (21<u<20+2\lambda) \end{matrix}$$

where *w* is the weighting factor. In *iLearnPlus* this factor is set to *w* = 0.5 as described in Chou’s work (27). The PAAC and APAAC have been successfully applied to protein cellular attributes prediction (27) and enzyme subfamily classes prediction (28).


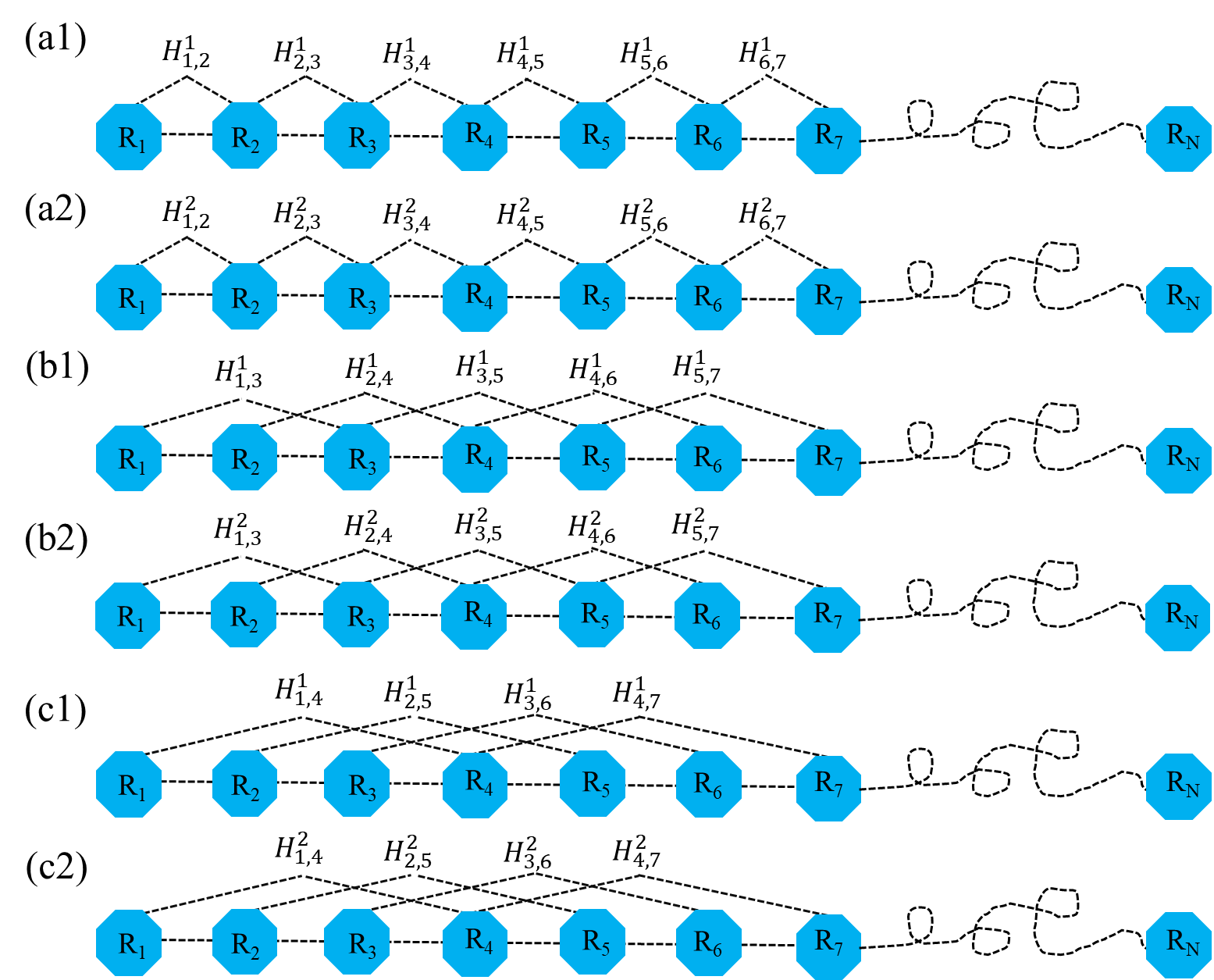


**Figure 20**. A schematic diagram to show (a1/a2) the first-rank, (b1/b2) the second-rank and (c1/c2) the third-rank sequence-order-coupling mode along a protein sequence through a hydrophobicity/hydrophilicity correlation function, where $H_{i,j}^{1}$and $H_{i,j}^{2}$ are given by the aforementioned equation. Panels (a1/a2) reflects the coupling mode between the most adjacent residues, panels (b1/b2) shows the coupling between the adjacent plus one residue, and panels (c1/c2) shows the coupling between the adjacent plus two residues. This figure is adapted from (28) for illustration purposes.

*Parameter(s)*:

- *Lambda value: integer, should be small than sequence length, default is 2.*
- *Weight value: weight factor, ranged from 0 to 1, default is 0.05.*

**AAindex**

Physicochemical properties of amino acids are the most intuitive features for representing biochemical reactions and have been extensively applied in bioinformatics research. The amino acid indices (AAindex) database (83) collects many published indices representing physicochemical properties of amino acids. For each physicochemical property, there is a set of 20 numerical values for all amino acids. Currently, 544 physicochemical properties can be retrieved from the AAindex database. After removing physicochemical properties with value 'NA' for any of the amino acids, 531 physicochemical properties were left. In contrast to the residue-based encoding methods of amino acid identity and evolutionary information, a vector of 531 mean values is used to represent a sample for various window sizes. The AAINDEX descriptor (35) can be applied to encode peptides of equal length.

*Parameters:*

- *Physicochemical properties for proteins: the names of used physicochemical amino acids indices.*

**BLOSUM62**

In this descriptor, the BLOSUM62 matrix is employed to represent the protein primary sequence information as the basic feature set. A matrix comprising of *m* × *n* elements is used to represent each residue in a training dataset, where *n* denotes the peptide length and *m*  =  20, which elements comprise 20 amino acids. Each row in the BLOSUM62 matrix is adopted to encode one of 20 amino acids. The BLOSUM62 descriptor can be applied to encode peptides of equal length. The BLOSUM62 descriptor has been successfully applied to ubiquitination site prediction (36).

**ZScale**

For this descriptor, each amino acid is characterized by five physicochemical descriptor variables (cf. **Table 14**), which were developed by Sandberg *et al.* in 1998 (94). The ZSCALE descriptor can be applied to encode peptides with equal length. The descriptor has been successfully applied to sumoylation site prediction (37).

**Table 14**. Z-scales for the 20 amino acids.

| **Amino acid** | **Z1** | **Z2** | **Z3** | **Z4** | **Z5** | **Amino Acid** | **Z1** | **Z2** | **Z3** | **Z4** | **Z5** |
| --- | --- | --- | --- | --- | --- | --- | --- | --- | --- | --- | --- |
| A | 0.24 | -2.32 | 0.60 | -0.14 | 1.30 | M | -2.85 | -0.22 | 0.47 | 1.94 | -0.98 |
| C | 0.84 | -1.67 | 3.71 | 0.18 | -2.65 | N | 3.05 | 1.60 | 1.04 | -1.15 | 1.61 |
| D | 3.98 | 0.93 | 1.93 | -2.46 | 0.75 | P | -1.66 | 0.27 | 1.84 | 0.70 | 2.00 |
| E | 3.11 | 0.26 | -0.11 | -3.04 | -0.25 | Q | 1.75 | 0.50 | -1.44 | -1.34 | 0.66 |
| F | -4.22 | 1.94 | 1.06 | 0.54 | -0.62 | R | 3.52 | 2.50 | -3.50 | 1.99 | -0.17 |
| G | 2.05 | 4.06 | 0.36 | -0.82 | -0.38 | S | 2.39 | -1.07 | 1.15 | -1.39 | 0.67 |
| H | 2.47 | 1.95 | 0.26 | 3.90 | 0.09 | T | 0.75 | -2.18 | -1.12 | -1.46 | -0.40 |
| I | -3.89 | -1.73 | -1.71 | -0.84 | 0.26 | V | -2.59 | -2.64 | -1.54 | -0.85 | -0.02 |
| K | 2.29 | 0.89 | -2.49 | 1.49 | 0.31 | W | -4.36 | 3.94 | 0.59 | 3.44 | -1.59 |
| L | -4.28 | -1.30 | -1.49 | -0.72 | 0.84 | Y | -2.54 | 2.44 | 0.43 | 0.04 | -1.47 |

**OPF_10bit**

For this descriptor, the amino acids are classified into 10 groups based their physicochemical properties (**Table 15**). Note that different groups might overlap, since a specific amino acid type may have two or more physicochemical properties. To reflect the correlation of different properties, a 10-bit vector were calculated to represent each amino acid of the N-terminus of peptide. Similarly, the position of the bit of this 10-bit vector is set to 1, if the amino acid belongs to a corresponding group and 0 otherwise. The OPF_10bit descriptor has been successfully applied to anti-cancer peptides prediction (80).

**Table 15.** Details of the division of the standard amino acid alphabet based on ten physicochemical properties.

| Rank | Physicochemical properties | Amino acid group |
| --- | --- | --- |
| 1 | Aromatic | {F, Y, W, H} |
| 2 | Negative | {D, E} |
| 3 | Positive | {K, H, R} |
| 4 | Polar | {N, Q, S, D, E, C, T, K, R, H, Y, W} |
| 5 | Hydrophobic | {A, G, C, T, I, V, L, K, H, F, Y, W, M} |
| 6 | Aliphatic | {I, V, L} |
| 7 | Tiny | {A, S, G, C} |
| 8 | Charged | {K, H, R, D, E} |
| 9 | Small | {P, N, D, T, C, A, G, S, V} |
| 10 | Proline | {P} |

**OPF_7bit type 1**

Similar to OPF_10bit descriptor, for this descriptor, the amino acids are classified into 7 groups. There are three subtypes of OPF_7bit descriptor (i.e. type 1, type 2 and type 3), due to different division of the amino acids. The difference of the type 1 to type 3 subtypes is the different division of amino acids. The division of amino acids for type 1 to 3 is listed in **Table 16**.

**KNN (K-Nearest Neighbors)**

The *K*-Nearest Neighbor for nucleotide (KNN) descriptor requires an extra training file and a label file. The training file is used to calculate the top *K*-Nearest Neighbor peptides by calculating the similarity score of two nucleotide sequences.

**ASDC (Adaptive skip dinucleotide composition)**

The adaptive skip dipeptide composition is a modified dipeptide composition, which sufficiently considers the correlation information present not only between adjacent residues but also between intervening residues (80). For given a sequence, the feature vector for ASDC is represented by:

$$ASDC=\left( f_{v1},f_{v1},\ldots,f_{v400} \right),$$

where *f_vi_* is calculated by

$$f_{vi}=\frac{\sum_{g=1}^{L-1} O_{i}^{g}}{\sum_{i=1}^{400} \sum_{g=1}^{L-1} O_{i}^{g}},$$

where *f_vi_* denotes the occurrence frequency of all possible dipeptide with ≤ *L*-1 intervening nucleotides. The ASDC descriptor has been successfully applied to anti-cancer peptide prediction (80) and cell-penetrating peptide prediction (81).

**Table 16**. Details of the division of the standard amino acid alphabet based on seven physicochemical properties.

| Rank | Physicochemical properties | Type 1 | Type 2 | Type 3 |
| --- | --- | --- | --- | --- |
| 1 | Hydrophobicity | {A, C, F, G, H, I, L, M, N, P, Q, S, T, V, W, Y} | {D, E} | {K, R} |
| 2 | Normalized Van der Waals volume | {C, F, I, L, M, V, W} | {A, G, H, P, S, T, Y} | {D, E, K, N, Q, R} |
| 3 | Polarity | {A, C, D, G, P, S, T} | {E, I, L, N, Q, V} | {F, H, K, M, R, W, Y} |
| 4 | Polarizibility | {C, F, I, L, M, V, W, Y} | {A, G, P, S, T} | {D, E, H, K, N, Q, R} |
| 5 | Charge | {A, D, G, S, T} | {C, E, I, L, N, P, Q, V} | {F, H, K, M, R, W, Y} |
| 6 | Secondary structures | {D, G, N, P, S} | {A, E, H, K, L, M, Q, R} | {C, F, I, T, V, W, Y} |
| 7 | Solvent accessibility | {A, C, F, G, I, L, V, W} | {H, M, P, S, T, Y} | {D, E, K, N, R, Q} |

**DistancePair (PseAAC of distance-pair and reduced alphabet)**

The descriptor incorporates the amino acid distance pair coupling information and the amino acid reduced alphabet profile into the general pseudo amino acid composition vector. For the reduced alphabet profile, they are cp(13), cp(14), and cp(15) as defined below:

cp(13) = {MF; IL;V;A;C;WYQHP;G;T; S;N;RK;D; E}

cp(14) = {EIMV; L;F;WY;G; P;C;A; S; T;N;HRKQ; E;D}

sp(15) = {P;G; E;K;R;Q;D; S;N; T;H;C; I;V;W;YF;A; L;M}

where the single letters without a semicolon (;) to separate them mean belonging to a same cluster. The DistancePair descriptor has been successfully applied to DNA-binding protein identification (16).

*Parameter(s):*

- *Maximum distance: the maximum distance value, default is 0.*
- *Reduced alphabet scheme: i.e. cp(13), cp(14), cp(15) or cp(20).*

**PseKRAAC (pseudo K-tuple reduced amino acids composition)**

Previous studies indicate that certain residues are similar in their physicochemical features, and can be clustered into groups because they play similar structural or functional roles in proteins (95). By implementing reduced amino acid alphabets, the protein complexity can be significantly simplified, which reduces information redundancy and decreases the risk of overfitting. The Pseudo *K*-tuple Reduced Amino Acids Composition (PseKRAAC) descriptor (29) includes two different feature types for protein sequence analysis: *g-gap* and *λ*-correlation PseKRAAC (27).

The *g-gap* PseKRAAC is used to represent a protein sequence with a vector containing RAAC*^K^* components, where *g* represents the gap between each *K*-tuple peptides (21,52,96,97). A *g*-*gap* of *n* reflects the sequence-order information for all *K*-tuple peptides with the starting residues separated by *n* residues. An illustrated example of this encoding scheme (*K* = 2) is provided in the following **Figure 21A**.

The *λ*-correlation PseKRAAC is used to represent a protein sequence with a vector containing RAAC*^K^* components, where *λ* is an integer that represents the correlation tier and is less than *N*-*K*, where *N* is the sequence length. The *n-*th-tier correlation factor (*λ* = *n*) reflects the sequence-order correlation between the *n*-th nearest residues. An illustrated example of this encoding scheme (*K* = 2) is provided in the following **Figure 21B**.


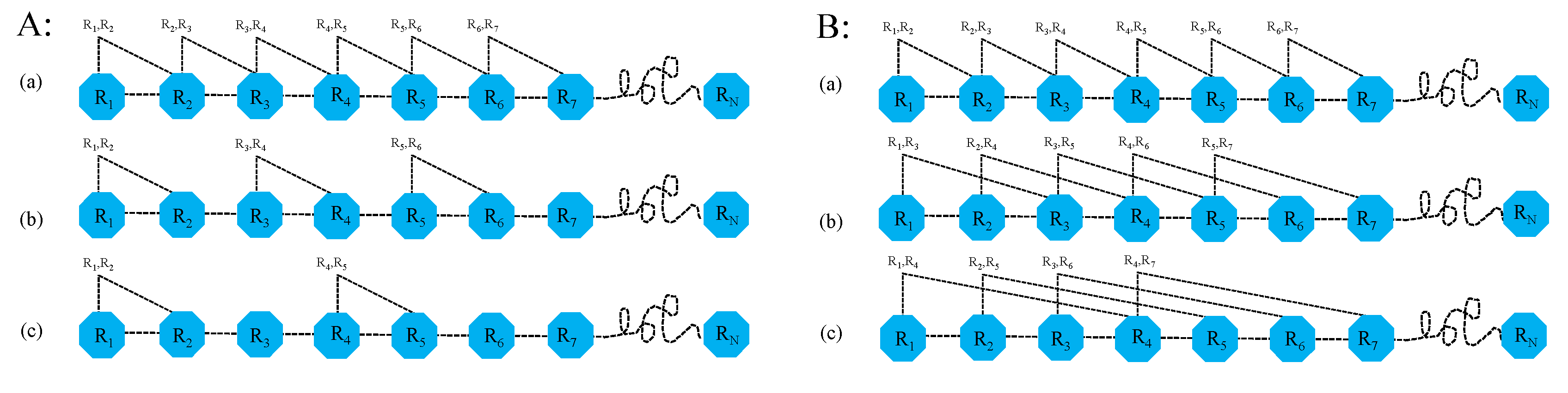


**Figure 21**. A schematic diagram showing: (A) *g-gap* definition of dipeptide, and (B) λ-correlation definition of dipeptide A: (a) *g-gap* of 0 reflects the sequence-order information between all adjacent dipeptides, i.e. separated by zero residues, (b) *g-gap* of 1 reflects the sequence-order information for all dipeptides with the starting residues separated by one residue, and (c) *g-gap* of 2 reflects the sequence-order information for all dipeptides with the starting residues separated by two residues; B: (a) the first-tier correlation factor reflects the sequence-order correlation between the nearest residues along a protein chain, (b) the second-tier correlation factor reflects the sequence-order correlation between the second nearest residues, (c) the third-tier correlation factor reflects the sequence-order correlation between the 3rd nearest residues, and so forth. The figure is adapted from (29).

The 16 types of reduced amino acid alphabets with different clustering approaches can be used to generate different versions of pseudo reduced amino acid compositions (PseRAACs) (**Table17**).

**Table 17**. A list of 16 types of reduced amino acid alphabets for proteins (29).

| Type | Description | Cluster | Reference |
| --- | --- | --- | --- |
| 1 | RedPSSM | 2-19 | (98) |
| 2 | BLOSUM 62 matrix | 2-6, 8, 15 | (99) |
| 3 | PAM matrix (3A) and WAG matrix (3B) | 2-19 | (100) |
| 4 | Protein Blocks | 5,8,9,11,13 | (101) |
| 5 | BLOSUM50 matrix | 3,4,8,10,15 | (101) |
| 6 | Multiple cluster | 4,5A,5B,5C | (102) |
| 7 | Metric multi-dimensional scaling | 2-19 | (103) |
| 8 | Grantham Distance Matrix | 2-19 | (104) |
| 9 | Grantham Distance Matrix | 2-19 | (104) |
| 10 | BLOSUM matrix for SWISS-PROT | 2-19 | (105) |
| 11 | BLOSUM matrix for SWISS-PROT | 2-19 | (105) |
| 12 | BLOSUM matrix for DAPS | 2-18 | (106) |
| 13 | Coarse-graining substitution  matrices | 4,12,17 | (107) |
| 14 | Alphabet Simplifer | 2-19 | (108) |
| 15 | MJ matrix | 2-16 | (109) |
| 16 | BLOSUM50 matrix | 2-16 | (109) |

*Parameter(s):*

- *RAAC subtype model: RAAC subtype model, i.e. g-gap or lambda-correlation.*
- *Gap value: the gap value for between two amino acids.*
- *Lambda value: the lambda value in lambda-correlation model.*
- *K-tuple: k-tuple value.*
- *RAAC cluster: the RAAC cluster type for each type of feature descriptor.*

# 12. Commonly Used Feature Descriptors and Parameters for Ligands

**Bask**

The feature descriptor calculates some commonly used bask information index based on its topological structure, and 21 molecular connectivity can be obtained for this feature descriptor.

**Burden**

The feature descriptor calculates 64 burden eigenvalue descriptors.

**Phamacophore**

The feature descriptor calculates potential pharmacophore point descriptors definitions as describes in Pharmacophores and Pharmacophore Searches 2006 (Eds. T. Langer and R.D. Hoffmann), Chapter 3: Alignment-free Pharmacophore Patterns - A Correlation-vector Approach.

**Constitution**

The molecular constitution descriptors characterize composition of chemical elements and chemical bonds, path length, hydrogen bond-acceptor, and donator in the constitution module.

**Topology**

The topological descriptors, which are calculated directly from the ligand structure, quantify key topological aspects including molecular connectivity and valence connectivity for different path-orders, cycle, or cluster size.

**Connectivity**

The feature descriptor calculates 44 molecular connectivity indices based on its topological

structure.

**Kappa**

The feature descriptor calculates 7 descriptors of Kier and Hall's kappa indices based on its topological structure.

**E-state**

The feature descriptor calculates the E-state fingerprints and values based on Kier and Hall's paper.

**Autocorrelation-moran**

The feature descriptor calculates 32 moran autocorrelation descriptors.

**Autocorrelation-geary**

The feature descriptor calculates 32 geary autocorrelation descriptors based on its topological

structure.

**Autocorrelation-broto**

The feature descriptor calculates 32 Moreau-Broto autocorrelation descriptors.

**Molecular properties**

The feature descriptor calculates Molecular physical/chemical properties based on some special

type of approaches, including: LogP; LogP2; MR; TPSA, UI and Hy.

**Charge**

The feature descriptor calculates Charge descriptors based on Gasteiger/Marseli partial charges.

**Moe-type**

The MOE-type descriptors are computed from the connection table information based on atomic contributions to Van der Waals surface area, LogP, molar refractivity, partial charge, and E-state value.

**Fingerprints**

Molecular fingerprints are string representations of chemical structures, which consist of bins, each bin being a substructure descriptor associated with a specific molecular feature. 3 types of molecular fingerprints are provided, including E-state fingerprints, MACCS fingerprints, and Morgan fingerprints.

# 13. Commonly Used Feature Descriptors and Parameters for Protein Structure

**AAC type 1 and 2**

Amino Acids Content descriptor calculate the frequency of each amino acid type in the microenvironments of a target site. The microenvironments of the target site are defined by a three-dimensional position and a radius defining the neighborhood. Shells are formed around each target site. And the frequency of each amino acid type is calculated for each shell (AAC_type1) and for cumulative shells (AAC_type2). **Figure 22 s**hows an example. The solid dot represents the target site, while the cycles denote other residues. The microenvironment of the target site is specified as a three-dimensional position and a radius defining the neighborhood. And shells are formed around the site (The yellow part is the first shell, while the green part form the second shell, and the blue part is the third part). For AAC_type1 feature descriptor, the frequency of amino acids in the first shell (i.e. residue #1), the second shell (i.e. residue #2, #3 and #4) and the third shell (i.e. residue #5-9) will be calculated, respectively. For AAC_type2 descriptor, at first the frequency of amino acids in the first shell (i.e. residue #1) is calculated. Then, the frequency of amino acids in the first and second shells (i.e. residue #1-4) will be calculated. Finally, the frequency of amino acids in all the three shells (i.e. residue #1-9) will be calculated.


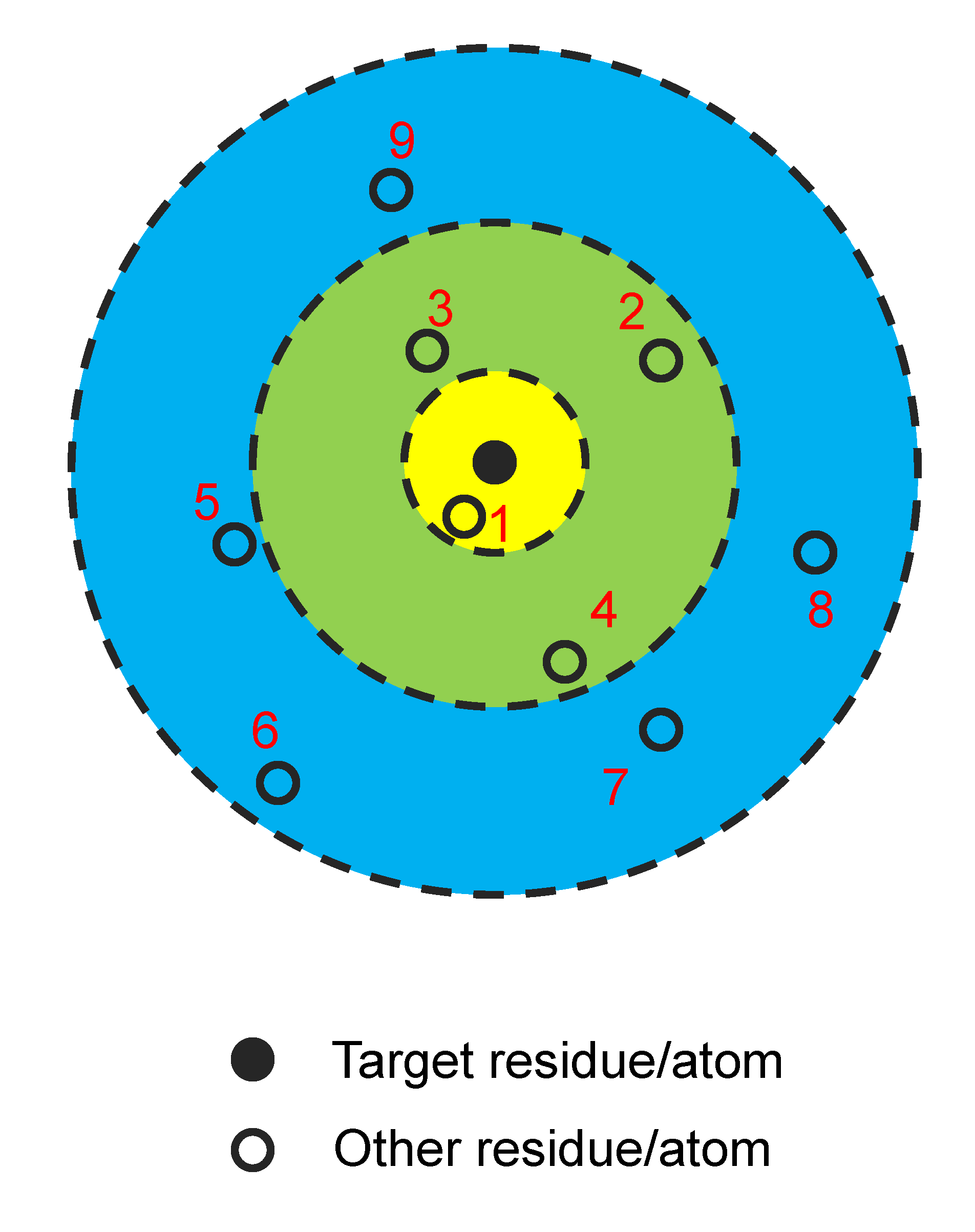


**Figure 22**. An example of feature extraction for protein structure “AAC_type1/2” descriptors.

*Parameter(s):*

- *Shell from: the start radius (i.e. the radius of first shell), default is 3.*
- *Shell to: the maximum radius of the shell, default is 20.*
- *Step: the interval between two adjacent shells, default is 3.*

**GAAC type 1 and 2**

In the GAAC encoding, the 20 amino acid types are further categorized into five classes according to their physicochemical properties, e.g. hydrophobicity, charge and molecular size (110). The five classes include the aliphatic group (*g1*: GAVLMI), aromatic group (*g2*: FYW), positive charge group (*g3*: KRH), negative charged group (*g4*: DE) and uncharged group (*g5*: STCPNQ). And the frequency of each amino acid group is calculated for each shell (GAAC_type1) and for cumulative shells (GAAC_type2).

*Parameter(s):*

- *Shell from: the start radius (i.e. the radius of first shell), default is 3.*
- *Shell to: the maximum radius of the shell, default is 20.*
- *Step: the interval between two adjacent shells, default is 3.*

**SS3 and SS8**

The “secondary structure” feature group encodes multiple features based on secondary structural elements around a target residue. For feature sets in this group, “SS3” considers three types of secondary structural elements (i.e. helix, β-strand, and β-turns and -loops(111)), while “SS8” considers eight types of secondary structural elements (i.e. α-helix, isolated β-bridge residue, strand, 3-10 helix, Π-helix, turn, bend and other(111)). The type1 features calculate the frequency of each type of SSE in each shell (i.e. SS3_type1 and SS8_type1) while type2 features quantify the frequency of each SSE type in cumulative shells (i.e. SS3_type2 and SS8_type2)

*Parameter(s):*

- *Shell from: the start radius (i.e. the radius of first shell), default is 3.*
- *Shell to: the maximum radius of the shell, default is 20.*
- *Step: the interval between two adjacent shells, default is 3.*

**HSE_CA and HSE_CB**

HSE calculates the half sphere exposure and residue depth as descriptors for each residue in a given structure. Half sphere exposure (HSE) is a 2D measure of solvent exposure which counts the number of Cα atoms around a residue in the direction of its side chain and in the opposite direction (within a radius of 13 Å)(112). HSE compliments the residue depth measure(113), which is calculated as an average distance of a residue’s atoms from the solvent accessible surface(114). HSE comes in two flavors: HSEα (HSE_CA) and HSEβ (HSE_CB). The former only uses the Cα atom positions, while the latter uses the Cα and Cβ atom positions.

**Residue depth**

The residue depth descriptor is the average distance of a residue’s atoms from the solvent accessible surface.

**AC_type1 and AC_type2**

The “atom composition” feature group includes two types of encoding schemes that are computed at the atomic level. For the feature sets in this group, the target atom is specified as a three-dimensional position and a radius defining its neighborhood, and shells are formed around each target atom (**Figure 22**). Then, frequency of each atom type is calculated in each shell (i.e. AC_type1) and in cumulative shells (i.e. AC_type2).

*Parameter(s):*

- *Shell from: the start radius (i.e. the radius of first shell), default is 1.*
- *Shell to: the maximum radius of the shell, default is 10.*
- *Step: the interval between two adjacent shells, default is 1.*

**Network-based index**

The “network-based index” feature set encodes residues in the context of a network that represents placement of residues in the space defined by the protein structure. These features quantify the degree, degree centrality, betweenness, clustering coefficient, closeness and centrality values.

# 14. References

1. Berman, H.M., Westbrook, J., Feng, Z., Gilliland, G., Bhat, T.N., Weissig, H., Shindyalov, I.N. and Bourne, P.E. (2000) The Protein Data Bank. *Nucleic Acids Res*, **28**, 235-242.

2. Bhasin, M. and Raghava, G.P. (2004) Classification of nuclear receptors based on amino acid composition and dipeptide composition. *J Biol Chem*, **279**, 23262-23266.

3. Zhou, C., Wang, C., Liu, H., Zhou, Q., Liu, Q., Guo, Y., Peng, T., Song, J., Zhang, J., Chen, L. *et al.* (2018) Identification and analysis of adenine N(6)-methylation sites in the rice genome. *Nat Plants*, **4**, 554-563.

4. Chen, Z., Zhao, P., Li, F., Marquez-Lago, T.T., Leier, A., Revote, J., Zhu, Y., Powell, D.R., Akutsu, T., Webb, G.I. *et al.* (2020) iLearn: an integrated platform and meta-learner for feature engineering, machine-learning analysis and modeling of DNA, RNA and protein sequence data. *Brief Bioinform*, **21**, 1047-1057.

5. Chen, K., Jiang, Y., Du, L. and Kurgan, L. (2009) Prediction of integral membrane protein type by collocated hydrophobic amino acid pairs. *J Comput Chem*, **30**, 163-172.

6. Chen, K., Kurgan, L.A. and Ruan, J. (2007) Prediction of flexible/rigid regions from protein sequences using k-spaced amino acid pairs. *BMC Struct Biol*, **7**, 25.

7. Saravanan, V. and Gautham, N. (2015) Harnessing Computational Biology for Exact Linear B-Cell Epitope Prediction: A Novel Amino Acid Composition-Based Feature Descriptor. *OMICS*, **19**, 648-658.

8. Cai, C.Z., Han, L.Y., Ji, Z.L., Chen, X. and Chen, Y.Z. (2003) SVM-Prot: Web-based support vector machine software for functional classification of a protein from its primary sequence. *Nucleic Acids Res*, **31**, 3692-3697.

9. Cai, C.Z., Han, L.Y., Ji, Z.L. and Chen, Y.Z. (2004) Enzyme family classification by support vector machines. *Proteins*, **55**, 66-76.

10. Dubchak, I., Muchnik, I., Holbrook, S.R. and Kim, S.H. (1995) Prediction of protein folding class using global description of amino acid sequence. *Proc Natl Acad Sci U S A*, **92**, 8700-8704.

11. Dubchak, I., Muchnik, I., Mayor, C., Dralyuk, I. and Kim, S.H. (1999) Recognition of a protein fold in the context of the Structural Classification of Proteins (SCOP) classification. *Proteins*, **35**, 401-407.

12. Han, L.Y., Cai, C.Z., Lo, S.L., Chung, M.C. and Chen, Y.Z. (2004) Prediction of RNA-binding proteins from primary sequence by a support vector machine approach. *RNA*, **10**, 355-368.

13. Shen, J., Zhang, J., Luo, X., Zhu, W., Yu, K., Chen, K., Li, Y. and Jiang, H. (2007) Predicting protein-protein interactions based only on sequences information. *Proc Natl Acad Sci U S A*, **104**, 4337-4341.

14. Qiang, X., Chen, H., Ye, X., Su, R. and Wei, L. (2018) 【m6A - predictor - muti-species】M6AMRFS: Robust Prediction of N6-Methyladenosine Sites With Sequence-Based Features in Multiple Species. *Front Genet*, **9**, 495.

15. Liu, B., Xu, J., Lan, X., Xu, R., Zhou, J., Wang, X. and Chou, K.C. (2014) iDNA-Prot|dis: identifying DNA-binding proteins by incorporating amino acid distance-pairs and reduced alphabet profile into the general pseudo amino acid composition. *PLoS One*, **9**, e106691.

16. Liu, B., Gao, X. and Zhang, H. (2019) BioSeq-Analysis2.0: an updated platform for analyzing DNA, RNA and protein sequences at sequence level and residue level based on machine learning approaches. *Nucleic Acids Res*, **47**, e127.

17. Feng, Z.P. and Zhang, C.T. (2000) Prediction of membrane protein types based on the hydrophobic index of amino acids. *J Protein Chem*, **19**, 269-275.

18. Lin, Z. and Pan, X.M. (2001) Accurate prediction of protein secondary structural content. *J Protein Chem*, **20**, 217-220.

19. Sokal, R.R. and Thomson, B.A. (2006) Population structure inferred by local spatial autocorrelation: an example from an Amerindian tribal population. *Am J Phys Anthropol*, **129**, 121-131.

20. Horne, D.S. (1988) Prediction of protein helix content from an autocorrelation analysis of sequence hydrophobicities. *Biopolymers*, **27**, 451-477.

21. Liu, B., Liu, F., Fang, L., Wang, X. and Chou, K.C. (2015) repDNA: a Python package to generate various modes of feature vectors for DNA sequences by incorporating user-defined physicochemical properties and sequence-order effects. *Bioinformatics*, **31**, 1307-1309.

22. Dong, Q., Zhou, S. and Guan, J. (2009) A new taxonomy-based protein fold recognition approach based on autocross-covariance transformation. *Bioinformatics*, **25**, 2655-2662.

23. Guo, Y., Yu, L., Wen, Z. and Li, M. (2008) Using support vector machine combined with auto covariance to predict protein-protein interactions from protein sequences. *Nucleic Acids Res*, **36**, 3025-3030.

24. Chou, K.C. (2000) Prediction of protein subcellular locations by incorporating quasi-sequence-order effect. *Biochem Biophys Res Commun*, **278**, 477-483.

25. Chou, K.C. and Cai, Y.D. (2004) Prediction of protein subcellular locations by GO-FunD-PseAA predictor. *Biochem Biophys Res Commun*, **320**, 1236-1239.

26. Schneider, G. and Wrede, P. (1994) The rational design of amino acid sequences by artificial neural networks and simulated molecular evolution: de novo design of an idealized leader peptidase cleavage site. *Biophys J*, **66**, 335-344.

27. Chou, K.C. (2001) Prediction of protein cellular attributes using pseudo-amino acid composition. *Proteins*, **43**, 246-255.

28. Chou, K.C. (2005) Using amphiphilic pseudo amino acid composition to predict enzyme subfamily classes. *Bioinformatics*, **21**, 10-19.

29. Zuo, Y., Li, Y., Chen, Y., Li, G., Yan, Z. and Yang, L. (2017) PseKRAAC: a flexible web server for generating pseudo K-tuple reduced amino acids composition. *Bioinformatics*, **33**, 122-124.

30. Chen, Z., Chen, Y.Z., Wang, X.F., Wang, C., Yan, R.X. and Zhang, Z. (2011) Prediction of ubiquitination sites by using the composition of k-spaced amino acid pairs. *PLoS One*, **6**, e22930.

31. Chen, Z., Zhou, Y., Song, J. and Zhang, Z. (2013) hCKSAAP_UbSite: improved prediction of human ubiquitination sites by exploiting amino acid pattern and properties. *Biochim Biophys Acta*, **1834**, 1461-1467.

32. Wang, J.T.L., Ma, Q., Shasha, D. and Wu, C.H. (2001) New techniques for extracting features from protein sequences. **40**, 426–441.

33. White, G. and Seffens, W. (1998) Using a neural network to backtranslate amino acid sequences. *Electronic Journal of Biotechnology (ISSN: 0717-3458) Vol 1 Num 3*, **1**.

34. Lin, K., May, A.C. and Taylor, W.R. (2002) Amino acid encoding schemes from protein structure alignments: multi-dimensional vectors to describe residue types. *J Theor Biol*, **216**, 361-365.

35. Tung, C.W. and Ho, S.Y. (2008) Computational identification of ubiquitylation sites from protein sequences. *BMC Bioinformatics*, **9**, 310.

36. Lee, T.Y., Chen, S.A., Hung, H.Y. and Ou, Y.Y. (2011) Incorporating distant sequence features and radial basis function networks to identify ubiquitin conjugation sites. *PLoS One*, **6**, e17331.

37. Chen, Y.Z., Chen, Z., Gong, Y.A. and Ying, G. (2012) SUMOhydro: a novel method for the prediction of sumoylation sites based on hydrophobic properties. *PLoS One*, **7**, e39195.

38. Chen, X., Qiu, J.D., Shi, S.P., Suo, S.B., Huang, S.Y. and Liang, R.P. (2013) Incorporating key position and amino acid residue features to identify general and species-specific Ubiquitin conjugation sites. *Bioinformatics*, **29**, 1614-1622.

39. Lee, D., Karchin, R. and Beer, M.A. (2011) Discriminative prediction of mammalian enhancers from DNA sequence. *Genome Res*, **21**, 2167-2180.

40. Noble, W.S., Kuehn, S., Thurman, R., Yu, M. and Stamatoyannopoulos, J. (2005) Predicting the in vivo signature of human gene regulatory sequences. *Bioinformatics*, **21 Suppl 1**, i338-343.

41. Gupta, S., Dennis, J., Thurman, R.E., Kingston, R., Stamatoyannopoulos, J.A. and Noble, W.S. (2008) Predicting human nucleosome occupancy from primary sequence. *PLoS Comput Biol*, **4**, e1000134.

42. Chen, W., Tran, H., Liang, Z., Lin, H. and Zhang, L. (2015) Identification and analysis of the N(6)-methyladenosine in the Saccharomyces cerevisiae transcriptome. *Sci Rep*, **5**, 13859.

43. Qiang, X., Chen, H., Ye, X., Su, R. and Wei, L. (2018) M6AMRFS: Robust Prediction of N6-Methyladenosine Sites With Sequence-Based Features in Multiple Species. *Front Genet*, **9**, 495.

44. Gao, F. and Zhang, C.T. (2004) Comparison of various algorithms for recognizing short coding sequences of human genes. *Bioinformatics*, **20**, 673-681.

45. Doench, J.G., Fusi, N., Sullender, M., Hegde, M., Vaimberg, E.W., Donovan, K.F., Smith, I., Tothova, Z., Wilen, C., Orchard, R. *et al.* (2016) Optimized sgRNA design to maximize activity and minimize off-target effects of CRISPR-Cas9. *Nat Biotechnol*, **34**, 184-191.

46. Cursons, J., Pillman, K.A., Scheer, K.G., Gregory, P.A., Foroutan, M., Hediyeh-Zadeh, S., Toubia, J., Crampin, E.J., Goodall, G.J., Bracken, C.P. *et al.* (2018) Combinatorial Targeting by MicroRNAs Co-ordinates Post-transcriptional Control of EMT. *Cell Syst*, **7**, 77-91 e77.

47. He, W., Jia, C. and Zou, Q. (2019) 4mCPred: machine learning methods for DNA N4-methylcytosine sites prediction. *Bioinformatics*, **35**, 593-601.

48. Lalović, D. and Veljković, V. (1990) The global average DNA base composition of coding regions may be determined by the electron-ion interaction potential. *Biosystems*, **23**, 311-316.

49. Nair, A.S. and Sreenadhan, S.P. (2006) A coding measure scheme employing electron-ion interaction pseudopotential (EIIP). *Bioinformation*, **1**, 197-202.

50. Manavalan, B., Basith, S., Shin, T.H., Lee, D.Y., Wei, L. and Lee, G. (2019) 4mCpred-EL: An Ensemble Learning Framework for Identification of DNA N(4)-methylcytosine Sites in the Mouse Genome. *Cells*, **8**.

51. Wei, L., Su, R., Luan, S., Liao, Z., Manavalan, B., Zou, Q. and Shi, X. (2019) Iterative feature representations improve N4-methylcytosine site prediction. *Bioinformatics*, **35**, 4930-4937.

52. Liu, B., Liu, F., Wang, X., Chen, J., Fang, L. and Chou, K.C. (2015) Pse-in-One: a web server for generating various modes of pseudo components of DNA, RNA, and protein sequences. *Nucleic Acids Res*, **43**, W65-71.

53. Dong, J., Yao, Z.J., Wen, M., Zhu, M.F., Wang, N.N., Miao, H.Y., Lu, A.P., Zeng, W.B. and Cao, D.S. (2016) BioTriangle: a web-accessible platform for generating various molecular representations for chemicals, proteins, DNAs/RNAs and their interactions. *J Cheminform*, **8**, 34.

54. Dong, J., Yao, Z.J., Zhang, L., Luo, F., Lin, Q., Lu, A.P., Chen, A.F. and Cao, D.S. (2018) PyBioMed: a python library for various molecular representations of chemicals, proteins and DNAs and their interactions. *J Cheminform*, **10**, 16.

55. Jain, A.K., Murty, M.N. and Flynn, P.J. (1999) Data clustering: A review. *Acm Computing Surveys*, **31**, 264-323.

56. Rokach, L. and Maimon, O. (2005) In Maimon, O. and Rokach, L. (eds.), *Data Mining and Knowledge Discovery Handbook*. Springer US, Boston, MA, pp. 321-352.

57. Theodoridis, S. and Koutroumbas, K. (2009) In Theodoridis, S. and Koutroumbas, K. (eds.), *Pattern Recognition (Fourth Edition)*. Academic Press, Boston, pp. 653-700.

58. Filippone, M., Camastra, F., Masulli, F. and Rovetta, S. (2008) A survey of kernel and spectral methods for clustering. *Pattern Recognition*, **41**, 176-190.

59. Enright, A.J., Van Dongen, S. and Ouzounis, C.A. (2002) An efficient algorithm for large-scale detection of protein families. *Nucleic Acids Res*, **30**, 1575-1584.

60. Jain, A.K. (2010) Data clustering: 50 years beyond K-means. *Pattern Recognition Letters*, **31**, 651-666.

61. Frey, B.J. and Dueck, D. (2007) Clustering by passing messages between data points. *Science*, **315**, 972-976.

62. Cheng, Y.Z. (1995) Mean Shift, Mode Seeking, and Clustering. *Ieee Transactions on Pattern Analysis and Machine Intelligence*, **17**, 790-799.

63. Ester, M., Kriegel, H.-P., Sander, r. and Xu, X. (1996), *Proceedings of the Second International Conference on Knowledge Discovery and Data Mining*. AAAI Press, Portland, Oregon, pp. 226-231.

64. Pearson, K. (1901) LIII. On lines and planes of closest fit to systems of points in space. *The London, Edinburgh, and Dublin Philosophical Magazine and Journal of Science*, **2**, 559-572.

65. Blei, D.M., Ng, A.Y. and Jordan, M.I. (2003) Latent dirichlet allocation. **3**, 993–1022.

66. Maaten, L.V.D. (2014) Accelerating t-SNE using tree-based algorithms. *J. Mach. Learn. Res.*, **15**, 3221-3245.

67. Sun, L., Luo, H., Bu, D., Zhao, G., Yu, K., Zhang, C., Liu, Y., Chen, R. and Zhao, Y. (2013) Utilizing sequence intrinsic composition to classify protein-coding and long non-coding transcripts. *Nucleic Acids Res*, **41**, e166.

68. Wang, L., Park, H.J., Dasari, S., Wang, S., Kocher, J.P. and Li, W. (2013) CPAT: Coding-Potential Assessment Tool using an alignment-free logistic regression model. *Nucleic Acids Res*, **41**, e74.

69. Leslie, C.S., Eskin, E., Cohen, A., Weston, J. and Noble, W.S. (2004) Mismatch string kernels for discriminative protein classification. *Bioinformatics*, **20**, 467-476.

70. El-Manzalawy, Y., Dobbs, D. and Honavar, V. (2008) Predicting flexible length linear B-cell epitopes. *Comput Syst Bioinformatics Conf*, **7**, 121-132.

71. Luo, L., Li, D., Zhang, W., Tu, S., Zhu, X. and Tian, G. (2016) Accurate Prediction of Transposon-Derived piRNAs by Integrating Various Sequential and Physicochemical Features. *PLoS One*, **11**, e0153268.

72. Chen, Z., Zhao, P., Li, F., Wang, Y., Smith, A.I., Webb, G.I., Akutsu, T., Baggag, A., Bensmail, H. and Song, J. (2020) Comprehensive review and assessment of computational methods for predicting RNA post-transcriptional modification sites from RNA sequences. *Brief Bioinform*, **21**, 1676-1696.

73. He, X., Zhang, S., Zhang, Y., Jiang, T. and Zeng, J. (2017) Characterizing RNA Pseudouridylation by Convolutional Neural Networks. *bioRxiv*, 126979.

74. Alipanahi, B., Delong, A., Weirauch, M.T. and Frey, B.J. (2015) Predicting the sequence specificities of DNA- and RNA-binding proteins by deep learning. *Nat Biotechnol*, **33**, 831-838.

75. Zhou, J. and Troyanskaya, O.G. (2015) Predicting effects of noncoding variants with deep learning-based sequence model. *Nat Methods*, **12**, 931-934.

76. Liu, Q., Chen, J., Wang, Y., Li, S., Jia, C., Song, J. and Li, F. (2020) DeepTorrent: a deep learning-based approach for predicting DNA N4-methylcytosine sites. *Brief Bioinform*.

77. Feng, S., Liang, Y., Du, W., Lv, W. and Li, Y. (2020) LncLocation: Efficient Subcellular Location Prediction of Long Non-Coding RNA-Based Multi-Source Heterogeneous Feature Fusion. *Int J Mol Sci*, **21**.

78. Dou, L., Li, X., Ding, H., Xu, L. and Xiang, H. (2020) Prediction of m5C Modifications in RNA Sequences by Combining Multiple Sequence Features. *Mol Ther Nucleic Acids*, **21**, 332-342.

79. Tang, Q., Nie, F., Kang, J. and Chen, W. (2020) ncPro-ML: An integrated computational tool for identifying non-coding RNA promoters in multiple species. *Comput Struct Biotechnol J*, **18**, 2445-2452.

80. Wei, L., Zhou, C., Chen, H., Song, J. and Su, R. (2018) ACPred-FL: a sequence-based predictor using effective feature representation to improve the prediction of anti-cancer peptides. *Bioinformatics*, **34**, 4007-4016.

81. Wei, L., Tang, J. and Zou, Q. (2017) SkipCPP-Pred: an improved and promising sequence-based predictor for predicting cell-penetrating peptides. *BMC Genomics*, **18**, 742.

82. Pan, G., Jiang, L., Tang, J. and Guo, F. (2018) A Novel Computational Method for Detecting DNA Methylation Sites with DNA Sequence Information and Physicochemical Properties. *Int J Mol Sci*, **19**.

83. Kawashima, S., Pokarowski, P., Pokarowska, M., Kolinski, A., Katayama, T. and Kanehisa, M. (2008) AAindex: amino acid index database, progress report 2008. *Nucleic Acids Res*, **36**, D202-205.

84. Chen, W., Feng, P.M., Lin, H. and Chou, K.C. (2013) iRSpot-PseDNC: identify recombination spots with pseudo dinucleotide composition. *Nucleic Acids Res*, **41**, e68.

85. Chen, W., Lei, T.Y., Jin, D.C., Lin, H. and Chou, K.C. (2014) PseKNC: a flexible web server for generating pseudo K-tuple nucleotide composition. *Anal Biochem*, **456**, 53-60.

86. Qiu, W.R., Xiao, X. and Chou, K.C. (2014) iRSpot-TNCPseAAC: identify recombination spots with trinucleotide composition and pseudo amino acid components. *Int J Mol Sci*, **15**, 1746-1766.

87. He, W., Jia, C., Duan, Y. and Zou, Q. (2018) 70ProPred: a predictor for discovering sigma70 promoters based on combining multiple features. *BMC Syst Biol*, **12**, 44.

88. He, W., Jia, C. and Zou, Q. (2018) 4mCPred: Machine Learning Methods for DNA N4-methylcytosine sites Prediction. *Bioinformatics*.

89. Chen, K., Kurgan, L. and Rahbari, M. (2007) Prediction of protein crystallization using collocation of amino acid pairs. *Biochem Biophys Res Commun*, **355**, 764-769.

90. Chen, K., Kurgan, L.A. and Ruan, J. (2008) Prediction of protein structural class using novel evolutionary collocation-based sequence representation. *J Comput Chem*, **29**, 1596-1604.

91. Rao, H.B., Zhu, F., Yang, G.B., Li, Z.R. and Chen, Y.Z. (2011) Update of PROFEAT: a web server for computing structural and physicochemical features of proteins and peptides from amino acid sequence. *Nucleic Acids Res*, **39**, W385-390.

92. Tomii, K. and Kanehisa, M. (1996) Analysis of amino acid indices and mutation matrices for sequence comparison and structure prediction of proteins. *Protein Eng*, **9**, 27-36.

93. Grantham, R. (1974) Amino acid difference formula to help explain protein evolution. *Science*, **185**, 862-864.

94. Sandberg, M., Eriksson, L., Jonsson, J., Sjöström, M. and Wold, S. (1998) New chemical descriptors relevant for the design of biologically active peptides. A multivariate characterization of 87 amino acids. *J Med Chem*, **41**, 2481-2491.

95. Wang, J. and Wang, W. (1999) A computational approach to simplifying the protein folding alphabet. *Nat Struct Biol*, **6**, 1033-1038.

96. Liu, B., Fang, L., Wang, S., Wang, X., Li, H. and Chou, K.C. (2015) Identification of microRNA precursor with the degenerate K-tuple or Kmer strategy. *J Theor Biol*, **385**, 153-159.

97. Wang, L., Wang, Y. and Chang, Q. (2016) Feature selection methods for big data bioinformatics: A survey from the search perspective. *Methods*, **111**, 21-31.

98. Liang, Y., Liu, S. and Zhang, S. (2015) Prediction of Protein Structural Classes for Low-Similarity Sequences Based on Consensus Sequence and Segmented PSSM. *Comput Math Methods Med*, **2015**, 370756.

99. Ogul, H. and Mumcuoglu, E.U. (2007) A discriminative method for remote homology detection based on n-peptide compositions with reduced amino acid alphabets. *Biosystems*, **87**, 75-81.

100. Kosiol, C., Goldman, N. and Buttimore, N.H. (2004) A new criterion and method for amino acid classification. *J Theor Biol*, **228**, 97-106.

101. Zuo, Y.C. and Li, Q.Z. (2010) Using K-minimum increment of diversity to predict secretory proteins of malaria parasite based on groupings of amino acids. *Amino Acids*, **38**, 859-867.

102. Melo, F. and Marti-Renom, M.A. (2006) Accuracy of sequence alignment and fold assessment using reduced amino acid alphabets. *Proteins*, **63**, 986-995.

103. Rakshit, S. and Ananthasuresh, G.K. (2008) An amino acid map of inter-residue contact energies using metric multi-dimensional scaling. *J Theor Biol*, **250**, 291-297.

104. Susko, E. and Roger, A.J. (2007) On reduced amino acid alphabets for phylogenetic inference. *Mol Biol Evol*, **24**, 2139-2150.

105. Liu, X., Liu, D., Qi, J. and Zheng, W.M. (2002) Simplified amino acid alphabets based on deviation of conditional probability from random background. *Phys Rev E Stat Nonlin Soft Matter Phys*, **66**, 021906.

106. Li, J. and Wang, W. (2007) Grouping of amino acids and recognition of protein structurally conserved regions by reduced alphabets of amino acids. *Sci China C Life Sci*, **50**, 392-402.

107. Peterson, E.L., Kondev, J., Theriot, J.A. and Phillips, R. (2009) Reduced amino acid alphabets exhibit an improved sensitivity and selectivity in fold assignment. *Bioinformatics*, **25**, 1356-1362.

108. Cannata, N., Toppo, S., Romualdi, C. and Valle, G. (2002) Simplifying amino acid alphabets by means of a branch and bound algorithm and substitution matrices. *Bioinformatics*, **18**, 1102-1108.

109. Li, T., Fan, K., Wang, J. and Wang, W. (2003) Reduction of protein sequence complexity by residue grouping. *Protein Eng*, **16**, 323-330.

110. Lee, T.Y., Lin, Z.Q., Hsieh, S.J., Bretana, N.A. and Lu, C.T. (2011) Exploiting maximal dependence decomposition to identify conserved motifs from a group of aligned signal sequences. *Bioinformatics*, **27**, 1780-1787.

111. Kabsch, W. and Sander, C. (1983) Dictionary of protein secondary structure: pattern recognition of hydrogen-bonded and geometrical features. *Biopolymers*, **22**, 2577-2637.

112. Cock, P.J., Antao, T., Chang, J.T., Chapman, B.A., Cox, C.J., Dalke, A., Friedberg, I., Hamelryck, T., Kauff, F., Wilczynski, B. *et al.* (2009) Biopython: freely available Python tools for computational molecular biology and bioinformatics. *Bioinformatics*, **25**, 1422-1423.

113. Song, J., Tan, H., Takemoto, K. and Akutsu, T. (2008) HSEpred: predict half-sphere exposure from protein sequences. *Bioinformatics*, **24**, 1489-1497.

114. Sanner, M.F., Olson, A.J. and Spehner, J.C. (1996) Reduced surface: an efficient way to compute molecular surfaces. *Biopolymers*, **38**, 305-320.
